# High-throughput Identification and Characterization of Two-dimensional Materials using Density functional theory

**DOI:** 10.1038/s41598-017-05402-0

**Published:** 2017-07-12

**Authors:** Kamal Choudhary, Irina Kalish, Ryan Beams, Francesca Tavazza

**Affiliations:** Materials Science and Engineering Division, National Institute of Standards and Technology, Gaithersburg, MD 20899 USA

## Abstract

We introduce a simple criterion to identify two-dimensional (2D) materials based on the comparison between experimental lattice constants and lattice constants mainly obtained from Materials-Project (MP) density functional theory (DFT) calculation repository. Specifically, if the relative difference between the two lattice constants for a specific material is greater than or equal to 5%, we predict them to be good candidates for 2D materials. We have predicted at least 1356 such 2D materials. For all the systems satisfying our criterion, we manually create single layer systems and calculate their energetics, structural, electronic, and elastic properties for both the bulk and the single layer cases. Currently the database consists of 1012 bulk and 430 single layer materials, of which 371 systems are common to bulk and single layer. The rest of calculations are underway. To validate our criterion, we calculated the exfoliation energy of the suggested layered materials, and we found that in 88.9% of the cases the currently accepted criterion for exfoliation was satisfied. Also, using molybdenum telluride as a test case, we performed X-ray diffraction and Raman scattering experiments to benchmark our calculations and understand their applicability and limitations. The data is publicly available at the website http://www.ctcms.nist.gov/~knc6/JVASP.html.

## Introduction

Two-dimensional (2D) materials^1, 2^ have great potential in sub-micron level electronics^3^, flexible and tunable electronics^4^, superconductivity^5^, photo-voltaic^6^, water purification^7^, sensors^8^, thermal management^9^, ethanol distillation and energy storage^10^, medicine^11^, quantum dots^12, 13^ and composites^14–16^. Despite a huge upsurge in graphene and other 2D materials research, search for novel 2D materials and their systematic comparison of properties is still in development phase^14, 17, 18^. A consistent, high throughput investigation of such materials using materials modelling tool such as density functional theory^19–21^ would allow better understanding of the nature and properties of 2D materials^22^.

One of the successful works in the field of making 2D material repository was initiated by Ding *et al*.^23^, who focused on investigating MX_2_ (M = Mo, Nb, W, Ta; X = S, Se, Te) monolayers for structural, vibronic and electronic properties using local density approximation (LDA) and semi-local generalized gradient approximation (GGA) with Perdew-Burke-Ernzerhof (PBE) functionals^24^. They also calculated hybrid functional (HSE) and many-body method based GW bandgaps of the materials^25^. However, one of the major limitations of this work is that they only considered honeycomb H structures for layered materials, while other structures have also been identified experimentally^26^. Another important database for 2D materials was developed by Bjorkman *et al*.^27^. They used DFT to characterize 2D materials with interlayer binding energies in the range of 10–20 meV/Å^2^, except those with significant covalent bonds. Their work shows that PBE strongly under-binds these materials, which can lead up to 20% overestimation of interlayer distance. Moreover, LDA is clearly shown to over bind materials. While authors’ focus is to study the structural properties of 2D materials with different functionals, they investigate bulk 2D materials only, instead of finite-number-of-layer cases, which are of much more technological importance. Later on, Lesbegue *et al*.^28^ used crystal packing fraction to identify bulk 2D materials from Inorganic Crystal Structure Database (ICSD) database^29^. They computed magnetic properties and PBE-based band structures for 46 candidate 2D materials. Although they provide criteria for selection of many 2D materials, they do not calculate properties for single/multi-layer materials. In addition, they performed all their calculations using the PBE functional, which has been found to be non-suitable for characterizing 2D materials and require more sophisticated vdW functionals than just using PBE functional. Ataca *et al*.^30^ also performed stability analysis to identify stable compounds among monolayer materials using an LDA functional, and they found 52 such materials. For all such compounds they also calculated elastic coefficients using LDA but only in the 2H and 1T hexagonal monolayer structures. Rasmussen *et al*.^22^ studied the electronic structures of 51 semiconducting monolayer transition-metal dichalcogenides and -oxides in the 2H and 1T hexagonal phases using DFT and taking into account many-body effects using GW approximation to alleviate DFT band-gap problem. Although this work gives a huge thrust towards 2D materials repository, it consists of prototype structures (2H and 1T) only, and their experimental fabrication is a debatable issue. In addition, it didn’t provide a robust method to identify 2D materials among all other bulk materials. Moreover, most of their computations are carried out with PBE for geometric optimization within DFT. Although, GW can be reinforced to obtain better band-gap predictions, important effects such as strain dependence of the bandgap and other properties are not reliable for 2D materials when computed with PBE. Very recently, Materialsweb repository has found hundreds of 2D materials using topological scaling algorithm^31^. Similar algorithm has been implemented by Mounet *et al*.^32^ to identify novel layered materials. DFT databases such as materials project (MP)^33^, Open Quantum Materials Database (OQMD)^34^, AFLOW^35^ have performed thousands of material data calculations with the PBE functional. These databases are quite homogenous among themselves and encompasses many of the ICSD materials data. While they provide a large amount of electronic structure, surface, interface and mechanical properties data, the choice of the PBE functional, fixed plane-wave energy cutoff and fixed K-point selection may not be well suitable for characterizing 2D materials^36^. Many 2D materials such as graphene require plane wave cut-off more than 600 eV^37^, but present DFT databases takes 520 eV cut-off for all materials uniformly. Additionally, these DFT databases do not contain any information for layered (such as single and bi-layer) material properties.

As evident from the above discussion, while there are many DFT-based databases available nowadays, there is still a significant need for a systematic evaluation of 2D material properties in monolayer, bilayer, and multi-layer forms, and for a comparison to their 3D bulk counterpart. Such properties should be calculated for all experimentally observed structures, not just conventional 2H and 1T, using DFT-functionals more suitable to the 2D case, such as optB88 functional^38, 39^. Also, sufficient convergence in plane-wave energy cut-off and K-point mesh is needed for reliable results. Lastly, a computationally-light criterion to identify whether a 3D material can be made into a 2D is still missing.

In this work, we developed such a criterion and then computed a vast array of properties for all the materials that our criterion identified as good 2D candidates. Specifically, our screening methodology is based on combining the finding from the Ataca *et al*.^30^ work and existing DFT databases. We notice above that PBE generally overestimates the lattice constant, especially along the direction of vdW interactions. We decided to use such a peculiarity as a basis for screening of materials. All major DFT databases use PBE and they also all contain the corresponding experimental lattice constants (from ICSD databases), for comparison purposes. So, we simply extract the PBE and the experimental lattice constants (*a, b* and *c*), and compute their relative error. The relative error in *a* or *b* or *c*-axis equal or greater than 5% is chosen as a preliminary screening criterion. After the preliminary identification, we repeat the DFT procedure for all these materials with better vdW functionals, such as optB88 to compute energetics and all other properties mentioned above.

For all possible 2D material candidates, our database provides materials properties for single layer as well as for their bulk counterparts. While the properties of many possible 2D materials have already been posted into our database, many of our calculations are still running, and their results will be automatically updated to the website once completed. All the possible 2D material candidates that we found are described in the results section below. The properties in our database include energetics, geometrical (computational X-ray diffraction) and electronic properties (band structure, density of states and work-function) and elastic constants for both the bulk and the single layer counterparts of materials. Most of the bulk materials have Inorganic Crystal Structure Database (ICSD) IDs^40^, so experimentalists can easily compare collected XRD patterns and look up how the actual materials are fabricated. Validation for all the 2D materials data with previously published work is beyond the scope of this paper, however we will discuss few examples of common 2D materials as benchmarking of the database. The data is distributed on National Institute of Standards and Technology (NIST) webpage for public use at http://www.ctcms.nist.gov/~knc6/JVASP.html. At present our database consists of 1012 bulk materials and 430 single-layer materials, and the database is still populating.

## Results

### Screening criterion and classification of vdW materials

As mentioned above, semi-local density functionals such as PBE are known to give incorrect results of lattice constants for vdW-bonded materials. All the publicly available DFT databases such as MP, AFLOW and OQMD uses PBE for property calculation. Hence, materials with significant error in lattice constants can be expected to be vdW bonded. We chose *δ* greater than or equal to 5% as screening criterion (for details see the discussion section) to identify possible 2D materials, where *δ* is the relative difference between ICSD (experimental data) and MP-PBE (DFT database computed using the PBE functional) lattice constants (*a*, *b* and *c* lattice constants chosen as in the ICSD and MP databases) for bulk materials in non-cubic crystal systems:

1 $$\delta =\frac{| {l}_{PBE}-{l}_{ICSD}| }{{l}_{ICSD}}\,{\rm{where}}\,l\in a,b,c$$

Using this screening criterion as an initial step, we identified 1356 possible 2D materials out of all the systems in the Materials Project database. Please note that we didn’t perform any of the DFT calculations needed to compute *δ*, we simply obtained the necessary quantities from the Materials Project database through REST API^41^.

**Figure 1:**
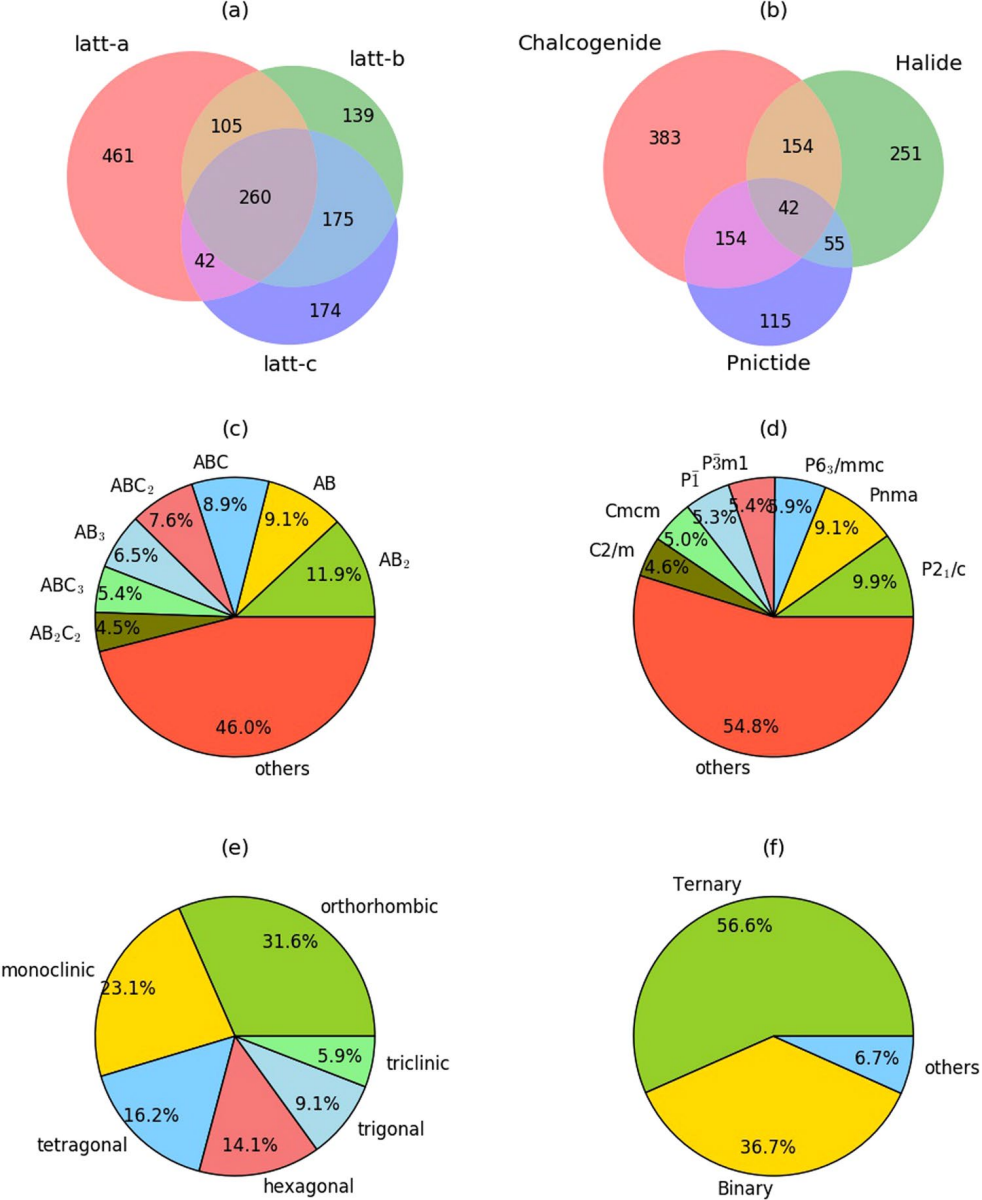
Classification of predicted layered materials in term of (**a**) relative error in lattice constants, (**b**) chemical compositions, (**c**) crystal prototypes, (**d**) crystal space group, (**e**) crystal-systems and (**f**) number of distinct chemical constituents.

An overall picture of the classification of these possible 2D materials is given in Fig. 1. To begin with, we found significant relative error in all lattice constants as well as their combinations (Fig. 1a). Specifically, the Venn diagram shows that the most of the materials have a relative error of 5% or more in lattice constant *a* (868 systems), followed by lattice constant *b* (679 systems) and, lastly, lattice constant *c* (651 systems). Relative errors equal or greater than 5% in all three lattice constants (*a*, *b*, and *c*) were found for 260 materials, while similar relative errors in only *a*, only *b*, and only *c* were found for 461, 139 and 174 materials, respectively. Lastly, relative errors equal or greater than 5% in both *a* as well as *b*, *a* as well as *c* and *b* as well as *c* were found for 105, 42 and 175 materials, respectively. We clearly observe that the Vander Waal bonding can have effect not just in *z* but also in *x* and *y* directions of the crystal.

With respect to the nature of these possible 2D materials, we found that most of the layered materials are chalcogenide in nature (Fig. 1b). However, halide, pnictides and their combinations are also possibilities. In Fig. 1c we show the structural prototype distribution of the materials in our database. While the major contribution is from AB_2_ structures, most of which are already known and have been fabricated^17^, other dominating stoichiometry are AB, ABC, ABC_2_ and AB_3_. These are less known potential 2D materials, possibly deserving further investigation. More details on the materials belonging to different structure-prototypes are given in the supplementary information (Table S1), where each prototype is listed with the corresponding materials. Like the prototype classification, in Fig. 1d we show the space group distribution of the predicted layered structures in our database. Space groups can change between bulk materials and corresponding single layer ones due to the broken periodicity in the Vander Wall direction. In the literature, $P\bar{6}m2$, $P\bar{3}m1$ and *P2*_*1*_ */m* structure spacegroups are commonly known as 2H, 1T and 1T’ single-layer structures. The space-groups for bulk structures of these layered materials are *P6*_*3*_ */mmc*, $P\bar{3}m1$ and *P2*_*1*_ */m*. 2H and 1T are the primary structures traditionally investigated in 2D computational materials repository databases^22^. However, Fig. 1d shows that 2D materials can have counterintuitive crystal structure space groups rather than just 2H and 1T structures. Recently, quite a few 2D materials have been experimentally fabricated which have crystal structures beyond 2H and 1T, such semi-metal WTe_2_ (space group Pmn2_1_)^42, 43^. In single layer materials space groups have direct implication on their properties. For instance, crystals with an inversion center cannot display piezoelectric behavior, and hence our space group results could be used to screen for 2D piezoelectric materials. Such a screening criterion has been already used by Materials Project to identify potential bulk piezoelectric materials^44^. Additionally, crystal structure space group information can be used in Raman experiments and second-harmonic generation (SHG) experiments, in which the spectral peaks are sensitive to space groups^45^. Interestingly, materials with lack of inversion symmetry can lead to valley-selective excitation of charge carriers^46, 47^. For this purpose, a detailed list of the materials in various space-group is given in supplementary information (Table S2). We also provide the bulk and layered space group information in our database so that it can be used in characterizing and identifying the materials experimentally.

In Fig. 1e we show the crystal system distribution of the bulk layered materials. Most of the layered materials belong to orthorhombic crystals system. Other major crystal systems are monoclinic and tetragonal systems. Up to this work bulk layered materials were mostly considered as having honey-comb structures in hexagonal system. However, our results clearly show that layered materials often belong to other crystal systems as well. A complete list of predicted layered materials in different crystal-systems are given in supplementary information (Table S3). In Fig. 1f, we show that most of our predicted layered materials are binary and ternary compounds. Our results are similar to the work by Ashton *et al*.^31^. As binary and ternary materials are generally easer to fabricate experimentally than others, the ICSD database is biased towards such compounds. Specifically, it contains 2,033 elementals, 34,785 binaries, 68,730 ternaries, 68,083 among quaternaries and quintenary compounds, as of year 2016–17. As our database imports structures from the Materials Project, and the Materials Project was built on the ICSD database, the same bias towards binary and ternary compounds can be observed.

As the choice of a 5% relative-error for our screening criterion is somewhat arbitrary, we recomputed all the quantities discussed in Fig. 1 using cutoff values of 3%, 7% and 10%. The complete results of these calculations are shown in Fig. S1 and in Tables S4–S5 in the supplementary information. As expected, the total number of screened compounds decreases as δ increases (3116, 1356, 819 and 375, for δ equal to 3%, 5%, 7% and 10%, respectively). However, in all cases the largest number of materials satisfying the screening criterion has large relative error in lattice constant *a* (Table S4). Similarly, the number of compounds with large relative error in lattice constant *b* is in all cases very close to the number of compounds with large relative error in lattice constant *c* (Table S4). We find similar behavior for chemical composition and stoichiometry. For all δ, the most prevalent chemical composition is chalcogenides, followed by halides and pnictides (Table S5). AB_2_ is always the most common crystal prototype, and AB, ABC are the second and third most likely crystal prototypes in all cases except for δ  = 3% (Fig. S1). It is worth pointing out that the probability of the selected material to be in the AB_2_ form grows monotonically as δ increases. The distribution of space groups shows less systematic behavior among all the investigated quantities. Orthorhombic crystal system is always the most commonly found structure, and monoclinic is the second most likely in all cases except for δ  = 10%, where it appears as the third most likely one.

**Figure 2:**
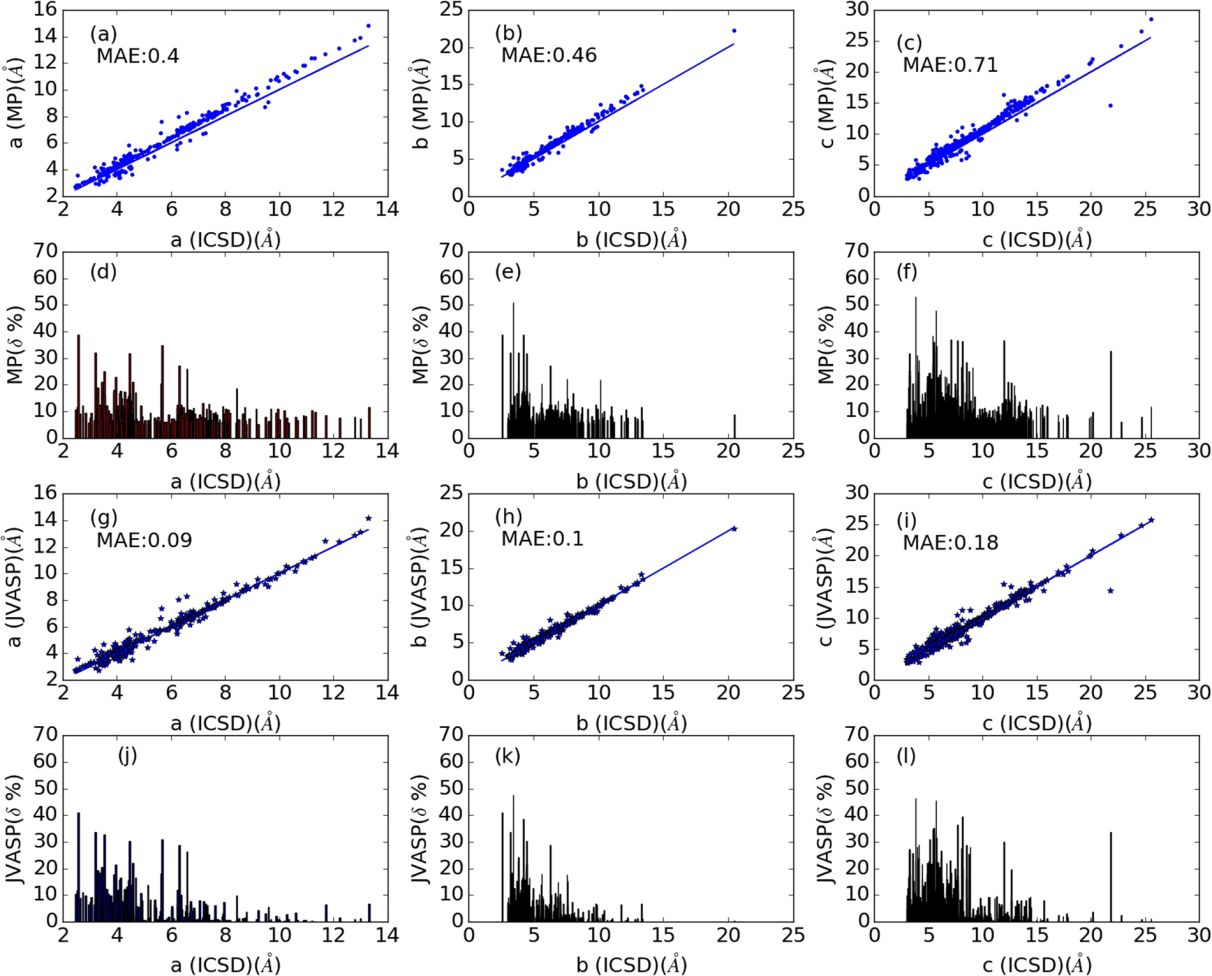
Comparison of lattice constants from Materials-project PBE calculations (**a**–**f**) and our optB88 functional calculations (**g**–**l**) with experimental ICSD data. Lattice constant a is examined in (**a**) PBE data and (**g**) optB88 data, while lattice constants b and c are given for PBE data in (**b**) and (**c**), respectively, and in (**h**) and (**i**) for optB88 data, respectively. In all cases the corresponding mean absolute error (using Eq. 2) is also reported, quantifying the improvement in lattice constants using optB88 over PBE. The data is plotted using dots, while the solid line is given just to indicate what the results should be for a perfect agreement between experimental and computational data. Similarly, relative percent errors with respect to the ICSD data are given in (**d**–**f**) for PBE *a*, *b*, and *c* lattice constants, respectively, and in (**j**–**l**) for optB88 equivalent data.

Conventionally, the accepted error in lattice constants computed using DFT with the PBE functional is about 2%^36, 48^ with respect to experimental values. As we found a lot of compounds with relative error in lattice constants greater than 5% (Fig. 1a), we noticed that PBE functional may not be the ideal functional to investigate 2D materials, and tested if the optB88 exchange-correlation functional^38, 39^ could be a better choice. We therefore relaxed the 1356 materials that our screening criterion had identified using the optB88 functional. Our results comparing lattice constants from ICSD to those obtained using PBE (Materials-Project data) and our optB88 functional-based data are shown in Fig. 2.

ICSD-data are given along the *x*-axis, and computational data are given along the *y*-axis (Fig. 2a–c and j–l). The DFT data is plotted using dots, while the solid lines indicate the results for a perfect agreement between experimental and computational DFT data. We find some degree of deviation from ideality in all cases, but such deviation is clearly more significant for PBE data than optB88. To better quantify such a deviation, we calculated the mean absolute error (MAE) in the data using equation 2:

2 $$MAE=\frac{1}{n}\sum _{i=1}^{n}| {x}_{i}-{y}_{i}| $$

where *x*_*i*_ is the optB88-based or the PBE-based DFT data and *y*_i_ is the corresponding ICSD data. Comparing MAE values, we find that optB88-MAE are always lower than PBE-MAE, up to 4 times lower in the case of lattice constant a (Fig. 2(a) and (g)), validating the assumption that the optB88 functional describes Vander Waal bonding better than PBE. The error in individual data points is also visualized in Fig. 2(d–f) and (j–l) as relative percentage error. We find that the relative error can be up as large as 53%, as it is the case for FeS (P4/nmm) computed using PBE (materials-project-id mp-20311). Examples of other materials with high relative error in lattice constants are SnS (Cmcm, mp-1379), RbMnAs (P4/nmm, mp-20242), KMnP (P4/nmm, mp-20422), NiBi (P6_3_/mmc, mp-22861), and SnCl2 (P2_1_/c, mp-29179). Although, optB88 can reduce the error in lattice-constants for the predicted layered materials, there still is plenty of room for improvement, as neither of these two functionals predicts lattice constants with 100% accuracy for all the materials. After identification of possible 2D materials with the lattice-constant criterion, we validate our findings by computing their exfoliation energy. The exfoliation energy for 2D materials is computed as equation 3:

3 $${E}_{f}=\frac{{E}_{1L}}{{N}_{1L}}-\frac{{E}_{bulk}}{{N}_{bulk}}$$

Here, *E*_*1L*_ and *E*_*bulk*_ are the energies of the single layer and 3D bulk materials and *N*_*1L*_ and *N*_*bulk*_ are the number of the atoms in the single layer and bulk systems respectively. Although PBE results are available for the 3D bulk structures in the Materials Project database, we carried out new bulk calculations with the optB88 functional to determine the exfoliation energy, for consistency between bulk and single layer energetics treatment. Both bulk and single layer data and metadata are available publicly through an easy-to-use web interface, which is discussed in the next section.

The conventional computational criterion^49–52^ to predict feasibility of exfoliation suggests that exfoliation energy for a feasible 2D materials should be less than 200 meV/atom. As for equation (2), both bulk and monolayer energies must be computed, and at present, we have carried out 1012 out of 1356 bulk calculations, and 430 single layer calculations. All the remaining calculations are currently in progress and the database is continuously expanding. It is to be noted that the computational cost of single layer material calculations can be much higher than bulk due to inclusion of vacuum padding in the simulation box. We have 371 materials in our database which have both single and bulk calculations completed, and out of these 330 (88.9%) have satisfied the exfoliation energy criterion (exfoliation energy below 200 meV) as shown in Fig. 1.

**Figure 3:**
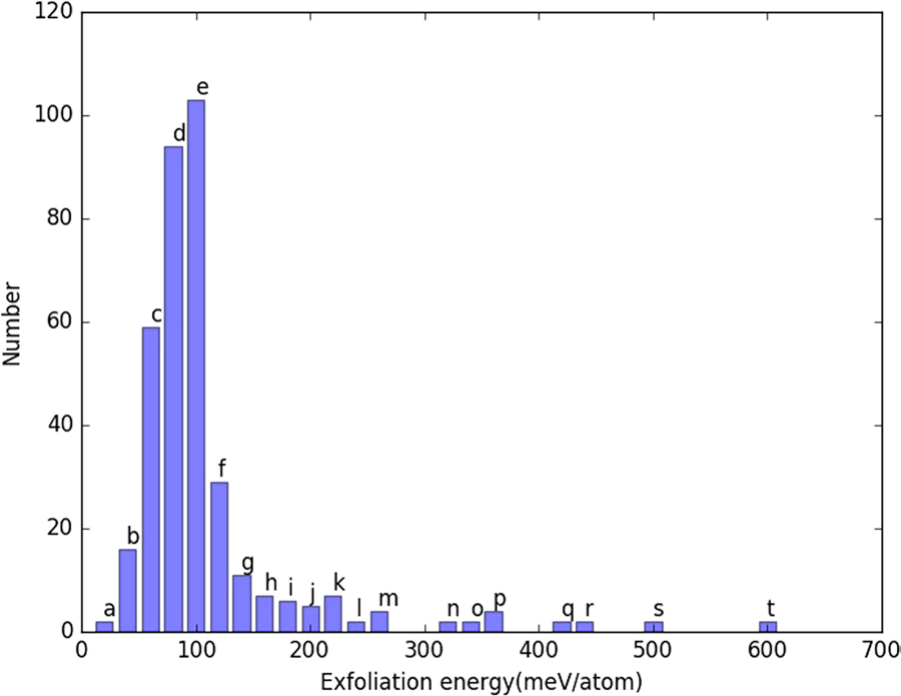
Exfoliation energy distribution for the materials based on the energy difference of bulk and layered materials phase. Most of the materials have E_f_ < 200 meV/atom suggesting experimental fabrication of layered structures. Materials inside each bins are grouped and discussed below. Most of the materials are found in the 60–100 meV range. Details of structures in each bins are given in Table 1.

Even more interestingly, most of the predicted layered structures were found to have exfoliation energies in the 60–100 meV range. This result clearly suggests that our simple criterion to distinguish vdW bonded structures based on the relative difference *δ* in lattice constants is a reasonable criterion, at least for the initial screening. As an added advantage, this criterion is very easy to apply and anybody can use it. To facilitate experimentalists requiring options for easy-to-fabricate 2D materials, we have grouped materials with respect to their energy of exfoliation in our database as shown in Fig. 3 and Table 1. Specifically, we have a group for exfoliation energies below 0–20 meV/atom, one for energies between 20 and 40 meV/atom, and so on. Along with the chemical composition, we provide the space group information for bulk and single-layer material in parenthesis, which are hyperlinked to our database webpage. Clicking on these space group navigate to database webpage directly. While there has not been experimental work on all of these 2D materials yet, many of them have been very recently predicted using DFT by Materialsweb database^31^ also. Materials such as TiIN, Sc_2_NCl_2_, OsCl_2_O, MnCl_2_, MgBr_2_ are found to be common between ours and Materialsweb database. A detailed comparison of these two databases needs to be made for a comprehensive outlook for 2D materials. Our criterion, however, suggests a few more (such as BCl_3_, Te_2_Br, AlI_3_, TiI_3_ and so on) materials that have not been identified yet. Moreover, new emerging 2D materials like BiTeI have already been proven candidate of 2D materials with very interesting physical phenomenon such as giant Rashba effect^53, 54^, hence other novel 2D materials could also be subjected to experimental fabrication. Although, it is not recommended to invest lot of effort in experimental fabrication of layered materials with *E*_*f*_ greater than 200 meV/atom, it is to be noted that large formation energy generally indicates that the 2D material could be formed only if a suitable substrate is found as for silicene and GeO^49, 55^ in which the exfoliation energy can take the value up to 600 meV/atom. Therefore, a layered material with high exfoliation energy cannot be completely discarded as candidate 2D materials.

**Table 1:** Classification of the database based on energy of exfoliation (meV).

Exfoliation-energy-bins (eV)	#	Materials
0–20	2	DyBC(Cmmm, P2/m)
20–40	16	Si_3_H(P $\bar{3}$ m1, P $\bar{3}$ m1), SbTe(P $\bar{3}$ m1, P $\bar{3}$ m1), ZrNCl(R $\bar{3}$ m, P $\bar{3}$ m1), HfNCl(R $\bar{3}$ m, P $\bar{3}$ m1), Mg(AlSe_2_)_2_(R $\bar{3}$ m, P $\bar{3}$ m1), LuHCl(R $\bar{3}$ m, P $\bar{3}$ m1), AlClO(Pmmn, Pmmn), Fe_3_S_4_(R3m, P3m1), ZrBrN(R $\bar{3}$ m, P $\bar{3}$ m1), YBrO(R $\bar{3}$ m, P $\bar{3}$ m1), HfBrN(R $\bar{3}$ m, P $\bar{3}$ m1), HoBrO(R $\bar{3}$ m, P $\bar{3}$ m1), TiNCl(Pmmn, Pmmn), SiH_4_(P2_1_ /c, Pc), ScBrO(Pmmn, Pmmn)
40–60	59	InClO(Pmmn, Pmmn), HfIN(R $\bar{3}$ m, P $\bar{3}$ m1), ErClO(Pmmn, Pmmn), Sc_2_CCl_2_(P $\bar{3}$ m1, P $\bar{3}$ m1), Sc_2_NCl_2_(P $\bar{3}$ m1, P $\bar{3}$ m1), TiBrN(Pmmn, Pmmn), CrBrO(Pmmn, Pmmn), TlSbO_3_(R $\bar{3}$, P $\bar{3}$ 1m), Bi_2_TeI(P $\bar{1}$, P $\bar{3}$ m1), TiIN(Pmmn, Pmmn), CeSiI(P $\bar{3}$ m1, P $\bar{3}$ m1), SiH(P $\bar{3}$ m1, P $\bar{3}$ m1), KMnP(P4/nmm, P4/nmm), CaHI(P4/nmm, P4/nmm), Sr(BiO_2_)_2_(C2/m, P2_1_ /m), TeO_3_(P6_3_ /mmc, P $\bar{6}$ m2), CoO_2_(P6_3_ /mmc, P6/mmm), Ta_2_CS_2_(R $\bar{3}$ m, P $\bar{3}$ m1), AlPd_5_I_2_(I4/mmm, P4/mmm), CaHBr(P4/nmm, P4/nmm), RbMnAs(P4/nmm, P4/nmm), RbMnP(P4/nmm, P4/nmm), NdIO(P4/nmm, P4/nmm), PrIO(P4/nmm, P4/nmm), Nb_2_CS_2_(R $\bar{3}$ m, P $\bar{3}$ m1), Nb_2_CS_2_(P $\bar{3}$ m1, P $\bar{3}$ m1), ScCl(R $\bar{3}$ m, P $\bar{3}$ m1), BiIO(P4/nmm, P4/nmm), ZrCl(C2/m, P $\bar{3}$ m1), Ge(BiTe_2_)_2_(R $\bar{3}$ m, P $\bar{3}$ m1), ThIN(P4/nmm, P4/nmm), MnO_2_(P $\bar{3}$ m1, P $\bar{3}$ m1), As_2_O_3_(P2_1_ /c, Pc), TiS_3_(P2_1_ /m, P2_1_ /m), CrS_2_(C2/m, C2/m), TbCl(R $\bar{3}$ m, P $\bar{3}$ m1), ZrS_3_(P2_1_ /m, P2_1_ /m), PbIF(P4/nmm, P4/nmm), DySI(Pmmn, Pmmn), HfS_3_(P2_1_ /m, P2_1_ /m), GaS(P6_3_ /mmc, P $\bar{6}$ m2), BiBrO(P4/nmm, P4/nmm), GaS(R $\bar{3}$ m, P $\bar{3}$ m1), SmTe_3_(Cmcm, P4/nmm), PrTe_3_(Cmcm, P4/nmm), NdTe_3_(Cmcm, P4/nmm), SrHI(P4/nmm, P4/nmm), YTe_3_(Cmcm, P4/nmm), NiO_2_(P $\bar{3}$ m1, P $\bar{3}$ m1), ClF(P2_1_ /c, P2_1_), US_3_(P2_1_ /m, P2_1_ /m), ZrSe_3_(P2_1_ /m, P2_1_ /m), HfSe_3_(P2_1_ /m, P2_1_ /m), GaSe(P6_3_ /mmc, P $\bar{6}$ m2), TbBr(R $\bar{3}$ m, P $\bar{3}$ m1), VO_2_(P1, P $\bar{3}$ m1), LuSBr(Pmmn, Pmmn), GaSe(R3m, P $\bar{6}$ m2), TiO_2_(R $\bar{3}$ m, P $\bar{3}$ m1)
60–80	94	Ta_2_Se(P4/nmm, P4/nmm), Al_3_Te_3_I(Pnma, P2_1_ /m), DySBr(Pmmn, Pmmn), SbCl_5_(P6_3_ /mmc, P $\bar{1}$), ErSeI(Pmmn, Pmmn), AgClO_2_(Pcca, Pcca), MgCl_2_(R $\bar{3}$ m, P $\bar{3}$ m1), HfSiTe(P4/nmm, P4/nmm), TiS_2_(R $\bar{3}$ m, P $\bar{3}$ m1), ErSCl(Pmmn, Pmmn), Ni(HO)_2_(P $\bar{3}$ m1, P $\bar{3}$ m1), CrAg(PS_3_)_2_(P2/c, P2/c), AuClO_2_(C222_1_, P222_1_), GaTe(P6_3_ /mmc, P $\bar{6}$ m2), Zr_2_Te_2_P(R $\bar{3}$ m, P $\bar{3}$ m1), TaI_2_O(C2/m, P2/m), CoO_2_(P $\bar{3}$ m1, P $\bar{3}$ $\bar{3}$ m1), ScPS_4_(P $\bar{1}$, P $\bar{1}$), AlCl_3_(C2/m, C2/m), TiCl_2_(P $\bar{3}$ m1, P $\bar{3}$ m1), Bi_2_Se_3_(R $\bar{3}$ m, P $\bar{3}$ m1), CdPS_3_(C2/m, C2/m), FeCl_2_(R $\bar{3}$ m, P $\bar{3}$ m1), NbO_2_(P6_3_ /mmc, P $\bar{6}$ m2), CoCl_2_(R $\bar{3}$ m, P $\bar{3}$ m1), MnCl_2_(R $\bar{3}$ m, P $\bar{3}$ m1), Bi_2_Te_2_S(R $\bar{3}$ m, P $\bar{3}$ m1), InSe(R3m, P3m1), AlHO_2_(P2_1_ /m, P2_1_ /m), Nb(SCl)_2_(C2/m, C2/m), USe_3_(P2_1_ /m, P2_1_ /m), CdPS_3_(R $\bar{3}$, P $\bar{3}$), ScCl_3_(R $\bar{3}$, P $\bar{3}$), ZrCl_2_(R3m, P $\bar{6}$ m2), Nb(SeCl)_2_(P $\bar{1}$, P $\bar{1}$), CrCl_3_(C2/m, C2/m), ScAg(PS_3_)_2_(P $\bar{3}$ 1c, P312), SiP_2_(Pbam, Pmc2_1_), C(P6_3_ /mmc, P6/mmm), Bi_2_Te_2_Se(R $\bar{3}$ m, P $\bar{3}$ m1), InAg(PS_3_)_2_(P $\bar{3}$ 1c, P312), HgPS_3_(P $\bar{1}$, P $\bar{1}$), CdCl_2_(R $\bar{3}$ m, P $\bar{3}$ m1), BiClO(P4/nmm, P4/nmm), BN(P6_3_ /mmc, P $\bar{6}$ m2), HfCl_4_(P2/c, P2/c), Te_3_As_2_(R $\bar{3}$ m, P $\bar{3}$ m1), Sb_2_Te_2_Se(R $\bar{3}$ m, P $\bar{3}$ m1), HfFeCl_6_(P $\bar{3}$ 1c, P312), RhCl_3_(C2/m, C2/m), MgPSe_3_(R $\bar{3}$, P $\bar{3}$), MgBr_2_(P $\bar{3}$ m1, P $\bar{3}$ m1), ZrFeCl_6_(P $\bar{3}$ 1c, P312), ThBrN(P4/nmm, P4/nmm), Sb_2_TeSe_2_(R $\bar{3}$ m, P $\bar{3}$ m1), FeCl_3_(R $\bar{3}$, P $\bar{3}$ 1m), TiCl_3_(P $\bar{3}$ 1m, P $\bar{3}$ 1m), ZrGeTe(P4/nmm, P4/nmm), Nb_3_Cl_8_(P $\bar{3}$ m1, P3m1), Nb_3_TeCl_7_(P $\bar{3}$ m1, P3m1), VCl_3_(R $\bar{3}$, P $\bar{3}$ 1m), IrCl_3_(C2/m, C2/m), OsCl_2_O(Immm, Pmmm), NbCl_4_(C2/m, C2/m), MnBr_2_(P $\bar{3}$ m1, P $\bar{3}$ m1), FeBr_2_(P $\bar{3}$ m1, P $\bar{3}$ m1), TcS_2_(P $\bar{1}$, P $\bar{1}$), GaAg(PSe_3_)_2_(P $\bar{3}$ 1c, P312), ReSe_2_(P $\bar{1}$, P $\bar{1}$), ScAg(PSe_3_)_2_(P $\bar{3}$ 1c, P312), CoBr_2_(P $\bar{3}$ m1, P $\bar{3}$ m1), CS_2_(Cmce, C2/m), CrSe(P4/nmm, P4/nmm), TiBr_2_(P $\bar{3}$ m1, P $\bar{3}$ m1), CdBr_2_(P6_3_ mc, P3m1), WS_2_(P6_3_ /mmc, P $\bar{6}$ m2), SnO_2_(Cm, P1), VBr_2_(P $\bar{3}$ m1, P $\bar{3}$ m1), MoS_2_(P6_3_ /mmc, P $\bar{6}$ m2), YBr_3_(C2/m, C2/m), TmAg(PSe_3_)_2_(P $\bar{3}$ 1c, P312), BiI(C2/m, C2/m), ErAg(PSe_3_)_2_(P $\bar{3}$ 1c, P312), AsCl_3_(P2_1_ 2_1_ 2_1_, P2_1_), ZnCl_2_(P4_2_ /nmc, P $\bar{4}$ m2), SNCl(P2_1_ /m, Pm), Sb_2_Te_3_(R $\bar{3}$ m, P $\bar{3}$ m1), Hf_3_Te_2_(I4/mmm, P4/mmm), ZrI_2_(P2_1_ /m, P2_1_ /m), InAg(PSe_3_)_2_(P $\bar{3}$ 1c, P312), CrBr_3_(R $\bar{3}$, P $\bar{3}$ 1m), WSe_2_(P6_3_ /mmc, P $\bar{6}$ m2), PtO_2_(P6_3_ mc, P $\bar{3}$ m1), PPdS(Pbcn, P2/c)
80–100	103	ThI_2_(P6_3_ /mmc, P $\bar{3}$ m1), MoSe_2_(P6_3_ /mmc, P $\bar{6}$ m2), GaTeCl(Pnnm, Pmn2_1_), RhBr_3_(C2/m, C2/m), BiBr_3_(P2_1_ /c, P2_1_ /c), NaN_3_(C2/m, P2/m), IrBr_3_(C2/m, C2/m), As_2_O_3_(P2_1_ /c, P2_1_), Al_2_Te_3_(P2_1_ /c, P2_1_ /c), MnSe(P4/nmm, P4/nmm), AlSiTe_3_(P $\bar{3}$ 1m, P $\bar{3}$ 1m), MgI_2_(P $\bar{3}$ m1, P $\bar{3}$ m1), MnI_2_(P $\bar{3}$ m1, P $\bar{3}$ m1), SnS_2_(P6_3_ mc, P $\bar{3}$ m1), TbCl_3_(Cmcm, Pmmn), BPS_4_(I222, P222), CaI_2_(P $\bar{3}$ m1, P $\bar{3}$ m1), S_5_N_6_(C2/c, C2), HgBr_2_(Cmc2_1_, Cm), SrHBr(P4/nmm, P4/nmm), CrI_2_(C2/m, C2/m), FeI_2_(P $\bar{3}$ m1, P $\bar{3}$ m1), PPdSe(Pbcn, P2/c), SnS_2_(P $\bar{3}$ m1, P $\bar{3}$ m1), VSe_2_(P $\bar{3}$ m1, P $\bar{3}$ m1), Ni_2_SbTe_2_(P6_3_ /mmc, P $\bar{6}$ m2), AlPS_4_(P222, P222), CdI_2_(P6_3_ mc, P $\bar{3}$ m1), PtO_2_(P $\bar{3}$ m1, P $\bar{3}$ m1), AuBr(P4_2_ /ncm, Cmme), Te_2_W(Pmn2_1_, P2_1_ /m), CaPbI_4_(P2/m, P2/m), FeS(P4/nmm, P4/nmm), CS_3_N_4_(P2_1_ /c, Pc), PbO(P4/nmm, P4/nmm), SiS_2_(Ibam, Pccm), P_4_S_5_(P2_1_ /m, Pm), GeI_2_(P $\bar{3}$ m1, P3m1), VS_2_(P $\bar{3}$ m1, P $\bar{3}$ m1), HfS_2_(P $\bar{3}$ m1, P $\bar{3}$ m1), Te_2_Mo(Pmn2_1_, P2_1_ /m), Te_2_Mo(P2_1_ /m, P2_1_ /m), Re(AgCl_3_)_2_(R $\bar{3}$, P $\bar{3}$), TeAuCl_7_(P $\bar{1}$, P1), CrSiTe_3_(R $\bar{3}$, P $\bar{3}$), BiOF(R $\bar{3}$ m, P $\bar{3}$ m1), TaS_2_(P6_3_ /mmc, P $\bar{6}$ m2), PbI_2_(R $\bar{3}$ m, P $\bar{3}$ m1), SmBr_3_(Cmcm, Pmmn), Nb_3_SBr_7_(P3m1, P3m1), TiO_2_(P6_3_ /mmc, P6/mmm), PdSCl(P2_1_ /m, Pm), TaSe_2_(R3m, P $\bar{6}$ m2), SbBr_3_(P2_1_ 2_1_ 2_1_, Pmn2_1_), AgI(P4/nmm, P4/nmm), AuI(P4_2_ /ncm, Cmme), ZrS_2_(P $\bar{3}$ m1, P $\bar{3}$ m1), PbI_2_(P $\bar{3}$ m1, P $\bar{3}$ m1), AsBr_3_(P2_1_ 2_1_ 2_1_, P2_1_), MoS_2_(R $\bar{3}$ m, C2/m), TiS_2_(P $\bar{3}$ m1, C2/m), TiS_2_(P $\bar{3}$ m1, P $\bar{3}$ m1), AlSeBr_7_(Pc, P1), FeTe(P4/nmm, P4/nmm), TaSe_2_(P6_3_ mc, P $\bar{6}$ m2), TmI_2_(P $\bar{3}$ m1, P $\bar{3}$ m1), Te_2_Mo(P6_3_ /mmc, P $\bar{6}$ m2), TaSe_2_(P6_3_ /mmc, P $\bar{6}$ m2), TaS_2_(P $\bar{3}$ m1, P $\bar{3}$ m1), HfSe_2_(P $\bar{3}$ m1, P $\bar{3}$ m1), PbClF(P4/nmm, P4/nmm), YI_3_(R $\bar{3}$, P $\bar{3}$), SiTe_2_(P $\bar{3}$ m1, P $\bar{3}$ m1), TiNbS_4_(C2/m, P2/m), NbS_2_(P6_3_ /mmc, P $\bar{6}$ m2), SnSe_2_(P $\bar{3}$ m1, P $\bar{3}$ m1), BeBr_2_(Ibam, Pccm), SbAsO_4_(P2_1_ /m, P2_1_ /m), As_4_S_3_(Pnma, P2_1_ /m), TiSe_2_(P $\bar{3}$ m1, P $\bar{3}$ m1), SN(P2_1_ /c, P $\bar{1}$), Ta_3_TeI_7_(P6_3_ mc, P3m1), MoBr_3_(Pmmn, Pmm2), ZrSe_2_(P $\bar{3}$ m1, P $\bar{3}$ m1), PtS_2_(P $\bar{3}$ m1, P $\bar{3}$ m1), AlTeI_7_(Pc, P1), TiSe(P4/nmm, P4/nmm), ZrTiSe_4_(P2/m, P2/m), PCl_3_(Pnma, Pmc2_1_), BiI_3_(R $\bar{3}$, P $\bar{3}$), PI_2_(P $\bar{1}$, P $\bar{1}$), AsI_3_(R $\bar{3}$, P $\bar{3}$), SN(P2_1_ /c, P2_1_), TaSe_2_(P $\bar{3}$ m1, P $\bar{3}$ m1), NbS_2_(P $\bar{3}$ m1, P $\bar{3}$ m1), PSe(P2_1_ /c, P2_1_), Nb_3_TeI_7_(P6_3_ mc, P3m1), SbI_3_(R $\bar{3}$, P $\bar{3}$), NbSe_2_(P6_3_ /mmc, P $\bar{6}$ m2), NdI_2_(I4/mmm, P4/mmm), RuBr_3_(P6_3_ /mcm, Pmma), CuSe_2_Br(P2_1_ /c, P2_1_), Ta_3_SeI_7_(P6_3_ mc, P3m1)
100–120	29	AlBr_3_(P2_1_ /c, P $\bar{1}$), TaTe_4_Ir(Pmn2_1_, P2_1_ /m), SnO(P4/nmm, P4/nmm), S_3_(NCl)_2_(P2_1_, P1), Te_2_Br(Pnma, Pmn2_1_), SbSI(P2_1_ 2_1_ 2_1_, Pmmn), PbF_4_(I4/mmm, P4/mmm), WO_2_(P6_3_ /mmc, P6/mmm), Pd(Se_3_Cl)_2_(P2_1_ /c, P $\bar{1}$), CaThBr_6_(Pmma, Pmm2), BiTeI(P3m1, P3m1), BiTeCl(P6_3_ mc, P3m1), PbBr_2_(Pnma, P2_1_ /m), TlPt_2_S_3_(P $\bar{3}$ m1, P $\bar{3}$ m1), CuSe_2_Cl(P2_1_ /c, P2_1_), Te_2_I(Pnma, Pmn2_1_), PtSe_2_(P $\bar{3}$ m1, P $\bar{3}$ m1), CrS_2_(P $\bar{3}$ m1, P $\bar{3}$ m1), Nb_2_Te_6_I(P2_1_ /c, P $\bar{1}$), HfTe_2_(P $\bar{3}$ m1, P $\bar{3}$ m1), BCl_3_(P6_3_ /m, P $\bar{6}$), CBrN(Pmmn, Pmm2), HgI_2_(P4_2_ /nmc, P $\bar{4}$ m2), PBr_3_(Pnma, Pmc2_1_), TiTe_2_(P $\bar{3}$ m1, P $\bar{3}$ m1), BiSBr(Pnma, P2_1_ /m), TiI_3_(Pmmn, Pmm2), P(Cmce, Pmna), SrThBr_6_(Pmma, Pmm2)
120–140	11	AlI_3_(P2_1_ /c, P $\bar{1}$), P_2_Se_5_(P2_1_ /c, P1), SbSBr(Pnma, P2_1_ /m), TlPd_2_Se_3_(P $\bar{3}$ m1, P $\bar{3}$ m1), Bi_2_Se_3_(Pnma, P2_1_ /m), BBr_3_(P6_3_ /m, P $\bar{6}$), CuTe_2_Br(P2_1_ /c, P2_1_), As_2_Se_3_(P2_1_ /c, Pc), As(Cmce, Pmna), TlTe_3_Pt_2_(P $\bar{3}$ m1, P $\bar{3}$ m1), Sb_2_S_3_(Pnma, P2_1_ /m)
140–160	7	Ta(ICl)_2_(Immm, Pmmm), SnS(Pnma, Pmn2_1_), SnSe(Cmcm, P4/nmm), Te_2_Pt(P $\bar{3}$ m1, P $\bar{3}$ m1), PdS_2_(Pbca, P2_1_ /c), SnSe(Pnma, Pmn2_1_), BI_3_(P6_3_ /m, P $\bar{6}$ 2m)
160–180	6	BiO_2_(P6_3_ /mmc, P $\bar{6}$ m2), AuSe(C2/m, P2/m), PdSe_2_(Pbca, P2_1_ /c), CaMnSi(P4/nmm, P4/nmm), NiTe_2_(P $\bar{3}$ m1, P $\bar{3}$ m1), NbI_5_(P2_1_ /c, Pc)
180–200	5	PI_3_(P6_3_, P3), PbS(Aem2, Aem2), BaBrCl(Pnma, P2_1_ /m), HgCl_2_(Pnma, Pmc2_1_), ZrS(P4/nmm, P4/nmm)
200–220	7	Te_2_Rh(P $\bar{3}$ m1, P $\bar{3}$ m1), SbSI(Pnma, P2_1_ /m), Te_2_Pd(P $\bar{3}$ m1, P $\bar{3}$ m1), Ba_2_N(R $\bar{3}$ m, P $\bar{3}$ m1), Te_2_Ir(P $\bar{3}$ m1, P $\bar{3}$ m1), SrO_2_(I4/mmm, P4/mmm), BiSeI(Pnma, P2_1_ /m)
220–240	2	BiSI(Pnma, P2_1_ /m), SbSeI(Pnma, Pmn2_1_)
240–260	4	InBi(P4/nmm, P4/nmm), Na_3_As(P6_3_ /mmc, P $\bar{6}$ m2), Bi_2_Te_3_(R $\bar{3}$ m, P3m1), Bi_2_Pt(P $\bar{3}$ m1, P $\bar{3}$ m1)
300–320	2	Mg_3_Au(P6_3_ /mmc, P $\bar{6}$ m2), Te_2_Au(C2/m, C2/m)
320–340	2	CaSn(Cmcm, Pmma), KAuSe(Cmcm, Pmma)
340–360	4	KAuS(Cmcm, Pmma), RbAuSe(Cmcm, Pmma), CaSi(Cmcm, Pmma), RbAuS(Cmcm, Pmma)
400–420	2	HgBr(I4/mmm, Pmmm), HgCl(I4/mmm, Pmmm)
420–440	2	Sc_2_C(P $\bar{3}$ m1, P $\bar{3}$ m1), CeS(I4/mmm, P4/nmm)
480–500	2	SiAs_3_(P6_3_ /mmc, P6/mmm), GaN(P6_3_ mc, P $\bar{6}$ m2)
580–600	2	SiSb_3_(P6_3_ /mmc, P6/mmm), Sb_3_Pb(P6_3_ /mmc, P $\bar{6}$ m2)

Out 1356 only 430 number of materials have both bulk and single layer calculation completed and the rest of the calculations are underway.

The table is updated online (website:﻿ www.ctcms.nist.gov/~knc6/JVASP.html) as further calculations get completed. Chemical compositions as well as their bulk and single layer space groups hyperlinked to our database are provided in parenthesis.

### Applicability and validation of the database

Our database provides the users with immediate access to several properties that are key in describing a 2D material and determining its technological applicability. All such properties have been computed consistently, to enable meaningful comparison between different materials. The properties that we focused on in this database are energetics, structural, elastic and electronic properties for both monolayer and bulk materials. While some of the calculated properties can deviate from experimental data, a consistent database like ours will enable at least a meaningful qualitative comparison of materials. We provide the applicability and limitation of our database as discussed below.

### X-ray diffraction pattern of bulk and monolayer materials

**Figure 4:**
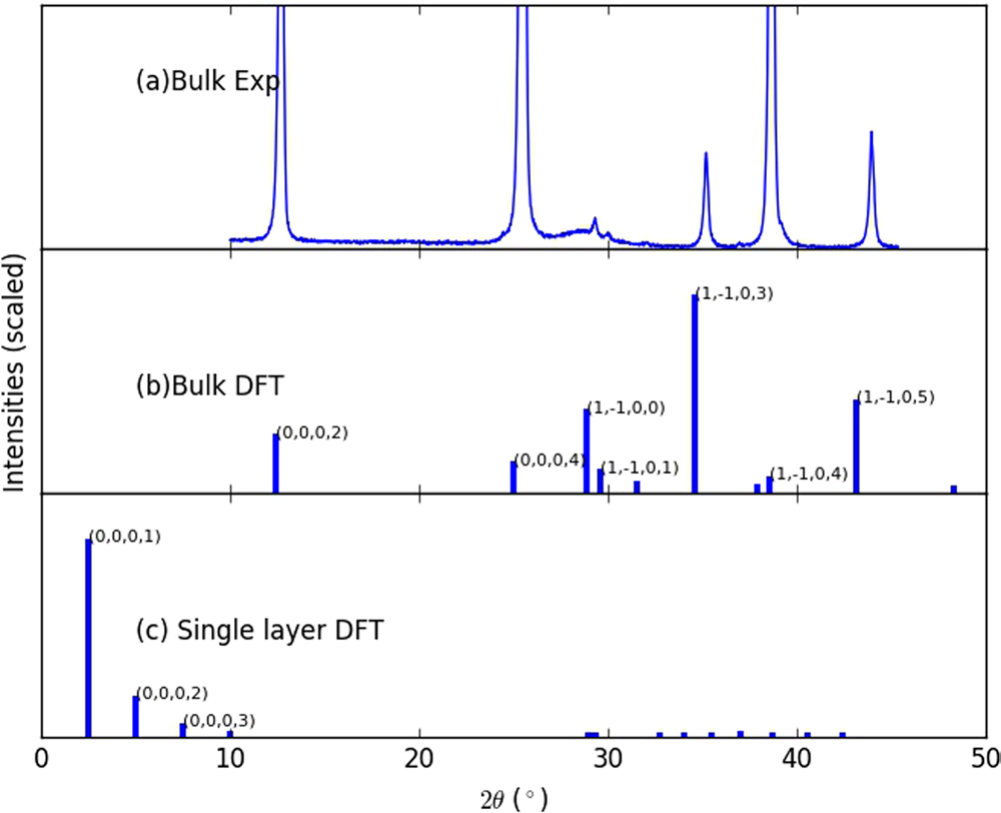
XRD plots for (**a**) bulk 2H-MoTe_2_ obtained with experiment, (**b**) bulk 2H-MoTe_2_ obtained with DFT using optB88 functional, (**c**) single layer MoTe_2_ with DFT using optB88 functional. Some of the important peaks are labelled with corresponding Miller indices. Single layer XRD have significant difference from the bulk material.

XRD patterns act as key signatures for materials. Using DFT optimized crystal structure, XRD patterns for bulk and monolayer are obtained in a consistent way. As an example, XRD patterns for bulk and single layer 2H-MoTe_2_ are shown in Fig. 4. To validate our DFT-based XRD, we also carried out experiments (Fig. 4a) to benchmark the bulk 2H-MoTe_2_ structure. As shown in Fig. 4b, there is great agreement between the DFT and the experimental XRD peak positions, while the intensities do not always match in the case of bulk 2H-MoTe_2_, which can be attributed to the experimental sample preparation techniques. For single layer MoTe_2_, the XRD pattern is shown in Fig. 4c. Peak positions are quite different between single layer and bulk, which could be explained in terms of the structure modifications occurring as we switch from bulk to single layer MoTe_2_. Strong diffraction lines for single layer materials are a characteristic feature of 2D materials, as they are also observed in the case of MoS_2_^56^. Although experimental XRD patterns are commonly available for bulk materials, obtaining experimental single layer XRD is much more challenging. This adds to the value of our database, as we are providing such a quantity and it could be used by experimentalist as a reference to compare and analyze their findings. Once again, it is important to point out that vdW functionals such as optB88, or conventional LDA functional produce XRD patterns for bulk materials that well match experimental ones, while semi-local functionals like PBE give erroneous XRD patterns, as they are incorrect in determining lattice constants to begin with. We examined at least 20 different material cases using three different exchange correlation functionals, to guarantee we used the most appropriate one for our DFT calculations. Results clearly show that the peak positions in computational XRD using optB88 are very close to experimental ones, while their intensities may not match. A comparison of XRD experimental results to XRD DFT spectra obtained using 3 different exchange correlation functionals is given in the supplementary information (Fig. S2), together with 5 more examples of good match between optB88 spectra and experimental ones (Fig. S3).

### Band-structure of bulk and monolayer materials

**Figure 5:**
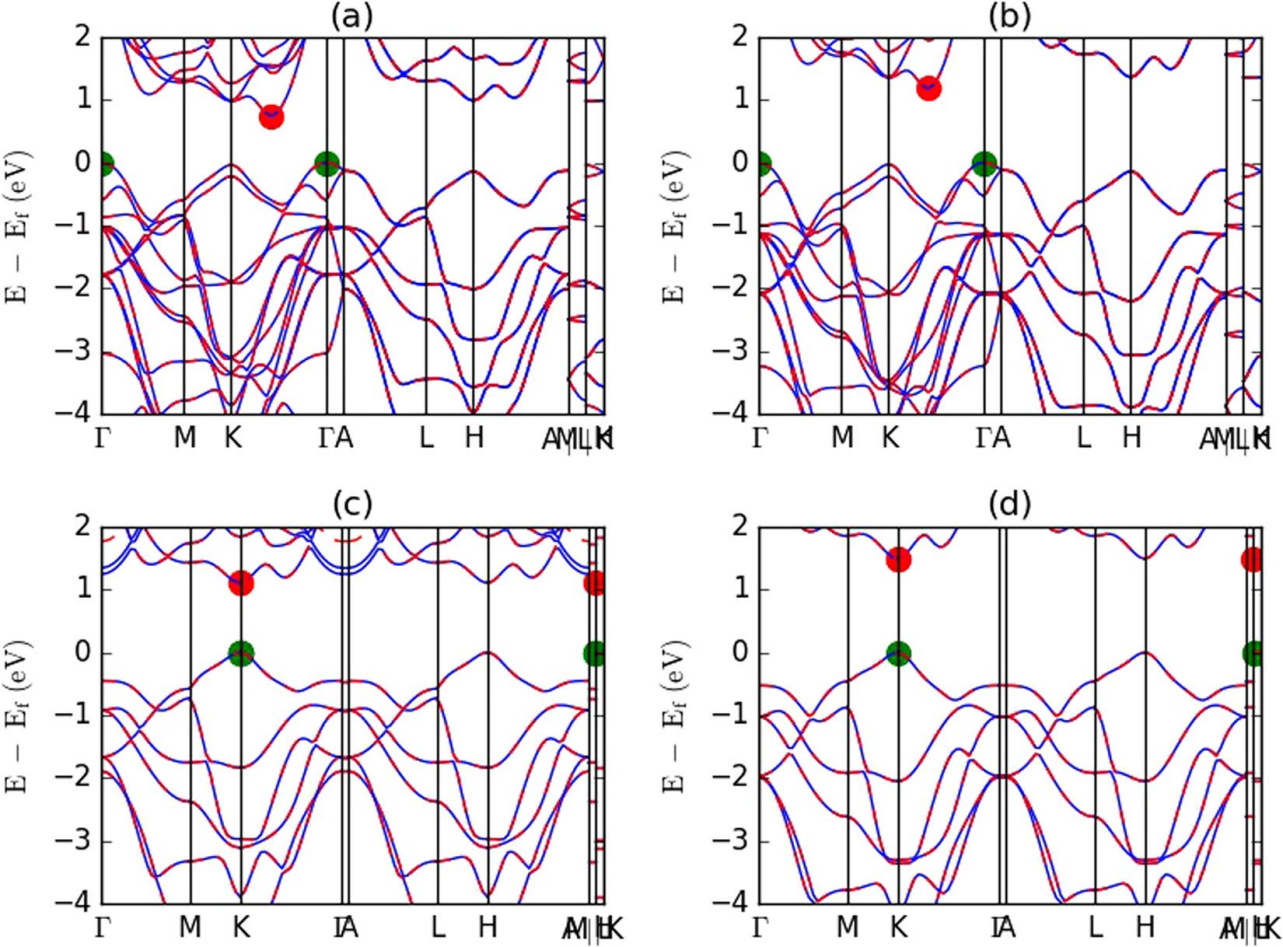
Bandstructures for MoTe_2_ in bulk and layered phases. (**a**) Shows bandstructure for 2H MoTe_2_ with optB88 and (**b**) with HSE06 functional. Similarly, (**c**,**d**) show bandstructure of single layer 2H MoTe_2_ with optB88 and HSE06 functionals, respectively. Change of nature of bandgap from indirect to direct for bulk and single layer phases is validated.

We compute band structures, density of states and plane-average electrostatic potential to characterize the electronic properties of bulk and single layer materials. An example for 2H bulk and single layer MoTe_2_ is shown in Fig. 5. As expected, we notice a change in the nature of the bandgap, from indirect to direct, and a change in the bandgap value going from bulk to single layer. In the figure the red dots indicate the conduction band minima and the green dots show the valence band maxima. In addition to results computed using the optB88 functional, our database also contains HSE06 functional-based band structures for few materials. HSE06 functionals are important to obtain correct description of bandgaps of materials because all the local and semilocal DFT such as LDA and GGA are prone to underestimate the bandgaps. However, HSE06 are computationally very expensive, hence only few HSE06 for bulk and single layer materials are calculated and the database for HSE06 calculations is still populating. Nevertheless, in absence of better results, our optB88 based bandgaps could still be used to predict qualitative trends. To validate our high throughput electronic property data for single-layer and bulk materials, we compared our results to previous experimental and DFT data for quite a few 2D materials, as shown in Table 2. We notice that, although the trends in the bandgaps look consistent, their exact values are less satisfactory. We also observe that optB88 generally underestimates, and HSE06 somewhat overestimates the bandgaps.

**Table 2:** Table shows crystallographic, electronic and elastic properties of some commonly known 2D materials for comparison and validation of the database.

Mat	SG	Bg (eV)	BgH (eV)	Bgr (eV)	c_11_	c_11r_	Φ (eV)	Φ_r_ (eV)
MoS_2_	P63/mmc	0.919	1.493	1.23^a^	214.6	238^d^	−5.32	—
L-MoS_2_	P-6m2	1.714	2.142	2.48^f^	134.1	138.1^c^	−5.43	−5.07^e^
WSe_2_	P63/mmc	1.002	1.516	1.2^a^	183.7	—	−4.72	—
L-WSe_2_	P-6m2	1.546	—	2.08^f^	121.5	130.04^c^	−4.83	−4.21^e^
MoSe_2_	P63/mmc	0.873	1.404	1.09^a^	169.7	—	−4.77	—
L-MoSe_2_	P-6m2	1.481	1.891	2.18^f^	111.8	118.37^c^	−4.96	−4.57^e^
MoTe_2_	P63/mmc	0.754	—	1.0^b^	109.5	—	−4.56	—
L-MoTe_2_	P-6m2	1.107	1.484	1.72^f^	84.8	92.7^c^	−4.65	−4.29^e^
WS_2_	P63/mmc	1.024	1.6	1.35^a^	233.3	—	−5.29	—
L-WS_2_	P-6m2	1.606	—	2.43^f^	146.5	151.48^c^	−5.41	−4.73^e^

We compared our data with experiment/other DFT results whichever available. SG stands for space group, Bg is the band gap with optB88 functional, BgH is the bandgap with HSE06 functional, Bgr is the previously reported band-gap values from DFT/experiments. C11 is the elastic constant for bulk material in GPa unit and elastic coefficient for layered material in N/m unit, φ is work-function obtained with optB88 and φ_r_ is corresponding previously reported values from literature. Ref. a, b and d are from previously reported experimental results while others are from previous DFT calculations. a^68^, b^69^, c^30^, d^61^, e^70^, f ^22^.

A detailed list of bandgaps using optB88 functional is given in the supplementary information, to enable an appropriate selection of materials (Table S6). DFT generally underestimates band-gaps of materials, hence if a material has a zero bandgap, it may or not be metallic compared to experimental data. Thankfully if DFT predicts non-zero bandgap then the material must be semiconducting or insulator in nature. We find 61% optB88-based bandgaps of single layer materials are greater than 0 or in other words they are non-metallic. This suggests that most of the layered materials in our database could be semiconducting or insulating in nature. HSE06 based bandgaps for most of the materials are underway, which can give better prediction of bandgaps. Interestingly, the metallic phase materials can also be of technological importance due to emergence of topological states, which are currently an area of intense research^57^. A detailed analysis of whether a material has a topological state or not is beyond the scope of present paper. Lastly, we also computed the work-function of the single-layer materials using the z-direction averaged potentials, as shown in Table 2. These values can be used to characterize the n/p nature of the 2D heterostructures. For a heterostructure made with two single layer materials, if the work-function for the first material is higher than the second one, then the first one should act as an n-type and second one as a p-type semiconductor. The plane average potential and hence work-functions are calculated for all the 2D materials. As evident from Table 2, our work function mass values have great agreement with previously reported value in the literature.

### Elastic properties of bulk and monolayer materials

Next, we calculate the elastic constants for bulk and single layer materials. Elastic constant calculations for layered materials are tricky^58^, because of their area nature instead of volume nature used in calculation. It is to be noted that during the calculation the vacuum size is taken as z-dimension of the simulation box so to make the elastic constant z-dimension free, we rescale the elastic constants with the z-simulation box size giving the elastic constant in the unit of N/m^58, 59^. Moreover, the bulk and the monolayer elastic properties are not comparable but all the monolayer data could be compared for their elastic properties among themselves. A table for comparison of elastic constant for some reported elastic constant in the literature in Table 2. The DFT data for elastic constants are found to be within 10% tolerance of the previously reported values. It correctly reflects our data are comparable to previously reported data. Similar to the structural data, elastic constants for 2D materials are sensitive to the selection of functional. A comparison of the elastic constants using different functionals are given in supplementary information (Table S7). We detect that elastic constants could deviate upto 27% with PBE functional are used such as for bulk MoS_2_ (176 GPa^60^ compared to 238 GPa from experiment^61^), however with optB88 (214 GPa) the error is generally within 10%.

Another key property that can help experimentalists characterize their data is the phonon description. These phonon modes act as the signatures of materials and can be easily compared with experimental data. One of the key advantage characterizing the 2D or any other materials with phonon is that the experimental spectra is less affected by impurities in the materials than X-ray photoelectron spectroscopy (XPS) and other experimental techniques. However, we can only compute Gamma-point phonons using the conventional cell for each material for computational cost reasons. Highly negative phonon modes generally imply structural instability or phase-transition in the material. It is to be emphasized that elastic constants and phonon modes generally require a careful investigation of finite size effects on properties, but that is beyond the scope of this work right now. However, our phonon results can be used to screen layered materials with highly negative phonon modes. As an example, we compare the phonon modes and their representation for 1T’-MoTe_2_ in Table 3. The experimental results are obtained with polarization-resolved Raman experiment^45^. We notice that our 1 × 1 × 1 conventional cell phonon results are comparable to experiments. This could be attributed due the fact that conventional cell for MoTe_2_ was large enough (more than 1.2 nm) enabling proper description of atomic vibrations. However, this may not be case for all the materials, because their conventional cells could be relatively small. The phonon density of states of the system is given in the supplementary information (Fig. S4). To ensure the effect of cell size, we also compute phonons for 2 × 2 × 2 conventional cell. We notice the 2 × 2 × 2 and 1 × 1 × 1 data are comparable and close to experiments. Therefore, consistency in different sizes ensures the validity of extension of the calculation into phonon description. In addition to providing the phonon modes, we also provide the phonon representation of the phonons using symmetry of the eigenvectors of phonon mode for most of the materials with the help of Phonopy package^62^. However, we manually implement all the crystal point groups in the Phonopy package that were missing before along with their Infrared and Raman activity as available at Bilbao server^63^. The phonon representation modes assignment is challenging task in experiments. As mentioned above, the experimental mode assignment is carried with angular Raman spectroscopy, which are much expensive. In this way we can assign the phonon modes with proper representation for unknown modes in literature such as for 1T’-MoTe_2_^64^. Comparison of the experimental and our DFT phonon modes show that our phonon modes could deviate up to 8% from experiment. However, our phonon representations have great agreement with experiments. Additionally, we compared the elastic constants obtained from 1 × 1 × 1 and 2 × 2 × 2 supercell for BN, BiClO and AuI as additional test cases for validation in the supplementary section (Table S8). We didn’t notice significant change in elastic constants due to size.

**Table 3:** Phonon modes for 1T’ MoTe_2_ for 1 × 1 × 1 conventional cell, 2 × 2 × 2 conventional cell with optB88 functional and polarization-resolved Raman experiment.

DFT Phonon mode 1 × 1 × 1 cell (cm^−1^)	DFT 1 × 1 × 1 Representation	Experimental modes	Experimental Representation	DFT Phonon modes for 2 × 2 × 2 cell	DFT 2 × 2 × 2 Representation
−0.13	Bu I			−0.58	Bu I
−0.06	Au I			−0.04	Bu I
−0.04	Bu I			0.06	Au I
7.36	Bu I			6.08	Bu I
25.80	Au I			24.65	Au I
31.04	Bu I			26.09	Bu I
76.66	Ag R			74.09	Ag R
84.86	Ag R	80.56	Ag R	81.54	Ag R
88.03	Bg R			87.63	Bg R
90.32	Bg R			89.60	Bg R
103.28	Bg R	96.54	Bg R	102.36	Bg R
104.17	Bg R	108.32	Bg R	102.95	Bg R
107.55	Au I			106.61	Au I
108.54	Ag R			107.12	Ag R
109.91	Au I			108.66	Au R
112.03	Ag R	112.8	Ag R	111.10	Ag R
114.06	Bu I			115.46	Bu I
122.27	Bu I			121.19	Bu I
124.29	Ag R			121.90	Ag R
126.27	Ag R	129.2	Ag R	123.58	Ag R
129.64	Bu I			126.25	Bu I
134.19	Bu I			130.53	Bu I
151.35	Ag R			150.19	Ag R
156.27	Ag R	163.32	Ag R	153.05	Ag R
175.28	Au I			176.42	Au I
175.94	Au I			176.93	Au I
184.24	Bg R			181.76	Bg R
186.30	Bg R	191.64	Bg R	185.04	Bg R
190.81	Bu I			189.22	Bu I
190.93	Bu I			189.61	Bu I
236.52	Ag R			234.90	Ag R
239.60	Ag R	248.45	Ag R	239.42	Ag R
252.88	Ag R	258.61	Ag R	250.04	Ag R
253.04	Ag R	263.34	Ag R	252.26	Ag R
264.26	Bu I			264.26	Bu I
265.13	Bu I			264.73	Bu I

Representation table of modes are also shown. I, and R stands for infrared and Raman active modes respectively.

### Web-interface

**Figure 6:**
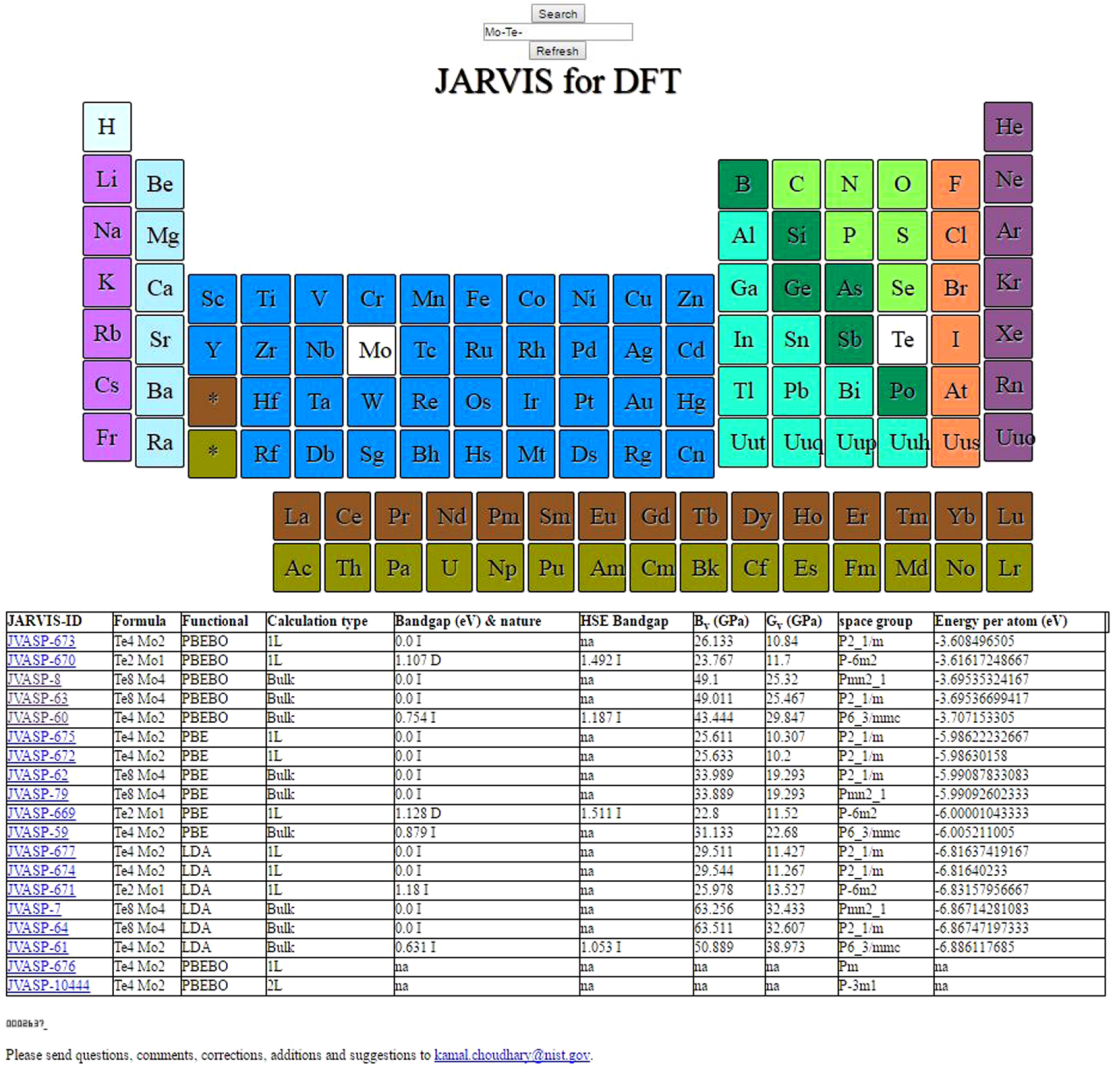
Snap-shot of the web-interface showing the example of Mo-Te as an entry.

The database is accessible through an easy to use web interface. A snapshot of the website is given in Fig. 6.

Here, for example clicking on Mo and Te and clicking “Search” button shows the options of calculations. In the first column of the results is the JARVIS-ID which leads to as detailed web-page of the calculation, second column is the formula of the structure, third column in the functional used in the calculation, fourth column is the calculation type for example 3D bulk or 2D single layer, fifth column is bandgap and its nature such as direct or indirect using LDA/PBE or optB88 functional, sixth column is the same information but for HSE06 functional, wherever available, seventh column is the Voigt bulk modulus, eighth column is the shear modulus, ninth column is the space group of the structure used in calculation, tenth column is the energy per atom of the system which can be post processed to calculate heat of formation. The energy per atom value difference between bulk and its corresponding single-layer material with consistent functional can be used to calculate the exfoliation energy. Clicking on any of the JARVIS-ID navigates to a new webpage containing detailed information of the calculation. The input files used in the calculation are provided as zipped files to ensure reproducibility of the data. Visualization of the structure is provided with the help of JSMOL^65^. The atomic structure could be rotated, and other valuable information such bond-distance, angles could be obtained interactively by simple click operations as facilitated by JSMOL. Next, convergence of the energy-cutoff and K-points are shown to ensure proper DFT optimization of the structure. Then XRD and RDF analysis of the structure is given. After that electronic structure information, such as density of states and band structure are given with total, orbital and element dos buttons to facilitate the visualization of orbital and elemental character of electronic states. After this, z-plane averaged potential is provided which can be used to calculate work-function for layered materials. Interestingly, the work-function of the 2D single layer material is close to the bulk z-plane averaged potential. This can be due to the interaction in the vdW bonding-direction is small as expected. Moreover, the elastic constant and allied phonon data are also provided for the cases where calculation has been completed. We also provide online visualization of the phonon modes using JSMOL. We provide ICSD-IDs^29^ for bulk materials at respective webpages. These ICSD-IDs can be used to find out how the materials were experimentally fabricated. All the above mentioned easy to use web-tools are provided for free on NIST’s robust CTCMS web-server http://www.ctcms.nist.gov/~knc6/JVASP.html.

## Discussions

According to functional comparison investigation of Blaha *et al*.^36, 48^, PBE generally shows 2.5% maximum error tolerance in lattice constant, thus here we assume any error that is greater than this value may be due to the extra degree of freedom such as vdW bonding. We set quite a conservative screening criterion of 5%, but it can be shown that even values as low as 3.5% can be used, such as in case of GeS, for example. If we consider transition metal chalcogenides and oxides as already known 2D-materials, hundreds of new 2D materials were found here, and the database is still growing. We expect to complete all the exfoliation energy calculations within 5 months, while computing the energetics, electronic and elastic properties will take longer, possibly up to 1 year. Our database clearly shows the 2D materials could not only be transition metal chalcogenides but they could be halides, pnictides and combination of them. The greatest advantage of the initial screening criterion is that it didn’t require any additional calculation, since ICSD and Materials Project (MP) already contains all the necessary data already publicly available. However, for energy of exfoliation calculation, we had to compute the energetics of both the bulk and single layer material using optB88 functions, which were not commonly available. Moreover, we concentrated first on the structures with available ICSD IDs because it almost guarantees the feasibility of the experimental material fabrication. Please note that the “discovery” aspect of this work is related to the identification of possible 2D materials from known bulk compounds, and that is accomplished by our strategy, even if no new 3D materials can be identified using our approach. Although the data is provided for MP only, it is being extended to other databases such as AFLOW and OQMD, and a concise database, encompassing all these above-mentioned databases for predicting 2D materials, will be available soon. The easy to use web-interface with HTML features, and publicly available input data will facilitate the data reproducibility and enhances the machine learning capabilities; a topic which will be discussed in another paper. Our database can give experimentalist a way to compare for the collected XRD and phonon data for initial experimental comparison. HSE06 band structures have recently become very popular in DFT community for their accurate results and relatively cheap cost compared to many many-body calculations. We plan to calculate the HSE06 band-structures for all the materials in our database. We have not analyzed the phonon data for many materials, however it is commonly known that finite size effect, as well as the method for phonon calculation such finite-difference and density functional perturbation theory (DFPT), play critical role in designating phonons for Raman measurements. Later we plan to carry out supercell phonon calculation for all the structures with both finite difference and DFPT to enhance quality of the phonon data. We also plan to compute thermoelectric, piezoelectric, defect energetics, interface energetics and multi-layer properties of materials with and without strain in our database. In a nutshell, C_33_ being very low, high work function and bandgaps in the range 0.5 to 3.5 eV were identified to be characteristics of 2D materials. Once again, it is worth to mention that bandgaps are generally underestimated in DFT (unless HSE06, GW or other high-level calculations are carried out), however, if a material has non-zero bandgap in DFT calculation then it must be semiconducting or insulating. Our publicly available data will allow user to select material with required characteristics at absolutely no cost. A characteristic feature of this database is a presence of both the bulk and single layer data which enables easy comparison of properties as material changes from infinite to finite size. Calculation of data for the samples comprised of multiple, but small number of layers (i.e. 2, 3, 4) is more challenging, but it will also be available in the database in the future. Despite this database still being in its initial developmental stage, it serves as a comprehensive detailed source for finding properties of the single layer 2D-layered materials in conjunction with their corresponding 3D-bulk state. Our property calculations here are our first steps in characterizing layered materials in a systematic way, much more involved property analysis is subjected to future work. After accumulating a enough data in this project, we can employ machine learning methods to predict behavior of 3D bulk as well as 2D materials in a single framework.

## Methods

### Density functional theory

**Figure 7:**
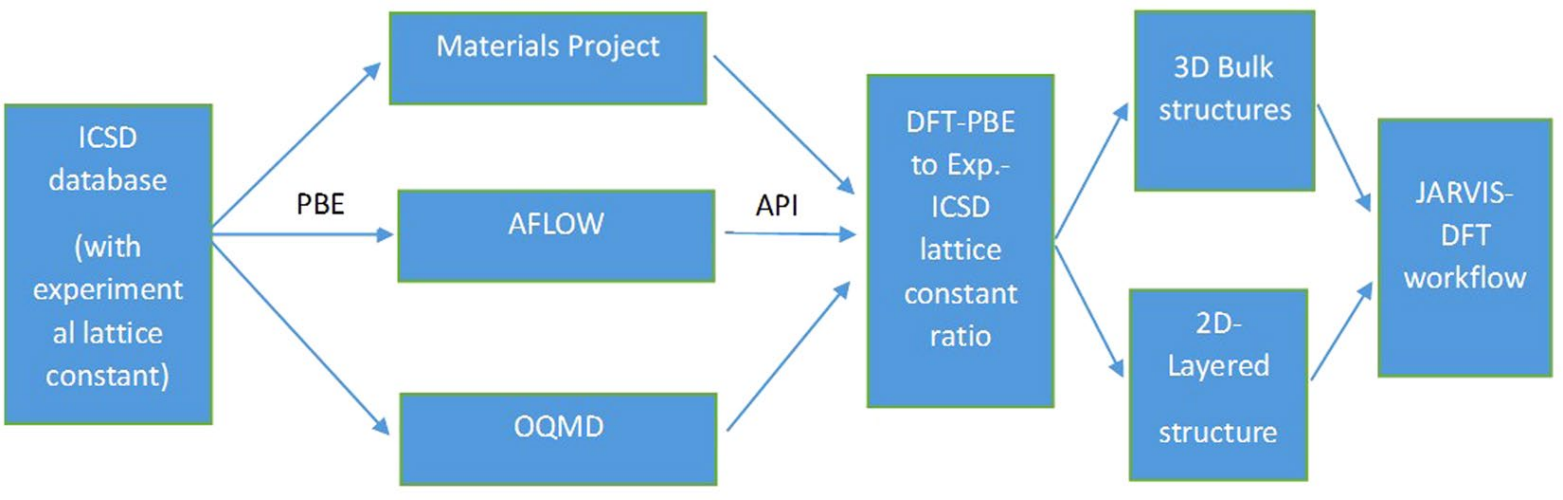
Illustration about initial screening of 2D materials is shown. DFT databases used ICSD crystallographic database to characterize bulk materials with PBE functional. PBE functional is known to overestimate lattice constant of materials which can be 2D. Based on API, materials with reasonably high PBE to Experimental lattice constant difference were screened from thousands of materials available in the DFT databases. Layered counterparts of these materials were made manually. Both bulk and layered materials were then subjected to a series of DFT calculations for their property calculation.

**Figure 8:**
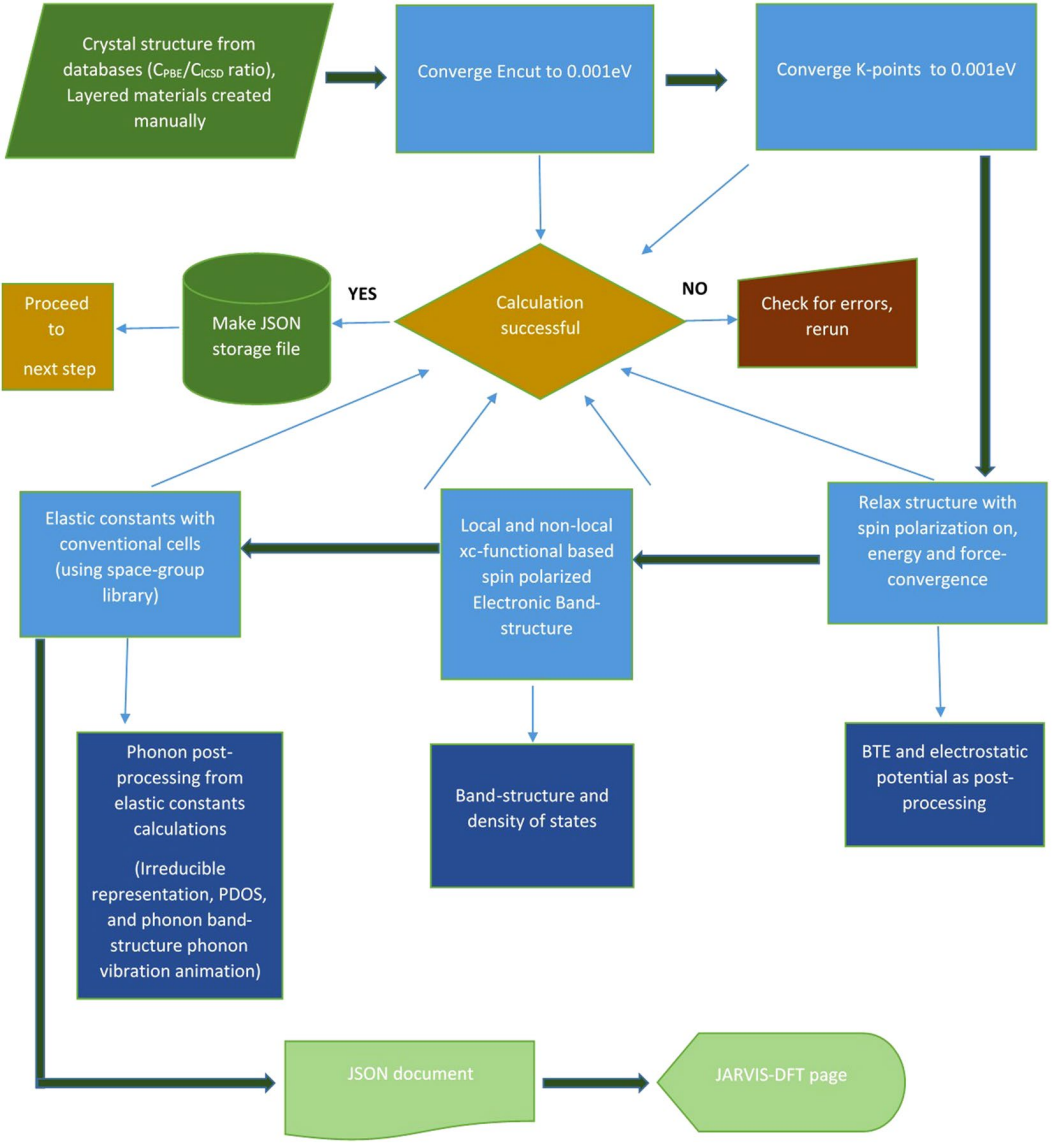
JARVIS-DFT workflow for checking the DFT convergence, carrying out property calculation, managing of calculations and building database of calculations are described. Major steps are marked with deep-green thick arrows.

All the calculations are done with Vienna Ab initio Simulation Package (VASP) software^66^. Please note commercial software is identified to specify procedures. Such identification does not imply recommendation by the National Institute of Standards and Technology. 2D bulk material structures are taken from MP, OQMD, AFLOW and ICSD or from latest research papers. These DFT databases were made with crystal structure data obtained from ICSD database. We use the ICSD lattice constants and DFT lattice constants with PBE as a guide for identifying 2D materials as shown in Fig. 7. We use 5% expansion criterion between PBE and ICSD to identify 2D materials. The geometrical input structure for input were obtained from application program interface (API) of the DFT databases mainly. The calculation set-up and handling of data is primarily tackled with Pymatgen^41^ and atomic simulation environment (ASE)^67^. The 1 L materials are made manually by separating single layers and adding vacuum padding in z-direction. At present, non-polar layered materials are computed only. We use LDA, PBE and optB88 functionals for many 2D materials to facilitate comparison of properties using different functionals. The K-point and Energy cut-off convergence is achieved with 0.001 eV tolerance with a series of DFT calculations. This convergence calculation is performed automatically. We keep increasing the Energy cut-off and K-point density of mesh until the difference between the previous and present pointy in the plot is within 0.001 eV tolerance. After achieving the tolerance criterion, we test five more points to ensure if there is some other minimum doesn’t exist. If another minimum is found, the convergence calculation is started again from the last point automatically. The K-point is chosen in an automatic mesh way as provided in VASP, which uses the reciprocal lattice vectors of the system at an increment of 5, while the energy cut-off is incremented with a step size of 50 eV. The force and energy convergence for DFT self-consistent calculations are 10^−6^ eV and 0.001 eV/Å respectively. It is to be noted that other DFT databases do not enforce force-convergence criterion which can result in incorrect description of elastic and phonon properties. After the structure optimization, we calculate X-ray diffraction (XRD), radial distribution function (RDF) plots, electronic band-structure, optical properties, and elastic constants in different sets of calculations in automated way. The elastic constants are calculated on conventional cell using space group information of the structures, while phonon-bandstructures are calculated with Phonopy package^62^. We have carried out phonon convergence for finite size effects for few systems only. The phonons obtained from conventional cell are also provided with visualization tools. We used the conventional K-points for electronic and phonon bandstructure, computational XRD as implemented in Pymatgen^41^ and AFLOW^35^. The data presented in html format as well as available in JSON format. A flow-chart for the process mentioned above is given below in Fig. 8.

### X-ray diffraction

MoTe_2_ powders and crystals were finely ground in hexane using an agate mortar; θ–2θ scans were collected at ambient temperature on a powder X-ray diffractometer equipped with general area detector using CuKα radiation (λ = 0.15418 nm). MDI-JADE 6.5 software was used to analyze the experimental data (Jade 6.5, Materials Data, Inc. Livermore, CA., 2015).

### Raman-measurement

The polarization-resolved Raman measurements were acquired by sending linearly polarized light at 532 nm through a motorized achromatic halfwave plate to control the excitation polarization. The same microscope objective (0.75 NA) was used for excitation and collection. The resulting Raman signal passed through a longpass filter followed by a motorized linear polarizer. Measurements were acquired by rotating the excitation polarization and analyzer together in either co-polarized or cross-polarized configurations. The spectra were acquired using a 500 mm focal length spectrometer with a 2400 lines/mm grating and CCD. To avoid damaging the samples an excitation power of 0.35 mW at the microscope objective was used with a 2 min spectral acquisition. The spectra were calibrated using an Ar lamp, and the laser frequency was measured with a wavemeter.

## Conclusions

In this work, we have created a new publicly available database specially dedicated to 2D materials. Our database provides detailed analysis of all the bulk and layered materials with appropriate vdW functional. Energy of exfoliation, bandgap, work-function and elastic constant values are presented and can be used to easily and consistently compare the materials of interest. All the database and the input files are provided in public domain for free to facilitate reproducibility of the DFT results. We also mention all the approximations used in the calculation on the webpage to enable the reader understand the limitations of the results. With the help of the data from our repository, we have demonstrated the effectiveness of a simple criterion to identify 2D materials based on lattice constants that can be used in predicting new layered materials. We have predicted hundreds of novel materials which satisfy both the lattice difference as well as exfoliation energy criterion to deem them as 2D materials, and the database is still expanding. Experimental synthesis and characterization of these 2D materials could reveal a vast range of material properties and phenomenon. All the bulk material calculation webpages are hyperlinked to Materials-Project and AFLOW databases to enable comparison of properties in different databases. We believe our new predicted 2D materials will be advantageous for many material science problems.

## Electronic supplementary material

Supplementary information
