# Supplementary material for: High-throughput Identification and Characterization of Two-dimensional Materials using Density functional theory
Source: Sci Rep. 2017 Jul 12;7:5179. doi: 10.1038/s41598-017-05402-0 (PMC5507937; doi:10.1038/s41598-017-05402-0)
Supplement: Supplementary file 1 — Supplementary information [file 41598_2017_5402_MOESM1_ESM.pdf]

Supplementary information: High-throughput Identification and Characterization of Two-dimensional Materials using Density functional theory

Kamal Choudhary[1], Irina Kalish[1], Ryan Beams[1], Francesca Tavazza[1]

1. Materials Science and Engineering Division, National Institute of Standards and Technology, Gaithersburg, MD 20899, U.S.A

Table S1 Prototype distribution of predicted layered materials

Prototype	Materials	#
ABCD	AgCSN, AgCNO, AgCNO, SmCuSeF, HgHOF, SmFeAsO, LaFeAsO	7
ABC_2D_4	Ta2InCuTe4	1
ABC_2D_6	VAg(PS3)2, VAg(PSe3)2, GaAg(PSe3)2, ScAg(PS3)2, YbNa(PS3)2, Yb2TbPrS6, ScAg(PSe3)2, ErAg(PSe3)2, TmAg(PSe3)2, TlIn(PSe3)2, InAg(PSe3)2, Ba2UCuO6, InCu(PSe3)2, InAg(PS3)2	14
ABC_2D_7	RbVP2S7, CsVP2S7, ZnP2PbO7, CaCrP2O7, BaCoP2O7	5
ABC_2D_8	KAl(SO4)2, RbFe(MoO4)2, KV2SbO8, YbK(WO4)2	4
$A_2B_3C_{24}$	Re2(PCl8)3	1
$A_2B_2C_3D_{14}$	K2U3(TeO7)2	1
$ABC_2D_2E_5$	K2H2IrCl5O	1
$A_3B_8C_{15}$	Sb8(PbS5)3	1
$AB_2C_6D_6$	FePb2(CN)6	1
$ABCD_3$	LiSO3F, BaBOF3, KHCO3	3
$ABCD_2$	CsSO2F, RbSO2F	2
$ABCD_4$	YbKSiS4, NaNdSiO4, KBaPS4, NaYSiO4, KBaPSe4, EuRbPO4, SrAlBO4	7
$ABCD_6$	LiSmAlF6	1
A_3B_7	Ta3I7	1
A_6B_{11}	Pr6O11, Nb6I11	2
A_3B_5	Yb3Si5, Fe5Si3, Yb5Ge3	3
A_3B_8	Sn3F8, Au3F8, Nb3Cl8	3
ABC_3D_4	Al4CN3O	1
$AB_2C_9D_{10}$	NdP2H9O10	1
AB_6C_6	Ag(TeMo)6, Zr(TeCl)6	2
$A_2B_4C_{11}$	Ta2Tl4S11, Au2Se4O11	2
$A_3B_4C_8$	Li4V3O8	1
$A_2B_5C_{13}$	Na5Zr2F13	1
AB_6C_8	W(S4Cl3)2, Hf(Te4Cl3)2	2
$A_3B_4C_5$	Li3Ce5Ge4, Re3Te4Cl5	2
$A_3B_4C_4$	Ce3(CuGe)4	1
$A_3B_4C_6$	P4(SeO2)3	1
AB_3C_8	SbI3Cl8, SbS8Cl3, La3ReO8, RuS3Cl8	4
AB_3C_9	Sn3BF9	1
AB_5C_{11}	TlHg5Cl11	1
AB_3C_4	Tl3PSe4, Hg3AsO4, Tl3PO4, Tl3SbS4, Tl4SnS3, Tl3AsO4,	14

	Rb3AsSe4, SbN3Cl4, CS3N4, P3Se4I, Hg3TeBr4, Hg3TeCl4, Hg3PO4, Ag3PSe4	
AB_3C_5	CsB3O5, V5P3N, Rb5GeP3, Ge3Mo5C, Cs3CoBr5	5
AB_3C_6	Rb3TlF6, Rb3YF6, Cs3YF6, Li(TiSe2)3, Li(TiS2)3, Pb3SO6, K3AlH6, NbHg3F6, Nb3GeTe6, Hg3SO6, BaMn3O6	11
AB_3C_7	Nb3TeCl7, Nb3SBr7, Ta3SeI7, Ta3TeI7, CuMo3I7, Nb3TeI7, AgW3Br7, Ta3SBr7	8
AB_3C_3	Tl3BSe3, Ga3Te3I, Al3Te3I, Tl3BS3	4
A_5B_6	S5N6	1
$A_2B_3C_4D_{12}$	Na2Cu3(GeO3)4	1
$A_6B_7C_8$	S7(N3O4)2	1
$ABCD_3E_6$	H6CN3ClO	1
$AB_3C_4D_4$	BS4N4F3	1
AB	SN, SN, HgTe, YbGa, CrS, PS, HgS, TlF, CrTe, GaN, BN, NdSb, VS, HgO, ZrTe, SnS, CdAu, CdTe, HgTe, CaSi, GaSe, CrSb, PrSb, MnNi, SiAs, SmS, GaSe, LaAg, SnS, MoN, CaSn, GaS, TlSn, AuSe, SrSi, YN, TbPt, BaO, AlSn, HgO, BN, SnS, HgS, ZnTe, GeAs, NbP, GaTe, RuC, LaSb, MnSe, TaS, SmSe, TbSe, YbSe, YbSi, TmTe, AuSe, PdO, FeTe, AlAs, TiCu, TlNi, TlPt, FeN, CdTe, TaP, PuNi, GeS, ZrN, TiSe, PtN, AlN, CoAs, CoO, USi, InSb, InS, PbO, PuSb, FeSe, DyGe, LaS, InSb, FeSe, LaAs, PbS, CeS, PuSi, NpSi, CeNi, InSb, FeSn, FeTe, PtPb, InSe, FeS, PuIn, TiSi, HgI, NiBi, CuI, BiRh, InBr, MnBi, HgCl, CuBr, BiPt, HgBr, BCl, TeI, InBi, MnH, ZrCl, CN, IBr, BiI, AuI, BiSe, SCl, SBr, PSe, ClF, SiH, NO	124
ABC_4D_{13}	KTaP4O13	1
A_3B_{11}	K3P11, K3Hg11	2
ABC_{14}	SnHgP14, HgP14Pb	2
A_7B_8	Fe7S8	1
ABC_{11}	NbXeF11	1
$A_{14}B_{23}$	Ho14Ge23	1
AB_2C_{12}	Ca(AuF6)2, Re(TeCl6)2, Zr2TeBr12, Te2OsCl12	4
$A_2B_3C_3$	Na3Li3N2	1
AB_4C_{13}	Lu4CoB13, Tb4CoB13	2
AB_4C_{11}	RbNb4Cl11	1
$AB_2C_2D_3E_8$	Ba2Ca2Cu3HgO8	1
$AB_2C_4D_{12}$	K2Cu(PO3)4	1
ABC_2	CuAgTe2, TlGeS2, CuCN2, NaAsO2, Ti2SnC, Zr2AlC, EuTl2Pd, AgSbS2, LaTe2Se, NdTe2Se, Zr2SnC, Zr2AlN, Hf2SnC, Ti2AlN, AgCO2, Nb2SnC, NbCoTe2, AgNO2, Na2LiN, CuAgO2, RbAuO2, TlCdS2, YbAgS2, YbLiO2, CdHgO2, AlAgO2, TlFeSe2, TlAgTe2, CsAgC2, LiMnSe2, NaMnSe2, Cr2GaN, RbAuC2, ZrAlPt2, TiCdHg2, YbCsSe2, LiMnTe2, NaMnTe2, TlFeS2, KAsSe2, PbCN2, TlSbSe2,	103

	YbCuS2, Zr2InC, Sc2InC, Ti2InC, NaAuO2, CdInS2, GaCo2Ni, DyCoSn2, LiCeSn2, Ta2InC, UCrC2, UFeC2, AgClO2, CsLiBr2, SiCl2O, PNCl2, Bi2TeI, PBr2N, SiCl2O, ZrTi2O, SeOF2, KAuO2, Te2AuCl, CsI2Br, LiSnS2, CCl2O, TlAgSe2, NaFeS2, Hg2IO, TlSbS2, LiBiO2, SCl2O, SBr2O, Ca2IN, CsBr2F, TeCF2, TaCoTe2, MnWN2, TaI2O, RuCl2O, OsCl2O, Hf2CoP, CuSe2Br, FeAgTe2, RbMnSe2, HgCN2, PrTe2Se, LiMoS2, RbAsO2, TiAgHg2, BaZnBi2, TbCoSn2, ErCoSn2, KBiO2, CuTe2Br, CuTe2I, CuSe2Cl, PNF2, AgTe2Au, VBr2O, SrCuO2	
ABC_3	MgGeO3, CaCO3, ZnPS3, CaSiO3, CaSiO3, RbSO3, MgSiO3, CaZrO3, SnGeS3, RbNO3, CaSiO3, CdPS3, AgPS3, FePS3, KHgF3, YAlO3, YbLaS3, CdSO3, MnPS3, TaFeTe3, HgSeO3, HgSeO3, YbLaSe3, YbPrSe3, YbNdSe3, YbSmSe3, NaMgF3, BaTiO3, RbNO3, SiCuO3, BaSiO3, TiFeO3, TiPbO3, UMnSe3, BaTiO3, CaIrO3, TiPbO3, LiSi3Pd, YbCeSe3, UPdSe3, YbLaS3, SrRuO3, CsNO3, UFeS3, CCl3F, MoNCl3, NbSeBr3, DyCoO3, SmCoO3, PrMnO3, EuMnO3, HgPS3, CuCO3, PCl3O, CsSnCl3, NbCl3O, HgBrO3, NbTeBr3, RbTiCl3, CsTiCl3, GeH3Cl, CuSe3Br, CBr3F, LaTlO3, RbVBr3, BrNO3, AsCl3O, CsPdCl3, HgClO3, KAuI3, AlSiTe3, UGa3Ni, WI3O	73
AB_5C_6	Na5ReO6, SbI5F6, AsBr5F6, AsI5F6	4
AB_5C_5	S5N5Cl	1
$A_2B_6C_7$	Y6C2I7, Be2Te7Cl6	2
$A_2B_3C_5$	Ta2Te5Pd3	1
AB_5C_8	KSb5S8, SnMo5O8, PrMo5O8, SmMo5O8, NdMo5O8, CaMo5O8, TlAs5S8	7
AB_2C_{13}	Ti2PCl13	1
$AB_2C_4D_8$	Ba2Y(CuO2)4, K2Pd(NO2)4, Ba2Y(CuO2)4, H8S(NO2)2	4
$AB_2C_4D_6$	Y2Si4CN6	1
$A_2B_2C_3D_5$	Sr3Fe2Br2O5	1
$A_2B_5C_5$	Na5Co2S5	1
$A_2B_2C_7$	Au2Se2O7, K2U2O7, Bi2Se2Cl7, Hg2Mo2O7	4
$A_2B_2C_5$	Sn2Sb2S5	1
$A_2B_2C_3$	S3(NO)2, P2S2O3, K2Cd2O3, Sr2Cu2O3, S3(NCl)2, Ge2S3I2, Tl2Sn2S3, U3(SiC)2, Cs2Au2Se3	9
$A_2B_2C_9$	As2S2O9, C2S9N2	2
AB_2C_5	CuSe2O5, PdSe2O5, K5CuAs2, U2FeS5, Sr2InI5, GeBi2O5, AlPd5I2, Ta2NiS5, Nd2GeO5, Si2NCl5, ZrTe2Br5, NaSn2Cl5, Se2NCl5, NaLi5N2, CsPd2Cl5, Bi2CO5	16
AB_2C_4	Cs(SbSe2)2, Ba2ZrS4, Hg(CO2)2, Tl2SeO4, Cd2SiO4, Zn(GaS2)2, Ag2SO4, Hg2NO4, Hg2SO4, Hg2SeO4, Li2UO4, Cs(SbS2)2, Cu2WS4, Cu2GeO4, K(SbSe2)2, Rb(SbSe2)2, Ag2SeO4, Hg2GeO4, Nd2CuO4, Zn(GaSe2)2, Pr2CuO4, K2RuO4, As2PbS4, In2CuO4,	43

	Er(Fe2Ge)2, Yb(NdS2)2, Sr2UO4, Ba(InTe2)2, Zn(InS2)2, Sm(Ni2P)2, Yb2CaO4, Na2SO4, Cu2HgI4, Rb2CrCl4, H2SeO4, Mg(SbO2)2, Cr2NiS4, C(Se2Br)2, Cs2HgI4, Cu2SO4, Sr(BiO2)2, Rb2ZnI4, Na2MnO4	
AB_2C_7	Ta2PtSe7, Ta2SnO7, RbDy2Cl7, KAl2Br7	4
AB_2C_6	TbRb2F6, Au(OF3)2, TbLi2F6, K2UF6, K2HfF6, Ta2PdS6, Ta2PdSe6, K2HfF6, MgTa2O6, Hf(PS3)2, Na2GeF6, Na2SiF6, Na2TiF6, Rh(OF3)2, K2TeBr6, As2SO6, Si2H6O, Rb2TeI6, Pd(SCl3)2, Pd(SeCl3)2, K2PtI6, Pt(SCl3)2, Nb2Te6I, Pd(PbCl3)2, Pd(PbBr3)2, Si2H6S, Si2H6Se, Ta(TeBr3)2, Ta(TeCl3)2, Pd(Se3Cl)2, Pd(SeBr3)2, S(IO3)2, Tl2TeBr6, Tl2TeI6	34
$A_3B_3C_4$	S4(BrN)3	1
AB_2C_3	SrCu2O3, Rb2CO3, TlAg3S2, Li2PrO3, TlPd2Se3, Tl2GeS3, CaCu2O3, U2AlCu3, Tl2SiS3, TlTe3Pt2, TlPt2S3, CsAu3S2, RbAu3Se2, CsAu3Se2, TaNi2Te3, YbCo3B2, UFe3B2, PrAl2Ni3, U2Al3Os, ZnNi3Sb2, Ti3PO2, LaAl2Ag3, Ag2SeO3, Tl2TeS3, RbAg3S2, CsCu2Cl3, CsAg2I3, Sc2Co3Si, MoS2Cl3, Na2PrO3, Y2NCl3, Se2NCl3, OsO3F2, Se2Br3N, S3N2Cl, Hg2TeO3, P2SeO3, Ca3SiBr2, CsH3O2, Hg2SeO3, TaMn2O3	41
AB_2C_2	Ba(TlHg)2, Sr(MnAs)2, Sr(FeAs)2, Yb2CdPd2, Sr(MnP)2, Ca(FeP)2, Nb2CS2, Ca(MnP)2, Na2CoS2, Ba(CuAs)2, Yb(MnAs)2, Zr(NiP)2, Yb2AlSi2, Sc2AlSi2, Yb2Ge2Ir, Cs(MnP)2, Np(CrSi)2, Tl(FeS)2, Cs(FeSb)2, Yb2SO2, Yb2MgSi2, Yb2SeO2, Pu(CrSi)2, Ba(MnP)2, Np2InNi2, Li2NdSb2, Eu(MnAs)2, Pu(FeSi)2, Ag2PbO2, Np(CuGe)2, Ce2SbO2, U(CoP)2, Li2CeSb2, Eu(FeAs)2, Sr(MnGe)2, Yb2InPd2, Ce(SnIr)2, Eu(MnP)2, Np(CoGe)2, Yb2S2O, Ba(MnGe)2, Li2PrSb2, Tb2CoGe2, U2Ni2Sn, C(ClF)2, Ce2BiO2, Sn2SI2, H(CO)2, Xe(OF)2, Nb(SeCl)2, Nb(SCl)2, Mo2SBr2, S(ClO)2, B2PCl2, Sc2CCl2, Sc2NCl2, Ta(NiTe)2, Sb2S2O, B2AsCl2, S2N2Cl, W(ClO)2	61
$ABCD_2E_2$	CS2NClO2, TeSeS(NCl)2	2
AB_2C_9	AlP2I9, GaP2I9	2
AB_2C_8	Pd(AuF4)2, Mg(AuF4)2, Zn(ReO4)2, Al2CuCl8, Nb(PS4)2, Al2CdCl8, Pt(SCl4)2, Ga2CuCl8, Re2PbO8, Eu(AlCl4)2, Ba(AlCl4)2, W2CCl8, Ga2PdBr8, Ga2PdI8	14
$A_2B_4C_5$	Ag4Bi2O5, Sb4S5Cl2, Ta2Mn4Si5	3
$ABC_2D_4E_6$	Hg6S4IBr2Cl	1
$AB_2C_3D_{12}$	Cs2Mo3SeO12	1
$A_2B_4C_9$	Sr4Nb2O9	1
A_2B_7	Tc2O7, K2Hg7, Rb2Hg7	3
AB_4C_6	Mg(C2N3)2, Cd(C2N3)2, Sm4Cl6O, P4SeO6, Hf(Te2Cl3)2, Hf(Se2Cl3)2, Ir(Cl2F3)2, Zr(Se2Cl3)2	8
AB_4C_5	Ag5SbS4, H4SO5	2
$A_2B_5C_6$	Pb5(SI3)2, Hg5(SbI3)2	2

A_2B_3	B2O3, P2O3, As2S3, As2Se3, Tm2S3, As2O3, As2O3, Sb2O3, Sb2Se3, Sb2S3, Yb2Se3, Nd2O3, Tb2Si3, Mn3N2, Ga2O3, Ca3N2, Yb2S3, Ce2O3, Gd2O3, Th3Si2, Bi2Se3, Au2O3, Te3Cl2, Mn3As2, Hf3Te2, Br2O3, Al2Te3, Er3Ru2	28
ABC_4D_{12}	Cs4BiSbCl12	1
AB_4C_8	K4GaAu8	1
AB_6C_{19}	LiNb6Cl19	1
AB_6C_{18}	W6CCl18	1
A_2B_9	V2Se9	1
$A_4B_4C_{11}$	Sm4Mo4O11	1
ABC_3D_6	TlHg(AsS2)3	1
ABC_3D_8	LiYMo3O8, LiScMo3O8	2
ABC_4	LiYF4, YbTaO4, LuTaO4, ScPS4, YNi4B, KSO4, SbAsO4, AsPO4, ZrTiTe4, YbLiF4, CaSO4, YbPO4, AlPO4, ZrGeTe4, UOF4, TaTe4Ir, CrWO4, KInBr4, LiAlCl4, BiPO4, PAuCl4, SbCl4F, NaBH4, KBH4, ReCl4O, AlPS4, WSCl4, BPS4, CsReBr4, TaCl4F, VNCl4, LiGaBr4, LiGaCl4, TlAuCl4, NaInBr4, NaInI4, RbBrF4, TaTe4I, Te4MoBr, KGaI4, GaCuI4, TlFeBr4, KAuI4, KAuBr4, AuSO4, Li4HN, LuPS4, PAuS4, GaPS4, WBr4O, WCl4O	51
ABC_5	KAuSe5, KAuS5, TaNiTe5, NbNiTe5, TaTe5Pt, AuSCl5, TbCsF5, NbTe5Pd	8
ABC_6	TbBaF6, TiAgF6, USO6, RbPSe6, KPSe6, UMoO6, AlICl6, RbPaF6, AlSbI6, NaNbCl6, ZrFeCl6, HfFeCl6, CsSbS6, CaThBr6, SrThBr6, ZrSnCl6, CsWCl6, AlBiBr6	18
ABC_7	GaSeBr7, HS7N, FeSeCl7, TeAuCl7, AuSeCl7, SICl7, AlTeI7, AlSeBr7, GaTeI7	9
AB_2C_{10}	Te2PdCl10	1
$A_2B_3C_9$	Ge3Sb2O9, Zn2(PS3)3, Rb3Mo2Cl9, Rb3Mo2Br9, Ba2V3O9	5
$A_2B_3C_8$	P2Pd3S8, Cu3(PO4)2, Ta2Pt3Se8, Zn3(AsO4)2, Ta2Pd3Se8, Mg2Mo3O8, Zn2Mo3O8, Al2Hg3Cl8, Ta2Zn3O8, Si3(Cl4O)2	10
$A_2B_3C_7$	La3Ni2Sn7	1
$A_2B_3C_6$	I2(OF2)3	1
ABC_4D_4	TcH4NO4	1
$A_2B_3C_4$	Cs2Pt3Se4, Ca3(AlAs2)2, Tl2Au4S3	3
ABC_8	BNF8, NbAlCl8, HS8N	3
ABC_9	MoPCl9, TiPCl9, RePCl9, NbTeCl9	4
AB_4C_7	P4SO7, P4SeO7	2
A_4B_5	As4S5, P4S5, Pu5Rh4, P4Se5, P4S5, V5Te4, Mn5As4	7
A_4B_7	Ag7Te4	1
$AB_3C_6D_6$	CoAg3(CN)6	1
A_2B_5	Al2Te5, Nb2O5, Rb2S5, K2S5, K2Se5, Pr2I5, Pr2Br5, Se2O5, P2Se5, La2I5	10

AB_4C_4	Na4CO4, Ta4SiTe4	2
$ABC_2D_4E_{14}$	Na2ZrCo(P2O7)2	1
A_3B_4	P4S3, P4S3, Ag3O4, Fe3O4, As4S3	5
AB_3C_{12}	Nd(AlBr4)3	1
AB_3C_{13}	K3AuSe13	1
AB_3C_{11}	IrS3Cl11	1
AB_2	WS2, Rb2Te, TiO2, GeO2, Te2Mo, PtO2, SnSe2, VSe2, SeO2, PtS2, TmSi2, B2Mo, USi2, PuGe2, YbC2, LuSi2, PtSe2, SiNi2, Li2S, SnS2, ZrS2, Rb2Te, SiS2, MoSe2, Co2Ge, TaS2, HoSi2, UHg2, WSe2, Cu2Te, TaS2, ZrSe2, NdGe2, ErSi2, TiSe2, CS2, TeO2, HgO2, PdSe2, S2N, MoS2, ScSi2, Mn2Sb, SiO2, SiO2, Te2Mo, TaGe2, GeS2, GeF2, BaSi2, AgF2, PtO2, HoSb2, TaSe2, Na2S, BaF2, Ta2Se, TcS2, VS2, RbHg2, SnS2, SiP2, NbS2, GeSe2, ThGe2, B2W, YbB2, YGe2, NbSe2, B2Au, GeO2, TaSe2, LuPb2, PrGe2, AgB2, Zr2Np, KAg2, ThMn2, SmGe2, YSi2, DySi2, Al2Cu, Sr2Si, YbGa2, Er2Mg, Tb2Mg, Cs2Pt, PdS2, GeAs2, WO2, LaGe2, NpGa2, EuSi2, Pu2Co, GdSi2, TaO2, CeGe2, GdO2, OsN2, KO2, UTe2, Cr2As, Co2As, Te2W, HgCl2, FeBr2, ZnCl2, PbI2, IO2, HgI2, HgI2, LaI2, MgI2, ClO2, CuBr2, BeCl2, HgBr2, BCl2, MgH2, SrH2, CaH2, MnO2, CrI2, TmCl2, Te2Br, Te2I, TiBr2, PdBr2, GeI2, CdBr2, W2N, MnI2, PbBr2, TiCl2, SCl2, LaSe2, CdI2, MnBr2, NdCl2, PtI2, GaBr2, Br2O, LaBr2, NdI2, SiCl2, SnCl2, PI2, SmCl2, WBr2, MoBr2, TmI2, SnBr2, Sc2C, KrF2, DyBr2, CaI2, CoBr2, MgBr2, BeBr2, Mn2Au, CuCl2, Ag2S	162
AB_3	NdTe3, Na3As, ZrSe3, Ag3Sb, SO3, YF3, Mn3As, Cu3As, ReB3, ErF3, SmTe3, USe3, TiS3, ZrS3, YAl3, Mg3Au, K3N, PrTe3, US3, TiCu3, PdF3, PrB3, Pu3Al, RhF3, YbF3, NaN3, RuCl3, MoCl3, ClO3, ZrCl3, SbCl3, CsI3, RuBr3, BiCl3, BCl3, BI3, TiI3, BBr3, PCl3, UI3, ZrBr3, TiI3, AsCl3, ZrI3, AlBr3, TbCl3, RuBr3, BrF3, MoBr3, IO3, AsBr3, AlCl3, AlCl3, GdBr3, AuBr3, PBr3, IrBr3, SbBr3, YCl3, SeO3, PI3, CrCl3, CsBr3, AuCl3, IrCl3, CrBr3, RhCl3, RhBr3, PuBr3, NdBr3, SmBr3, PuI3, LaI3, TiBr3, PtI3, RbI3, TlI3, DyCl3, SiI3, TaSe3, Si3Ir, Zr3Ag, MgPt3, AlI3, GaCl3, GaBr3, GaI3, InPd3	88
AB_4	PbF4, NdAl4, PrAl4, YbAu4, TbB4, Fe4P, XeF4, SnBr4, SiH4, UBr4, ReCl4, TiBr4, PuPt4, TcCl4, OsBr4, SiCl4, HfCl4, TaCl4, SnCl4, TiCl4, NbCl4	21
AB_5	CeP5, ZrTe5, HfTe5, SmFe5, UF5, BaAg5, UF5, CaPd5, CePd5, PBr5, SbCl5, PCl5, NbCl5, UCl5, TlP5, PaCl5, BiF5, UTe5, NbBr5, UBr5, TaCl5, AsCl5, LuMn5, NbI5	24
AB_6	UF6, SF6, WCl6, UCl6, RbTe6	5
AB_8	S8O, Ag8S	2
A_4B_{15}	C15S4	1
$AB_3C_3D_6$	AsS3(ClF2)3	1
$A_4B_4C_5$	Yb5(LiGe)4	1
ABC	PPdSe, ScOF, SNF, CuAgS, YbOF, KAuS, PPdS, SbOF, NbFeB,	121

	USiS, YbSiCu, YbLiGe, CuAgS, YbAsPd, RbAuS, ErCuGe, RbAuSe, YbAgGe, YbPPt, KAuSe, DyBC, BaLiSi, YbSiIr, SrLiP, BaLiP, BaLiAs, BaAgSb, SrAgSb, ErGaCo, YbCuSb, SrSbAu, HoMnGa, YbSnRh, NdTeF, YbMnGe, MnCuAs, UFeSi, KCN, LaInPd, USiRh, LaMnGe, MnCuP, NaMnP, RbMnAs, UGeRh, PrMnSi, KMnP, YbGeRh, TbMnSi, USnPd, CaHgPb, YbMnGe, CaMnSi, ZrNiP, CeCoGe, TbFeSi, RbMnP, YbMnSi, DyMnSi, ScFeSi, BiClO, TeRhCl, BiTeI, PbIF, SbSBr, BiIO, TiClO, SbSeI, CrSBr, TiBrO, SbSI, BiSeI, SbSI, SrIF, ZrIN, NdBrO, BiBrO, BiSCl, BiSBr, BiSI, LiBH, CaHI, SrHI, CaHBr, AuSeBr, BSBr, LuSBr, TmIO, GaTeCl, CNCl, ErSCl, InClO, TiIN, TiBrN, TiNCl, AlClO, SNCl, PuBrO, CBrN, BaBrCl, SbTeI, ThBrN, ThIN, ErSeI, DySBr, DySI, BiTeCl, AuCN, InTeI, InTeBr, TlAgSe, GeAsSe, PrIO, SmBrO, HSN, GdBrO, PdSCl, BaAgBi, LaIO, CrBrO, VBrO	
$AB_3C_5D_{12}$	Ca5P3O12F, Sr5P3O12F, Sr5P3ClO12, Sr5P3BrO12	4
$AB_2C_3D_3$	Dy3Mn3Ga2Si, Tb3Mn3Ga2Si	2
$AB_{20}C_{26}$	B20H26O	1
$AB_2C_3D_6$	NaTl3(SO3)2	1
$A_4B_8C_9$	Re4Cl8O9	1
ABC_2D_{10}	RbNb2PS10	1
A_8B_9	As8S9	1
ABC_{10}	TaPCl10, SbClF10	2
$ABC_4D_4E_4$	CoHgC4(SeN)4	1
$A_3B_7C_7$	Re3(SBr)7	1
$AB_3C_4D_{12}$	KMn4(PO4)3	1
$AB_2C_2D_2$	C2Se(SN)2, NiC2(SN)2, SeS2(NO)2, Sr2Co(BrO)2	4
$AB_2C_2D_4$	Cs2Pd(IBr2)2	1
$AB_2C_2D_7$	Ca2MgSi2O7	1
$AB_2C_2D_6$	Tl2Sn(AsS3)2, ZrP2(HO3)2	2
$A_7B_{10}C_{24}$	Ta7(Te12I5)2	1

Table S2 Space-group distribution of predicted layered materials

Space-group	Materials	#
$P4_2/mmc$	CsAgC2	1
$I4_1/a$	LiYF4, YbLiF4, YbPO4, TcH4NO4, CCl2O	5
$P6_3mc$	GaN, YN, PtO2, TaSe2, ErCuGe, SnS2, SmSe, TbSe, YbSe, YbCuSb, ZrN, Mg2Mo3O8, Zn2Mo3O8, CdBr2, CdI2, Sm4Cl6O, BiTeCl, MnWN2, Ta3SeI7, Ta3TeI7, LaTlO3, Nb3TeI7, LuMn5, Na2MnO4	24
$P6_3/mmc$	WS2, CrS, Te2Mo, BN, Na3As, VS, ZrTe, MoSe2, CrSb, Co2Ge, WSe2, GaSe, TaS2, GaS, MoS2, Ti2SnC, Zr2AlC, Zr2SnC, Zr2AlN, Hf2SnC, Ti2AlN, Nb2SnC, Mn2Sb, U2AlCu3, ReB3, YAlO3, BN, YbSiCu, Na2S, AlAgO2, GaTe, NbS2, MnSe, Cr2GaN, TmTe, AuSe, FeTe, U2Al3Os, ZrAlPt2, AlAs, BaAgSb, SrAgSb, YAl3, Mg3Au, TlNi, SrSbAu, FeN, YbCsSe2, KAg2, ThMn2, Sr2Si, PtN, AlN, Cs2Pt, K5CuAs2, BaSiO3, Zr2InC, Sc2InC, Ti2InC, CaHgPb, ZrNiP, PtPb, Ta2InC, NiBi, BiRh, MnBi, BiPt, SbCl5, SrH2, MnH, CaH2, Sc2Co3Si, RbTiCl3, CsTiCl3, Ca2IN, LaBr2, Rb3Mo2Cl9, Rb3Mo2Br9, Si3Ir, BaAgBi	80
$P\bar{3}c1$	Cu3As	1
$P\bar{6}m2$	GaSe, AlSn, YbPPt, BaLiSi, RuC, SrLiP, BaLiP, BaLiAs, TaS	9
$P4/mbm$	CaSiO3, Yb2CdPd2, Yb2AlSi2, Sc2AlSi2, Yb2MgSi2, TbB4, Np2InNi2, Th3Si2, Yb2InPd2, U2Ni2Sn	10
$P4_2/ncm$	AuI, Hf(Se2Cl3)2	2
$I\bar{4}2d$	UF5, GeS2, GeSe2, UOF4, UFeC2	5
$I\bar{4}2m$	Ta2InCuTe4	1
$P2_1$	P4S5, Hg(CO2)2, YbKSiS4, S3(NCl)2, AlICl6, TeCF2, K2U2O7, C2S9N2, Ag2S, Ba2V3O9	10
$P321$	RbSO3, K2UF6, KAl(SO4)2, Nd2O3, PdF3, Ce2O3, RhF3	7
$P2_12_12$	As2SO6	1
$P4_2/mbc$	SeO2, Mg(SbO2)2, Re2(PCl8)3	3
$R\bar{3}m$	CoO2, Bi2Se3, Sb2Te3	1
Pbcn	K3P11, PPdSe, TlAg3S2, PPdS, GeO2, B2PCl2, Si2H6S	7
Pbcm	TlF, PBr5, SbClF10, RbAsO2, Zr(TeCl)6	5
Fdd2	Hf(PS3)2, C(ClF)2, Au2O3, SCl, S(ClO)2	5
Pbca	PdSe2, NaAsO2, PdS2, CCl3F, ClO2, BCl2, TeSeS(NCl)2, TmCl2, CN, Tc2O7, TcCl4, CsReBr4, AlP2I9, OsBr4, Se2NCl3, Hg3TeBr4, Hg3TeCl4, S3N2Cl, CuMo3I7, GaP2I9, HgCN2, AuSO4, DyBr2, W2CCl8	24
$P6_322$	LiSmAlF6	1
Cmme	TbBaF6, RbPaF6	2

Cmmm	Li2PrO3, Ba2Y(CuO2)4, DyBC, La3Ni2Sn7, TlSbSe2, Ba2Y(CuO2)4, ZrTi2O, PuPt4	8
$P6_3cm$	RbVBr3	1
Ama2	NdTe3, PrTe3	2
$P6_3/mcm$	Fe5Si3, V5P3N, Yb5Ge3, K3N, Ge3Mo5C, RuCl3, ZrCl3, RuBr3, TiI3, ZrBr3, ZrI3	11
P4/n	PCl5, Bi2Se2Cl7	2
Pmmn	CaCO3, SrCu2O3, CaCu2O3, TiCu3, RbNO3, PrMnSi, CsNO3, TiClO, CrSBr, TiBrO, ZrIN, TiI3, RuBr3, MoBr3, LuSBr, CNCl, ErSCl, InClO, TiIN, TiBrN, TiNCl, AlClO, CBrN, ErSeI, DySBr, DySI, Tl2Au4S3, AsCl5, CrBrO, VBrO	30
Pmmm	TiPbO3, Fe4P, KAuO2	3
P4/mmm	YbGa, NdSb, PrSb, MnNi, LaAg, TlSn, SrSi, KHgF3, LaSb, RbAuC2, TiCdHg2, TiCu, Al2Cu, PuSb, BaTiO3, FeSe, Pu3Al, LaAs, GaCo2Ni, PuIn, Ba2Ca2Cu3HgO8, CsBr2F, NaLi5N2, TiAgHg2, Zr3Ag, MgPt3, InPd3, SrCuO2	28
Pmma	CdAu, SiCuO3, CaThBr6, SrThBr6, LiNb6Cl19, RbNb4Cl11	6
Aea2	SBr	1
Pc	RbNb2PS10, KSb5S8, GaSeBr7, As2S2O9, Al2CdCl8, ZrSnCl6, AlTeI7, AlSeBr7, GaTeI7	9
P2/m	ZrTiTe4, AgTe2Au	2
P6mm	AuCN	1
$I\bar{4}$	CoHgC4(SeN)4, SbCl4F, TaCl4F, GaCuI4	4
P2/c	YbTaO4, LuTaO4, VAg(PS3)2, Hg2SO4, Hg2SeO4, NbSeBr3, ReCl4, NbTeBr3, K3AuSe13, HfCl4, Eu(AlCl4)2, Ba(AlCl4)2, As8S9, Hg2Mo2O7	14
Pm	Na3Li3N2	1
Pccn	Sb2O3, SrAlBO4, Zr(Se2Cl3)2	3
Pcca	AgClO2	1
Pbam	Ta2Pt3Se8, SiP2, GeAs2, Ta2Pd3Se8, Sm4Mo4O11, Ta4SiTe4	6
I4/mcm	Sr2InI5, RbBrF4, Cs3CoBr5	3
$P3_12_1$	HgTe, HgS, CdTe, HgO, ZnTe, Nd(AlBr4)3	6
$P\bar{3}m1$	PtO2, SnSe2, VSe2, PtS2, PtSe2, SnS2, ZrS2, TaS2, ZrSe2, TiSe2, P2Pd3S8, Sr(MnAs)2, Sr(MnP)2, Nb2CS2, Ca(MnP)2, TlPd2Se3, TlCdS2, NaTl3(SO3)2, Li(TiSe2)3, SF6, TlTe3Pt2, TlPt2S3, CsAu3S2, RbAu3Se2, CsAu3Se2, VS2, Yb(MnAs)2, NbSe2, TaSe2, ZnNi3Sb2, Yb2SO2, Ca3N2, Yb2SeO2, Ti3PO2, Na2GeF6, Na2SiF6, Na2TiF6, RbFe(MoO4)2, Li(TiS2)3, Eu(MnAs)2, Gd2O3, CdInS2, Zn(InS2)2, Eu(MnP)2, Na2SO4, FeBr2, PbI2, WCl6, MgI2, UCl6, ZrP2(HO3)2, AlCl3, LiSnS2, TiBr2, BiSe, GeI2, W2N, MnI2, TiCl2, MnBr2, Sc2CCl2, Sc2NCl2, Nb3TeCl7, FeAgTe2, TmI2, SiH, Sc2C, Nb3Cl8, CaI2, CoBr2, MgBr2, K2Hg7, Rb2Hg7	73
$C222_1$	Yb(NdS2)2, Ho14Ge23	2

P1	Zn(GaS2)2, Tl3SbS4, Sr2Cu2O3, KAsSe2, Zn(GaSe2)2, Na2ZrCo(P2O7)2, MnO2, FeSeCl7	8
$P4_2nm$	S2N	1
$P4_2/nmc$	CaSiO3, HgI2	2
$P6_{222}$	TaGe2	1
Pmm2	Ag3Sb, CuAgTe2, CdTe, InSb	4
Imm2	AgNO2, TiSi, Ag8S, Bi2CO5	4
$I4_1/amd$	PuGe2, NdGe2, YbAgS2, YbLiO2, Cu2GeO4, NbP, ThGe2, YGe2, PrGe2, TaP, SmGe2, YSi2, DySi2, CeGe2, Cs4BiSbCl12, CsH3O2	16
$Cmc2_1$	Ag5SbS4, CuAgS, Al4CN3O, NaMgF3, ZrGeTe4, USnPd, HgI2, HgBr2, BrF3, IBr, GeH3Cl, Mn5As4	12
C2/m	SiAs, HgO2, AuSe, ZnPS3, CdPS3, AgPS3, FePS3, LiSO3F, NiC2(SN)2, Na5ReO6, Ta2PdS6, Ta2PdSe6, MnPS3, CdHgO2, Al2Te5, GeAs, TlFeSe2, Yb2Ge2Ir, Ta2PtSe7, RbAg3S2, InSe, Tb2CoGe2, NaN3, MoCl3, TeRhCl, Pb5(SI3)2, Cs2Pd(IBr2)2, CuBr2, NbCl5, Y6C2I7, AlCl3, UBr4, GdBr3, CrI2, Nb(SCl)2, TlHg5Cl11, Na5Zr2F13, IrBr3, ZrCl, YCl3, Cr2NiS4, CrCl3, Zn2(PS3)3, IrCl3, BiI, RhCl3, RhBr3, AlSbI6, Y2NCl3, ZrTe2Br5, Mn3As2, TaI2O, Sr(BiO2)2, TaCl4, Ag(TeMo)6, TaCl5, Ga2PdBr8, Ga2PdI8, GaCl3, CuCl2, NbCl4, K4GaAu8, BaMn3O6	63
Pnnm	Mg(C2N3)2, Cd(C2N3)2, KMn4(PO4)3, InS, Er(Fe2Ge)2, OsN2, Sm(Ni2P)2, GaTeCl, NaSn2Cl5, Be2Te7Cl6	10
Pnna	KInBr4, NdP2H9O10, GaBr2, Ag4Bi2O5, GeAsSe, TlFeBr4	6
C2/c	PS, S5N6, TbRb2F6, CuSe2O5, S3(NO)2, AgSbS2, PdSe2O5, AgCSN, SeS2(NO)2, YbK(WO4)2, Ag2PbO2, U2FeS5, KO2, Sb8(PbS5)3, PaCl5, Au2Se4O11, Ta2SnO7, PdBr2, HgBrO3, Tl2Sn2S3, B20H26O, Hg2IO, Pd(SeCl3)2, Ta2Zn3O8, V2Se9, PtI3, Ca3(AlAs2)2, TlAuCl4, S5N5Cl, Na2PrO3, AsBr5F6, C15S4, RbTe6, Cs2Au2Se3, CsWCl6, S(IO3)2, KBiO2, AsI5F6	38
Pnma	Rb2Te, Pu5Rh4, CrTe, Tm2S3, SiNi2, Li2S, HgO, Rb2Te, P4S3, P4S3, Sb2Se3, SnS, UF6, YF3, Sb2S3, TlGeS2, Tl3PSe4, Tl2SeO4, CaZrO3, CsSO2F, C2Se(SN)2, TbPt, SbOF, AsPO4, HgSeO3, ErF3, YbSiIr, ErGaCo, BaBOF3, SnHgP14, KBaPS4, Tl2TeS3, K2RuO4, Sn2Sb2S5, Rb5GeP3, KBaPSe4, EuRbPO4, Rb3AsSe4, KV2SbO8, USi, PbCN2, YbMnGe, YbCuS2, As2PbS4, MnCuAs, UFeSi, USiRh, MnCuP, UGeRh, YbGeRh, TbMnSi, PuSi, NpSi, UPdSe3, YbLaS3, FeS, YbMnSi, YbF3, DyMnSi, Yb2CaO4, UCrC2, SrRuO3, HgP14Pb, ScFeSi, HgCl2, SbCl3, CsI3, SbSeI, BiSeI, SbSI, Bi2Se3, PCl3, BiSCl, BiSBr, PNCl2, Sn2SI2, PBr2N, CsAg2I3, BiSI, GeBi2O5, Hg6S4IBr2Cl, LiBH, MgH2, K2H2IrCl5O, HS7N, NaBH4, KBH4, DyCoO3, SmCoO3, PrMnO3, EuMnO3, AuSeBr, PBr3, As4S3, CsBr3, Te2Br, Te2I, CsI2Br, Ti2PCl13, BaBrCl, PbBr2, RbDy2Cl7, NbXeF11, NdCl2, RbI3, TlI3, UTe5, NbBr5, Ga3Te3I, NbTe5Pd, Ta(NiTe)2, Al3Te3I, Nb3GeTe6, CBr3F, Ta2Te5Pd3, SiI3, TlAgSe, HSN, SmCl2,	123

	Sb4S5Cl2, SnBr2, CsPd2Cl5, Si3(Cl4O)2	
P6/mmm	TmSi2, B2Mo, USi2, LuSi2, SmFe5, HoSi2, UHg2, BaAg5, Cu2Te, ErSi2, ScSi2, Na2LiN, YNi4B, BaSi2, YbCo3B2, UFe3B2, B2W, YbB2, B2Au, PrAl2Ni3, TlPt, CaPd5, AgB2, Zr2Np, YbGa2, LaAl2Ag3, NpGa2, EuSi2, GdSi2, FeSn, CePd5, Ag7Te4, TaMn2O3	33
Amm2	Li3Ce5Ge4	1
Fmm2	SmS	1
I4/mmm	PbF4, YbC2, NdAl4, PrAl4, Ba2ZrS4, Sr(FeAs)2, Ca(FeP)2, Rb3TlF6, Rb3YF6, Cs3YF6, Ba(CuAs)2, Zr(NiP)2, PdO, Cs(MnP)2, LuPb2, Np(CrSi)2, Tl(FeS)2, Cs(FeSb)2, Mn3N2, Er2Mg, Tb2Mg, Pu(CrSi)2, Ba(MnP)2, Pu(FeSi)2, Np(CuGe)2, Ce2SbO2, In2CuO4, Eu(FeAs)2, Sr2UO4, Sr(MnGe)2, Np(CoGe)2, Ba(MnGe)2, HgI, HgCl, Ce2BiO2, HgBr, LaI2, Rb2CrCl4, K3AlH6, Sr2Co(BrO)2, Sr3Fe2Br2O5, AlPd5I2, NdI2, U3(SiC)2, Hf3Te2, Ca3SiBr2, KrF2, Mn2Au, BaZnBi2	49
P3m1	LiMnSe2, NaMnSe2, LiMnTe2, NaMnTe2, LiYMo3O8, LiScMo3O8, BiTeI, Nb3SBr7	8
$P2_1/c$	SN, SN, TiO2, As2S3, As2Se3, Sn3F8, As2O3, As2O3, Ag3O4, Rb2CO3, TbLi2F6, Hg3AsO4, ScOF, SnGeS3, AgCO2, Hg2NO4, TlHg(AsS2)3, Y2Si4CN6, YbOF, CuAgO2, K2Cd2O3, AgF2, CdSO3, Pd(AuF4)2, Mg(AuF4)2, HgSeO3, CaSO4, K2Pd(NO2)4, Zn3(AsO4)2, BS4N4F3, Ag2SeO3, Au3F8, USO6, ZnP2PbO7, UMoO6, WO2, SnMo5O8, PrMo5O8, SmMo5O8, NdMo5O8, CaMo5O8, Yb2S2O, K2TeBr6, LiAlCl4, IO2, XeF4, SnBr4, AlBr3, BiPO4, PAuCl4, Al2CuCl8, SiH4, AuBr3, BSBr, ReCl4O, Al2Hg3Cl8, SbN3Cl4, Se2O5, S4(BrN)3, CsSnCl3, Sn3BF9, Te3Cl2, TiBr4, AuCl3, MoS2Cl3, Te2PdCl10, NaNbCl6, AuSCl5, IrS3Cl11, LaSe2, PtI2, LiGaBr4, LiGaCl4, AuSeCl7, CS3N4, SiCl4, P3Se4I, SCl2O, Nd2GeO5, TlAs5S8, NaInI4, Si2NCl5, Zr2TeBr12, CsSbS6, Pt(SCl4)2, Nb2Te6I, OsO3F2, TaCoTe2, P2Se5, PSe, Br2O3, Pd(PbCl3)2, Pd(PbBr3)2, P4SeO6, S2N2Cl, H4SO5, Se2NCl5, SnCl2, InTeI, InTeBr, SICl7, I2(OF2)3, Ga2CuCl8, KGaI4, P4(SeO2)3, Pd(Se3Cl)2, Al2Te3, ClF, KAuI4, P2SeO3, CuSe2Br, KAuBr4, Hg3PO4, AgW3Br7, AsCl3O, SnCl4, Ir(Cl2F3)2, TiCl4, Hg2SeO3, LuPS4, AlI3, HgClO3, GaBr3, GaI3, KAl2Br7, GaPS4, NO, CuTe2Br, CuTe2I, CuSe2Cl, KAuI3, Tl2TeI6, NbI5, PNF2	134
$P2_1/m$	P2O3, As4S5, CeP5, ZrSe3, RbSO2F, AgCNO, P4S5, Te2Mo, SbAsO4, TaFeTe3, TaNi2Te3, USe3, TiS3, ZrS3, US3, Yb2TbPrS6, Yb2S3, Pr2I5, Pr2Br5, H(CO)2, SNCl, La3ReO8, Cs2HgI4, Tl3BSe3, Hf2CoP, Tl3BS3, TaSe3, Rb2ZnI4, PdSCl, La2I5, Li4V3O8	31
Imma	CaSiO3, RbHg2, LaGe2	3
Fddd	Cd2SiO4, Ag2SO4, Yb2Se3, Ag2SeO4, Hg2GeO4, Cu2SO4	6
$Pna2_1$	SO3, P4Se5, NaNdSiO4, NaYSiO4, ZnCl2, BiCl3, SbSBr, Nb6I11, H8S(NO2)2, PCl3O, C(Se2Br)2, Br2O, Hg5(SbI3)2, Re3Te4Cl5	14
Ibam	SiS2, KAuSe5, KAuS5, Na2CoS2, BeCl2, LiBiO2, Ta2Mn4Si5,	9

	CsPdCl3, BeBr2	
C2	VAg(PSe3)2, RbVP2S7, Nb2O5, CsVP2S7, Na4CO4	5
P$\bar{4}$m2	Ca(AuF6)2	1
P4/mnc	K2HfF6, Lu4CoB13, Tb4CoB13, Rb2TeI6, K2PtI6, Tl2TeBr6	6
P4/ncc	Tl4SnS3	1
I4/m	UF5, V5Te4, Yb5(LiGe)4, YbAu4, Ba2UCuO6, BiF5	6
Immm	K3Hg11, Ce3(CuGe)4, RuCl2O, OsCl2O, VBr2O	5
P4_2/mnm	GeO2, TeO2, Ba(TlHg)2, MgTa2O6, TaO2, NbCl3O, WI3O	7
Fmmm	Li2UO4, Cs2Pt3Se4, CrWO4	3
Pca2_1	RbPSe6, KPSe6, CS2NClO2, SeOF2, S8O, SBr2O, Se2Br3N, HS8N	8
Cm	KCN, Cu2HgI4, CuCO3, Ta3SBr7	4
Cc	K2Cu(PO3)4, ClO3	2
I222	BPS4, NaFeS2	2
P$\bar{3}$1c	GaAg(PSe3)2, ScAg(PS3)2, ScAg(PSe3)2, ErAg(PSe3)2, TmAg(PSe3)2, InAg(PSe3)2, InCu(PSe3)2, InAg(PS3)2, ZrFeCl6, HfFeCl6	10
P4$2_1$c	SNF, SeO3	2
I$\bar{4}$m2	TlAgTe2, TlFeS2, RbMnSe2, UGa3Ni	4
P4$2_1$m	BNF8, Ca2MgSi2O7, S7(N3O4)2	3
P$\bar{3}$1m	MoN, CoAg3(CN)6, TlAgSe2, NbHg3F6	4
I4mm	LiSi3Pd, Na5Co2S5	2
I$\bar{4}$c2	P2S2O3	1
I4	WBr4O, WCl4O	2
P$2_12_12_1$	CsB3O5, GeF2, KTaP4O13, Rb2S5, K2S5, K2Se5, SbSI, BCl, AsCl3, AsBr3, HgHOF, H2SeO4, H6CN3ClO, SbBr3, Si2H6O, SCl2, NaInBr4, SiCl2, BrNO3	19
Pba2	Au2Se2O7	1
P3_1	B2O3, RbNO3, Fe7S8, Hg3SO6	4
P$\bar{4}$2m	Cu2WS4	1
Cccm	Ba(InTe2)2	1
C222	HoSb2	1
P31m	Re2PbO8	1
P4/nmm	BaO, USiS, Ta2Se, NdTeF, TiSe, PbO, Li2NdSb2, LaMnGe, NaMnP, RbMnAs, FeSe, KMnP, U(CoP)2, Li2CeSb2, CaMnSi, GdO2, FeTe, UTe2, CeCoGe, Ce(SnIr)2, TbFeSi, RbMnP, SmCuSeF, Li2PrSb2, CuI, CuBr, BiClO, PbIF, BiIO, SrIF, CsLiBr2, NdBrO, BiBrO, InBi, CaHI, SrHI, CaHBr, SmFeAsO, LaFeAsO, TmIO, PuBrO, ThBrN, ThIN, PrIO, SmBrO, GdBrO, LaIO	47
Cmcm	ZrTe5, HfTe5, SnS, HgTe, CaSi, CaSn, Mn3As, MgGeO3, CuCN2, EuTl2Pd, LaTe2Se, MgSiO3, NdTe2Se, AgCNO, KAuS, RbAuO2, YbLaS3, BaF2, TaNiTe5, NbNiTe5, RbAuS, Tb2Si3, SmTe3, RbAuSe, KAuSe, YbSi, YbLaSe3, YbPrSe3, YbNdSe3, YbSmSe3, PuNi, GeS,	68

	AlPO4, Ga2O3, TaTe5Pt, CoAs, Fe3O4, TiFeO3, UMnSe3, DyGe, CaIrO3, InSb, NaAuO2, DyCoSn2, LiCeSn2, YbCeSe3, CeNi, InSb, Pb3SO6, UFeS3, InBr, CsCu2Cl3, UI3, TbCl3, Xe(OF)2, Te2AuCl, PuBr3, NdBr3, SmBr3, PuI3, LaI3, Mo2SBr2, Ta2NiS5, NbAlCl8, DyCl3, PrTe2Se, TbCoSn2, ErCoSn2	
$Pmc2_1$	CuAgS, TlP5, W(ClO)2	3
P4mm	BaTiO3, TiPbO3	2
$Pmn2_1$	TaTe4Ir, Te2W, Ag3PSe4	3
Cmce	CS2, NbCoTe2, Nd2CuO4, Pr2CuO4, Ta3I7, TbCsF5, WBr2, MoBr2	8
P31c	Re3(SBr)7	1
$P6_3$	Tl3PO4, Tl3AsO4, Cs2Mo3SeO12, PI3	4
$P3_{22}1$	SiO2, HgS	2
$P6_3/m$	Ca5P3O12F, Sr5P3O12F, PrB3, Ge3Sb2O9, Sr5P3ClO12, Sr5P3BrO12, BCl3, BI3, BBr3, Er3Ru2	10
Pma2	Hg2TeO3	1
$P\bar{6}2c$	W6CCl18	1
$P4_3212$	SiO2, Si2H6Se	2
$P\bar{6}2m$	Yb3Si5, NbFeB, K2HfF6, YbLiGe, YbAsPd, YbAgGe, HoMnGa, YbSnRh, Pu2Co, LaInPd, Dy3Mn3Ga2Si, YbMnGe, Cr2As, Co2As, Tb3Mn3Ga2Si	15
Pnc2	CuSe3Br	1
$P\bar{4}n2$	Nb(PS4)2	1
$P\bar{1}$	Cs(SbSe2)2, Au(OF3)2, Cu3(PO4)2, ScPS4, Tl2GeS3, KSO4, Tl2SiS3, Pr6O11, Cs(SbS2)2, TcS2, K(SbSe2)2, Rb(SbSe2)2, TiAgF6, YbNa(PS3)2, K2U3(TeO7)2, Ta2Tl4S11, Na2Cu3(GeO3)4, CaCrP2O7, BaCoP2O7, Rh(OF3)2, TlIn(PSe3)2, SiCl2O, AsS3(ClF2)3, TeI, IO3, MoNCl3, MoPCl9, Bi2TeI, SbI3Cl8, SiCl2O, KHCO3, HgPS3, UCl5, Re4Cl8O9, Nb(SeCl)2, WSCl4, TiPCl9, TaPCl10, SbI5F6, VNCl4, SbTeI, SbS8Cl3, Pd(SCl3)2, TiBr3, TlSbS2, TeAuCl7, Ta7(Te12I5)2, W(S4Cl3)2, Re(TeCl6)2, TaTe4I, Sb2S2O, Pt(SCl3)2, Te2OsCl12, Sr4Nb2O9, B2AsCl2, P4SO7, Te4MoBr, UBr5, Hf(Te2Cl3)2, Ta(TeBr3)2, Ta(TeCl3)2, PI2, P4SeO7, Pd(SeBr3)2, RuS3Cl8, RePCl9, Hf(Te4Cl3)2, NbTeCl9, Li4HN, LiMoS2, PAuS4, AlBiBr6	72
$P\bar{3}$	Tl2Sn(AsS3)2, Zn(ReO4)2, FePb2(CN)6, CrBr3, Ge2S3I2, AlSiTe3	6
P222	AlPS4	1
Aem2	SnS, LaS, PbS, CeS	4

Table S3 Crystal-system based distribution of predicted layered materials

Crystal system	Materials	#
Trigonal	B2O3, HgTe, PtO2, HgS, SnSe2, VSe2, PtS2, PtSe2, SnS2, ZrS2, CdTe, TaS2, ZrSe2, TiSe2, MoN, P2Pd3S8, RbSO3, Sr(MnAs)2, K2UF6, RbNO3, Sr(MnP)2, Nb2CS2, Tl2Sn(AsS3)2, CoAg3(CN)6, SiO2, Ca(MnP)2, GaAg(PSe3)2, TlPd2Se3, Cu3As, KAl(SO4)2, TlCdS2, HgO, NaTl3(SO3)2, Nd2O3, Li(TiSe2)3, ScAg(PS3)2, SF6, TlTe3Pt2, HgS, TlPt2S3, ZnTe, CsAu3S2, RbAu3Se2, CsAu3Se2, VS2, Yb(MnAs)2, NbSe2, LiMnSe2, NaMnSe2, Zn(ReO4)2, TaSe2, ZnNi3Sb2, Yb2SO2, LiMnTe2, NaMnTe2, Ca3N2, ScAg(PSe3)2, ErAg(PSe3)2, TmAg(PSe3)2, Yb2SeO2, PdF3, Ti3PO2, Na2GeF6, Na2SiF6, Na2TiF6, RbFe(MoO4)2, CoO, LiYMo3O8, LiScMo3O8, Li(TiS2)3, Eu(MnAs)2, Ce2O3, Gd2O3, CdInS2, InAg(PSe3)2, RhF3, FePb2(CN)6, Zn(InS2)2, Eu(MnP)2, Fe7S8, Na2SO4, InCu(PSe3)2, InAg(PS3)2, FeBr2, PbI2, BiTeI, WCl6, MgI2, UCl6, ZrP2(HO3)2, AlCl3, LiSnS2, CrBr3, TiBr2, BiSe, GeI2, Ge2S3I2, W2N, TlAgSe2, MnI2, TiCl2, ZrFeCl6, HfFeCl6, MnBr2, NbHg3F6, Sc2CCl2, Sc2NCl2, Nd(AlBr4)3, Nb3TeCl7, Nb3SBr7, Re2PbO8, Re3(SBr)7, FeAgTe2, TmI2, SiH, Sc2C, Nb3Cl8, CaI2, CoBr2, MgBr2, Hg3SO6, AlSiTe3, K2Hg7, Rb2Hg7	124
Orthorhombic	Rb2Te, ZrTe5, Pu5Rh4, TlF, NdTe3, CrTe, Tm2S3, SiNi2, Li2S, HfTe5, HgO, SnS, Rb2Te, CdAu, P4S3, P4S3, HgTe, CaSi, K3P11, SiS2, SmS, Sb2O3, Sb2Se3, SnS, CS2, Ag3Sb, UF6, SO3, YF3, PdSe2, P4Se5, CaSn, Mn3As, Sb2S3, CuAgTe2, MgGeO3, TlGeS2, PPdSe, CaCO3, SrCu2O3, KAuSe5, CuCN2, NaAsO2, KAuS5, EuTl2Pd, CsB3O5, Ag5SbS4, LaTe2Se, Tl3PSe4, MgSiO3, Ta2Pt3Se8, NdTe2Se, Tl2SeO4, Cd2SiO4, CaZrO3, TlAg3S2, CuAgS, CaSiO3, Li2PrO3, NbCoTe2, Ag2SO4, AgNO2, AgCNO, CsSO2F, C2Se(SN)2, Ba2Y(CuO2)4, KAuS, Yb2Se3, PPdS, TbPt, CaCu2O3, RbAuO2, GeF2, SbOF, Li2UO4, HoSb2, AsPO4, YbLaS3, TbBaF6, BaF2, Na2CoS2, SnS, CuAgS, TaNiTe5, NbNiTe5, RbAuS, Tb2Si3, HgSeO3, ErF3, SmTe3, RbAuSe, RbHg2, KAuSe, DyBC, SiP2, Mg(C2N3)2, YbSiIr, YbSi, GeO2, Al4CN3O, K3Hg11, ErGaCo, Ag2SeO4, Cd(C2N3)2, YbLaSe3, YbPrSe3, YbNdSe3, YbSmSe3, PrTe3, TiCu3, CdTe, PuNi, GeS, NaMgF3, AlPO4, La3Ni2Sn7, Ga2O3, ZrGeTe4, RbNO3, BaBOF3, PdS2, NaNdSiO4, KTaP4O13, Hg2GeO4, Cs2Pt3Se4, Hf(PS3)2, TaTe5Pt, CoAs, Nd2CuO4, SiCuO3, Pr2CuO4, SnHgP14, Rb2S5, KBaPS4, K2S5, Tl2TeS3, TaTe4Ir, GeAs2, K2RuO4, Sn2Sb2S5, NaYSiO4, RbPSe6, Rb5GeP3, Ta2Pd3Se8, KBaPSe4, EuRbPO4, Rb3AsSe4, K2Se5, KPSe6, Fe3O4, CrWO4, TiFeO3, KV2SbO8, KMn4(PO4)3, Sm4Mo4O11, USi, PbCN2, InSb, LaGe2, InS, TiPbO3, TlSbSe2, YbMnGe, YbCuS2, UMnSe3, As2PbS4, Li3Ce5Ge4, MnCuAs, UFeSi, DyGe, USiRh, CaIrO3, MnCuP, LaS, InSb, UGeRh, PrMnSi, NaAuO2, Er(Fe2Ge)2, YbGeRh,	428

	Ba2Y(CuO2)4, PbS, CeS, DyCoSn2, TbMnSi, PuSi, LiCeSn2, USnPd, YbCeSe3, NpSi, Yb(NdS2)2, Fe4P, UPdSe3, YbLaS3, Ba(InTe2)2, CeNi, InSb, OsN2, FeS, YbMnSi, SrAlBO4, Sm(Ni2P)2, YbF3, DyMnSi, Yb2CaO4, UCrC2, SrRuO3, TiSi, HgP14Pb, Pb3SO6, Ce3(CuGe)4, CsNO3, UFeS3, Te2W, ScFeSi, HgCl2, InBr, SbCl3, PBr5, CsI3, ZnCl2, BiCl3, KInBr4, AgClO2, C(ClF)2, SbSBr, TiClO, SbSeI, CrSBr, TiBrO, SbSI, BiSeI, SbSI, ZrIN, CsCu2Cl3, CCl3F, CS2NClO2, Bi2Se3, HgI2, ClO2, Nb6I11, PCl3, Ta3I7, UI3, BCl, TiI3, BeCl2, AsCl3, HgBr2, TbCl3, RuBr3, BrF3, MoBr3, BCl2, AsBr3, BiSCl, BiSBr, PNCl2, Sn2SI2, PBr2N, CsAg2I3, BiSI, GeBi2O5, TeSeS(NCl)2, Hg6S4IBr2Cl, LiBH, MgH2, K2H2IrCl5O, HgHOF, H2SeO4, H6CN3ClO, NdP2H9O10, HS7N, H8S(NO2)2, NaBH4, KBH4, DyCoO3, SmCoO3, PrMnO3, EuMnO3, AuSeBr, As2SO6, LuSBr, Au2O3, PBr3, PCl3O, ZrTi2O, TmCl2, Xe(OF)2, SeOF2, SbBr3, TlP5, KAuO2, GaTeCl, CN, AlPS4, S8O, Tc2O7, Te2AuCl, CNCl, As4S3, ErSCl, IBr, CsBr3, Te2Br, Te2I, PuPt4, CsI2Br, InClO, BPS4, TcCl4, CsReBr4, TiIN, TiBrN, TiNCl, AlClO, Ti2PCl13, Si2H6O, PuBr3, NdBr3, SmBr3, PuI3, LaI3, CBrN, Mo2SBr2, AlP2I9, BaBrCl, RbPaF6, PbBr2, RbDy2Cl7, Au2Se2O7, SCl, SBr, NaFeS2, SCl2, C(Se2Br)2, NbXeF11, LiBiO2, OsBr4, Ta2NiS5, NdCl2, RbI3, TlI3, NbAlCl8, GeH3Cl, GaBr2, S(ClO)2, SBr2O, B2PCl2, DyCl3, TbCsF5, ErSeI, Br2O, Cu2SO4, UTe5, Ta4SiTe4, NaInBr4, NbBr5, Ga3Te3I, NbTe5Pd, Ta(NiTe)2, Al3Te3I, CuSe3Br, Nb3GeTe6, CBr3F, Se2NCl3, Mn5As4, Hg5(SbI3)2, DySBr, DySI, Ta2Te5Pd3, NaSn2Cl5, Ag8S, Ag4Bi2O5, CaThBr6, SrThBr6, Hg3TeBr4, Hg3TeCl4, SiI3, Re3Te4Cl5, Se2Br3N, RuCl2O, OsCl2O, SiCl2, TlAgSe, GeAsSe, Si2H6S, S3N2Cl, HSN, TlFeBr4, CuMo3I7, SmCl2, HS8N, WBr2, Hg2TeO3, BrNO3, MoBr2, Sb4S5Cl2, Ta2Mn4Si5, Ho14Ge23, GaP2I9, HgCN2, SnBr2, CsPdCl3, Tl2Au4S3, SbClF10, LiNb6Cl19, CsPd2Cl5, AuSO4, DyBr2, Be2Te7Cl6, AsCl5, RbNb4Cl11, Zr(Se2Cl3)2, W2CCl8, PrTe2Se, BeBr2, Bi2CO5, Si3(Cl4O)2, RbAsO2, Ag3PSe4, TbCoSn2, ErCoSn2, Zr(TeCl)6, CrBrO, VBr2O, VBrO, W(ClO)2	
Monoclinic	SN, SN, P2O3, TiO2, As4S5, CeP5, PS, As2S3, P4S5, As2Se3, S5N6, Sn3F8, As2O3, As2O3, Ag3O4, ZrSe3, SiAs, HgO2, AuSe, TbRb2F6, CuSe2O5, ZnPS3, S3(NO)2, AgSbS2, Rb2CO3, TbLi2F6, Hg(CO2)2, Hg3AsO4, YbTaO4, PdSe2O5, ScOF, SnGeS3, AgCO2, CdPS3, AgPS3, LuTaO4, Hg2NO4, FePS3, AgCSN, TlHg(AsS2)3, Y2Si4CN6, RbSO2F, VAg(PS3)2, VAg(PSe3)2, K2Cu(PO3)4, RbNb2PS10, AgCNO, YbOF, CuAgO2, P4S5, Te2Mo, Hg2SO4, Hg2SeO4, K2Cd2O3, AgF2, LiSO3F, SbAsO4, NiC2(SN)2, Na5ReO6, Ta2PdS6, Ta2PdSe6, CdSO3, Pd(AuF4)2, MnPS3, ZrTiTe4, Mg(AuF4)2, SeS2(NO)2, TaFeTe3, RbVP2S7, CdHgO2, Al2Te5, TaNi2Te3, USe3, GeAs, TlFeSe2, TiS3, ZrS3, Nb2O5, HgSeO3, Yb2Ge2Ir, CsVP2S7, CaSO4,	313

	YbKSiS4, US3, Na4CO4, Yb2TbPrS6, K2Pd(NO2)4, Ta2PtSe7, Zn3(AsO4)2, KSb5S8, Yb2S3, BS4N4F3, Ag2SeO3, Au3F8, USO6, ZnP2PbO7, UMoO6, WO2, SnMo5O8, PrMo5O8, SmMo5O8, NdMo5O8, CaMo5O8, YbK(WO4)2, RbAg3S2, KCN, Ag2PbO2, U2FeS5, KO2, InSe, Yb2S2O, Tb2CoGe2, Sb8(PbS5)3, NaN3, MoCl3, Pr2I5, ClO3, TeRhCl, K2TeBr6, LiAlCl4, Pb5(SI3)2, Cs2Pd(IBr2)2, Pr2Br5, IO2, XeF4, SnBr4, CuBr2, AlBr3, NbCl5, BiPO4, Cu2HgI4, PAuCl4, GaSeBr7, Al2CuCl8, Y6C2I7, NbSeBr3, H(CO)2, SiH4, AlCl3, UBr4, GdBr3, AuBr3, CrI2, BSBr, CuCO3, ReCl4O, ReCl4, Al2Hg3Cl8, SbN3Cl4, Se2O5, Nb(SCl)2, S4(BrN)3, TlHg5Cl11, Na5Zr2F13, CsSnCl3, IrBr3, Sn3BF9, PaCl5, ZrCl, Au2Se4O11, YCl3, Cr2NiS4, Ta2SnO7, Te3Cl2, CrCl3, TiBr4, AuCl3, Zn2(PS3)3, IrCl3, S3(NCl)2, BiI, As2S2O9, RhCl3, PdBr2, MoS2Cl3, RhBr3, AlICl6, SNCl, HgBrO3, Tl2Sn2S3, NbTeBr3, B20H26O, AlSbI6, La3ReO8, Hg2IO, Pd(SeCl3)2, Te2PdCl10, NaNbCl6, AuSCl5, IrS3Cl11, LaSe2, Ta2Zn3O8, V2Se9, PtI3, Ca3(AlAs2)2, PtI2, LiGaBr4, LiGaCl4, Al2CdCl8, TlAuCl4, AuSeCl7, S5N5Cl, CS3N4, SiCl4, P3Se4I, SCl2O, Nd2GeO5, Cs2HgI4, Na2PrO3, TlAs5S8, Tl3BSe3, NaInI4, Y2NCl3, Si2NCl5, K3AuSe13, AsBr5F6, ZrTe2Br5, Zr2TeBr12, CsSbS6, C15S4, Pt(SCl4)2, Nb2Te6I, TeCF2, OsO3F2, TaCoTe2, P2Se5, RbTe6, PSe, Mn3As2, Br2O3, Pd(PbCl3)2, Pd(PbBr3)2, ZrSnCl6, P4SeO6, TaI2O, Sr(BiO2)2, S2N2Cl, H4SO5, Se2NCl5, Hf2CoP, SnCl2, Cs2Au2Se3, InTeI, InTeBr, SICl7, I2(OF2)3, Ga2CuCl8, Tl3BS3, K2U2O7, KGaI4, AlTeI7, AlSeBr7, HfCl4, TaCl4, P4(SeO2)3, Pd(Se3Cl)2, Al2Te3, ClF, KAuI4, GaTeI7, P2SeO3, CuSe2Br, Ag(TeMo)6, TaSe3, KAuBr4, Hg3PO4, AgW3Br7, TaCl5, AsCl3O, SnCl4, Ir(Cl2F3)2, Na3Li3N2, Rb2ZnI4, Eu(AlCl4)2, Ba(AlCl4)2, C2S9N2, TiCl4, PdSCl, La2I5, Hg2SeO3, LuPS4, AlI3, HgClO3, Ga2PdBr8, Ga2PdI8, GaCl3, GaBr3, GaI3, CsWCl6, KAl2Br7, GaPS4, S(IO3)2, KBiO2, CuCl2, NO, Ta3SBr7, AsI5F6, CuTe2Br, CuTe2I, CuSe2Cl, NbCl4, KAuI3, Ag2S, As8S9, Tl2TeI6, K4GaAu8, NbI5, PNF2, AgTe2Au, BaMn3O6, Hg2Mo2O7, Li4V3O8, Ba2V3O9	
Tetragonal	PbF4, YbGa, GeO2, SeO2, PuGe2, YbC2, NdSb, PrSb, UF5, MnNi, NdAl4, UF5, LaAg, NdGe2, TeO2, PrAl4, TlSn, S2N, CaSiO3, CaSiO3, Ba(TlHg)2, P2S2O3, LiYF4, Ba2ZrS4, Sr(FeAs)2, Yb2CdPd2, BNF8, SNF, Ca(FeP)2, Ca2MgSi2O7, SrSi, SiO2, KHgF3, BaO, GeS2, Rb3TlF6, Rb3YF6, Cs3YF6, USiS, YbAgS2, YbLiO2, K2HfF6, Ta2Se, Cu2WS4, Ba(CuAs)2, Cu2GeO4, Tl4SnS3, MgTa2O6, NbP, V5Te4, TlAgTe2, GeSe2, CsAgC2, ThGe2, LaSb, YGe2, Zr(NiP)2, RbAuC2, Yb2AlSi2, Sc2AlSi2, PdO, Yb5(LiGe)4, Cs(MnP)2, YbAu4, TiCdHg2, TiCu, LuPb2, PrGe2, Np(CrSi)2, Tl(FeS)2, Cs(FeSb)2, YbLiF4, CoHgC4(SeN)4, YbPO4, TaP, NdTeF, SmGe2, YSi2, DySi2, TlFeS2, Al2Cu, Mn3N2, BaTiO3, TiSe, Yb2MgSi2, Er2Mg, Tb2Mg, UOF4,	220

	Pu(CrSi)2, Ba(MnP)2, PbO, PuSb, TbB4, BaTiO3, Np2InNi2, Li2NdSb2, FeSe, Pu3Al, Pu(FeSi)2, LaMnGe, NaMnP, RbMnAs, Np(CuGe)2, FeSe, Ce2SbO2, KMnP, TiPbO3, In2CuO4, LaAs, Ta2InCuTe4, Lu4CoB13, GaCo2Ni, Th3Si2, U(CoP)2, LiSi3Pd, Li2CeSb2, Eu(FeAs)2, TaO2, Sr2UO4, CeGe2, CaMnSi, Sr(MnGe)2, GdO2, Yb2InPd2, FeTe, UTe2, CeCoGe, Ce(SnIr)2, TbFeSi, RbMnP, PuIn, Np(CoGe)2, Ba2UCuO6, SmCuSeF, Ba(MnGe)2, Li2PrSb2, Tb4CoB13, Ba2Ca2Cu3HgO8, UFeC2, U2Ni2Sn, HgI, CuI, HgCl, CuBr, BiClO, PbIF, BiIO, Ce2BiO2, SrIF, CsLiBr2, NdBrO, BiBrO, HgBr, HgI2, LaI2, PCl5, InBi, SbCl4F, Sr2InI5, Rb2CrCl4, Cs4BiSbCl12, K3AlH6, TcH4NO4, CaHI, SrHI, CaHBr, Sr2Co(BrO)2, Sr3Fe2Br2O5, SmFeAsO, LaFeAsO, Mg(SbO2)2, AlPd5I2, TmIO, SeO3, AuI, CCl2O, BiF5, NbCl3O, TaCl4F, PuBrO, ThBrN, ThIN, Rb2TeI6, Nb(PS4)2, Ca(AuF6)2, K2PtI6, S7(N3O4)2, RbBrF4, CsBr2F, NdI2, U3(SiC)2, Hf3Te2, PrIO, Cs3CoBr5, Na5Co2S5, Si2H6Se, SmBrO, GaCuI4, Hf(Se2Cl3)2, Re2(PCl8)3, RbMnSe2, GdBrO, Bi2Se2Cl7, Ca3SiBr2, NaLi5N2, KrF2, CsH3O2, TiAgHg2, Zr3Ag, Mn2Au, BaZnBi2, MgPt3, LaIO, Tl2TeBr6, InPd3, UGa3Ni, WI3O, WBr4O, WCl4O, SrCuO2	
Hexagonal	WS2, Yb3Si5, Fe5Si3, CrS, Te2Mo, TmSi2, GaN, B2Mo, USi2, BN, LuSi2, Na3As, VS, ZrTe, SmFe5, GaSe, MoSe2, CrSb, Co2Ge, HoSi2, UHg2, BaAg5, WSe2, Cu2Te, GaSe, TaS2, ErSi2, GaS, MoS2, ScSi2, Ti2SnC, Zr2AlC, Zr2SnC, Zr2AlN, Hf2SnC, Ti2AlN, Nb2SnC, Tl3PO4, Ca5P3O12F, Sr5P3O12F, Mn2Sb, YN, Na2LiN, TaGe2, YNi4B, BaSi2, NbFeB, AlSn, U2AlCu3, ReB3, PtO2, TaSe2, YAlO3, BN, V5P3N, YbSiCu, LiSmAlF6, Na2S, K2HfF6, YbLiGe, YbAsPd, ErCuGe, AlAgO2, YbAgGe, YbPPt, BaLiSi, SnS2, GaTe, RuC, NbS2, YbCo3B2, UFe3B2, B2W, YbB2, MnSe, Cr2GaN, SrLiP, BaLiP, BaLiAs, TaS, SmSe, TbSe, YbSe, TmTe, AuSe, B2Au, FeTe, PrAl2Ni3, U2Al3Os, ZrAlPt2, AlAs, BaAgSb, SrAgSb, YAl3, Mg3Au, TlNi, TlPt, Yb5Ge3, YbCuSb, K3N, SrSbAu, CaPd5, HoMnGa, YbSnRh, AgB2, FeN, YbCsSe2, Zr2Np, KAg2, ThMn2, Sr2Si, ZrN, PtN, AlN, YbGa2, Cs2Pt, K5CuAs2, Tl3AsO4, PrB3, LaAl2Ag3, Ge3Sb2O9, Ge3Mo5C, BaSiO3, Mg2Mo3O8, Zn2Mo3O8, NpGa2, EuSi2, Zr2InC, Pu2Co, GdSi2, LaInPd, Sc2InC, Ti2InC, CaHgPb, Dy3Mn3Ga2Si, YbMnGe, ZrNiP, FeSn, PtPb, Cr2As, Ta2InC, Co2As, CePd5, Tb3Mn3Ga2Si, RuCl3, NiBi, BiRh, ZrCl3, MnBi, RuBr3, BiPt, Sr5P3ClO12, Sr5P3BrO12, SbCl5, BCl3, BI3, TiI3, BBr3, ZrBr3, ZrI3, SrH2, MnH, CaH2, Cs2Mo3SeO12, Sc2Co3Si, PI3, CdBr2, Ag7Te4, CdI2, RbTiCl3, CsTiCl3, Sm4Cl6O, Ca2IN, LaBr2, BiTeCl, MnWN2, Ta3SeI7, Ta3TeI7, AuCN, LaTlO3, RbVBr3, Rb3Mo2Cl9, Rb3Mo2Br9, Si3Ir, Nb3TeI7, W6CCl18, BaAgBi, LuMn5, Er3Ru2, TaMn2O3, Na2MnO4	191
Triclinic	Cs(SbSe2)2, Au(OF3)2, Cu3(PO4)2, Zn(GaS2)2, ScPS4, Tl2GeS3,	80

	KSO4, Tl2SiS3, Tl3SbS4, Sr2Cu2O3, Pr6O11, Cs(SbS2)2, TcS2, K(SbSe2)2, Rb(SbSe2)2, TiAgF6, YbNa(PS3)2, K2U3(TeO7)2, KAsSe2, Zn(GaSe2)2, Ta2Tl4S11, Na2Cu3(GeO3)4, CaCrP2O7, Na2ZrCo(P2O7)2, BaCoP2O7, Rh(OF3)2, TlIn(PSe3)2, SiCl2O, AsS3(ClF2)3, TeI, IO3, MoNCl3, MoPCl9, Bi2TeI, SbI3Cl8, SiCl2O, KHCO3, MnO2, HgPS3, UCl5, Re4Cl8O9, Nb(SeCl)2, WSCl4, TiPCl9, TaPCl10, SbI5F6, VNCl4, SbTeI, SbS8Cl3, Pd(SCl3)2, FeSeCl7, TiBr3, TlSbS2, TeAuCl7, Ta7(Te12I5)2, W(S4Cl3)2, Re(TeCl6)2, TaTe4I, Sb2S2O, Pt(SCl3)2, Te2OsCl12, Sr4Nb2O9, B2AsCl2, P4SO7, Te4MoBr, UBr5, Hf(Te2Cl3)2, Ta(TeBr3)2, Ta(TeCl3)2, PI2, P4SeO7, Pd(SeBr3)2, RuS3Cl8, RePCl9, Hf(Te4Cl3)2, NbTeCl9, Li4HN, LiMoS2, PAuS4, AlBiBr6	

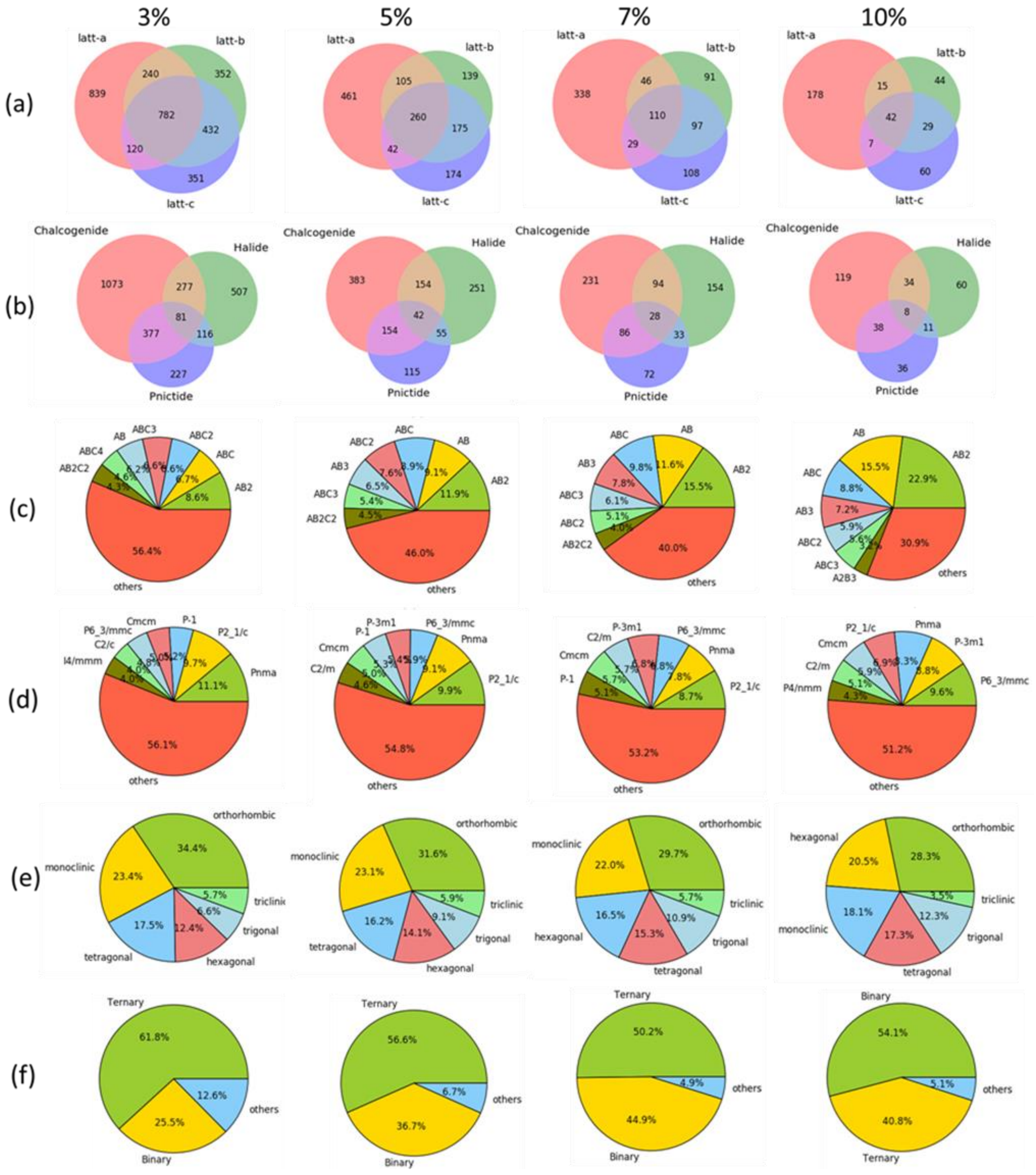

Figure S1 Classification of the materials identified as possible 2D as the selection criterion becomes more stringent (relative error in lattice constant equal or larger than 3%, 5%, 7% and 10% in the first, second, third and fourth column, respectively. Rows (a)-(f) contain the relative error in lattice constants (a), chemical composition (b), crystal prototype (c), crystal space group (d), structure (e) and number of distinct chemical constituents (f) (same labeling as in Fig. 1in the main text).

Table S4 Distribution of materials with relative error in lattice constant equal or larger than δ, [illegible] δ as defined in Eq. 1. Materials counted in column 2, 3, 4 have relative error equal or larger than δ in only one of the lattice constants (*a* or *b* or *c*), while column 5, 6, 7 sum up all the materials that have such an error in lattice constant *a*, *b* or *c*, respectively.

δ=relative error in lattice constant	% of materials with ONLY error in *a*	% of materials with ONLY error in *b*	% of materials with ONLY error in *c*	% of materials with error in *a*	% of materials with error in *b*	% of materials with error in *c*
3%	0.27	0.11	0.11	0.64	0.58	0.54
5%	0.34	0.10	0.13	0.64	0.50	0.48
7%	0.41	0.11	0.13	0.64	0.42	0.42
10%	0.47	0.12	0.16	0.65	0.35	0.37

Table S5 Distribution of chemical composition among the materials that our screening criterion suggests as possible 2D for various values of δ as defined in Eq. 1.

δ =relative error in lattice constant	% of Chalcogenides only materials	% of Halides only materials	% of Pnictides only materials	% of Chalcogenides (total)	% of Halides (Total)	% of Pnictides (Total)
3%	0.40	0.19	0.09	0.68	0.37	0.30
5%	0.33	0.22	0.10	0.64	0.44	0.32
7%	0.33	0.22	0.10	0.63	0.44	0.31
10%	0.39	0.20	0.12	0.65	0.37	0.30

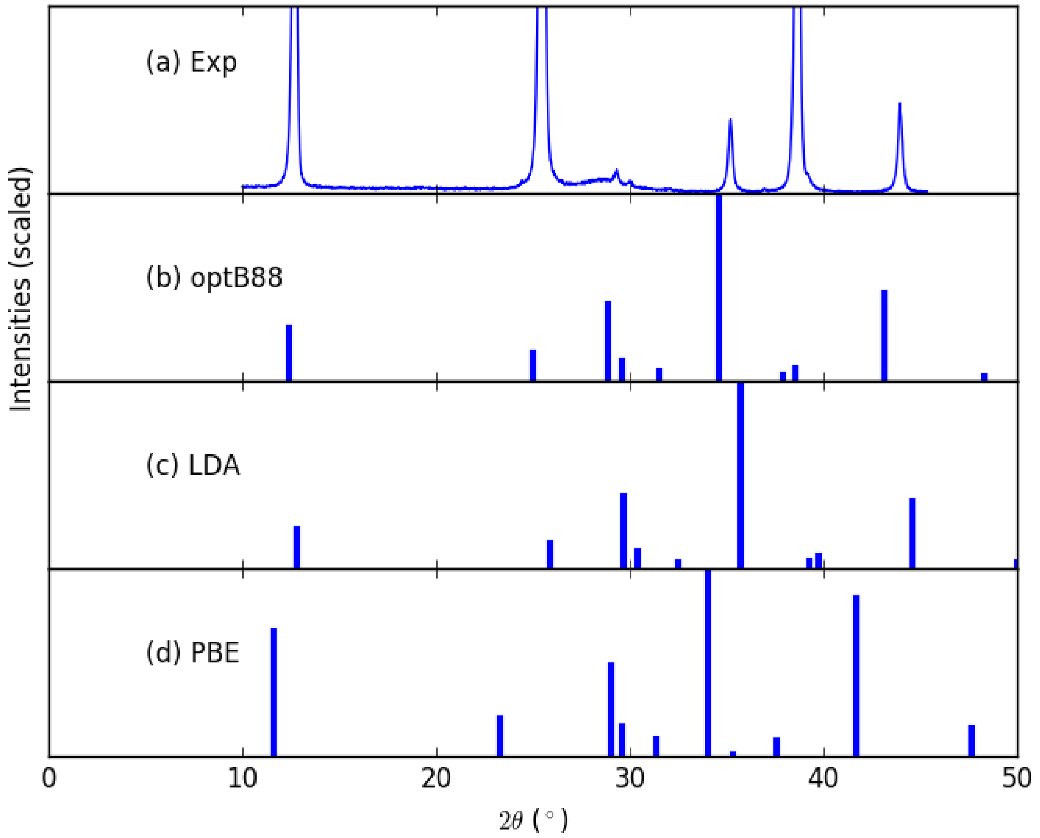

Fig. S2 showing XRD pattern of 2H $MoTe_2$ structure using (a) Experiment, (b) optB88 functional, (c) LDA functional, (d) PBE for bulk material. Due to good lattice constants match, LDA and optB88 gives reasonable XRD pattern compared to experimental XRD data.

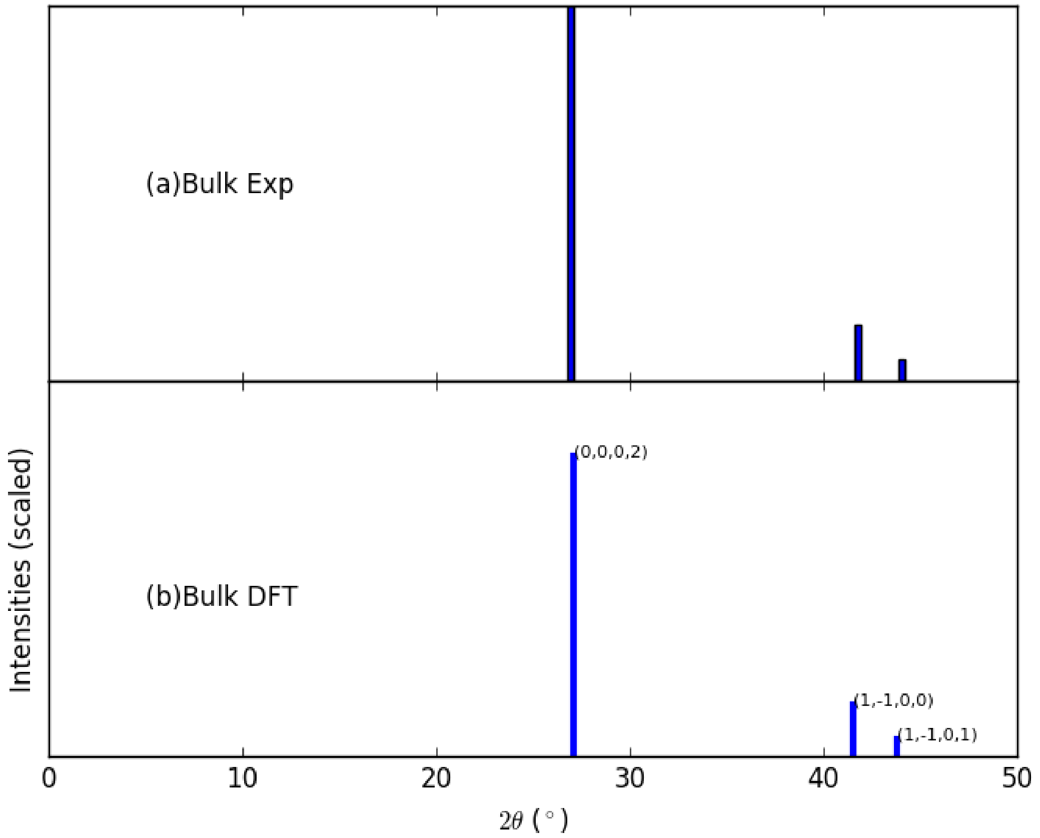
(a)Bulk Exp
(b)Bulk DFT
(0,0,0,2)
(1,-1,0,0)
(1,-1,0,1)
Intensities (scaled)
0
10
20
30
40
50
2θ (°)

Fig. S3a

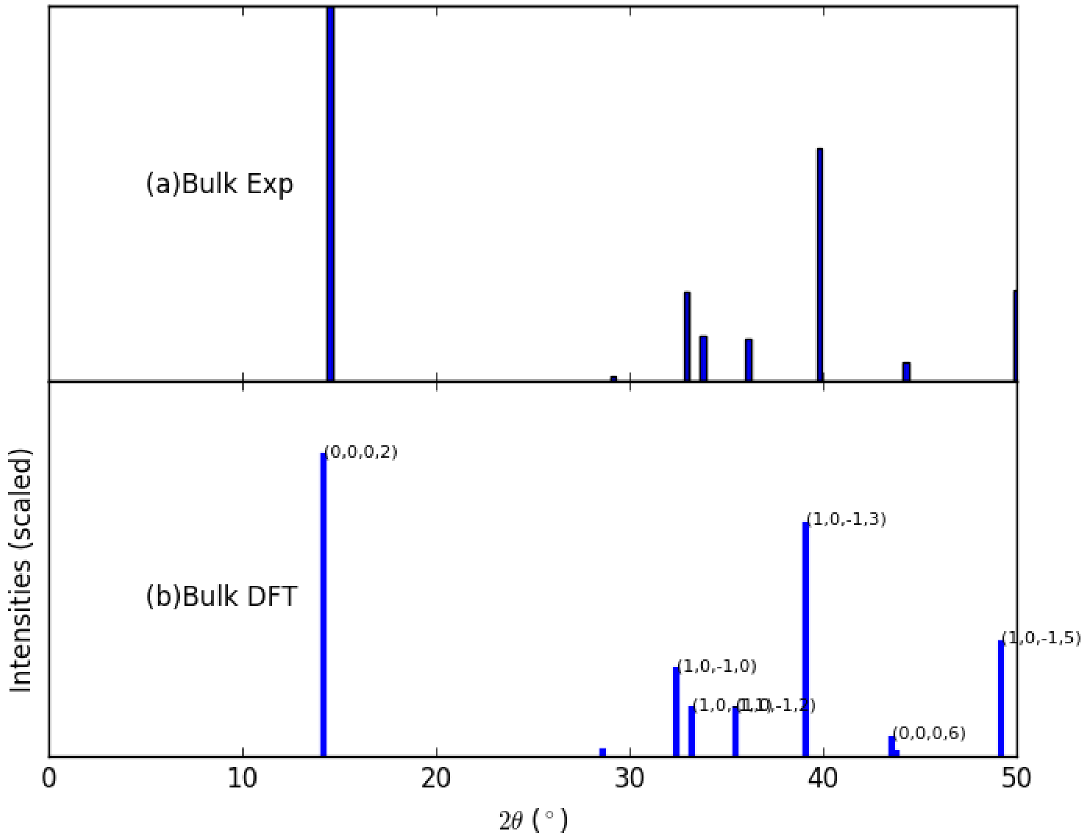
(a)Bulk Exp
(b)Bulk DFT
Intensities (scaled)
(0,0,0,2)
(1,0,-1,3)
(1,0,-1,5)
(1,0,-1,0)
(0,0,0,6)
0
10
20
30
40
50
2θ (°)

Fig. S3b

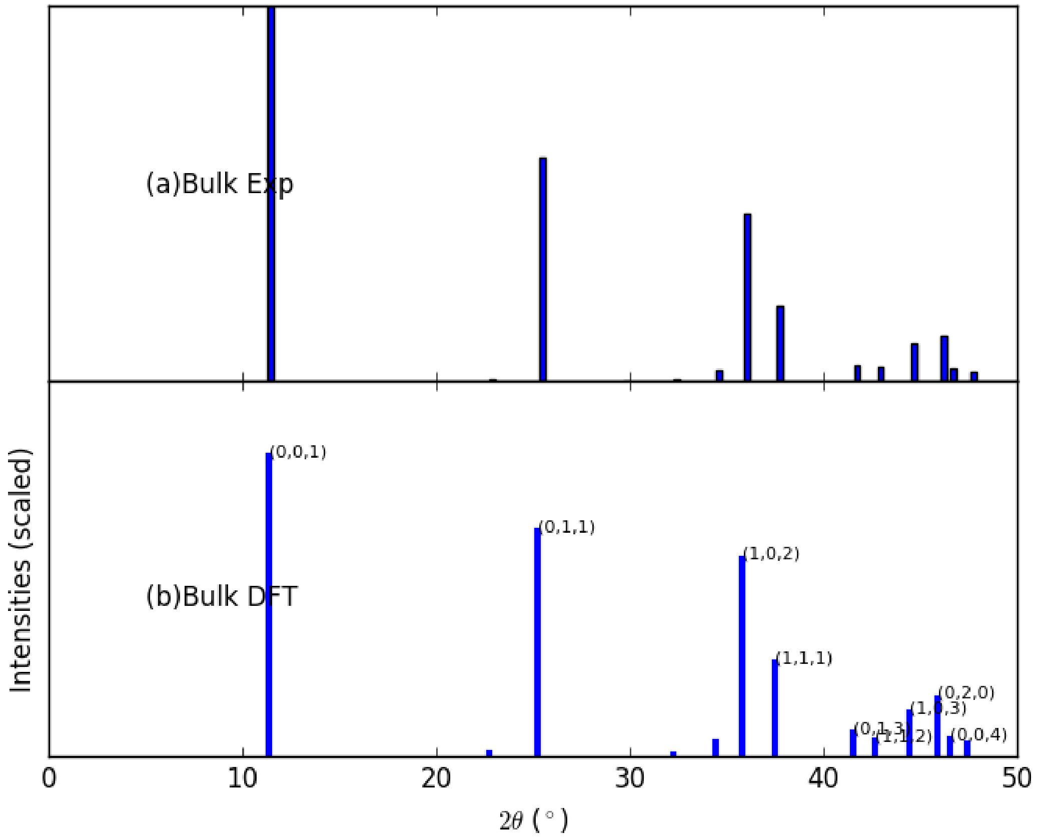
(a)Bulk Exp
(b)Bulk DFT
(0,0,1)
(0,1,1)
(1,0,2)
(1,1,1)
(0,1,3)
(1,1,2)
(1,0,3)
(0,2,0)
(0,0,4)
Intensities (scaled)
0
10
20
30
40
50
2θ (°)

Fig. S3c

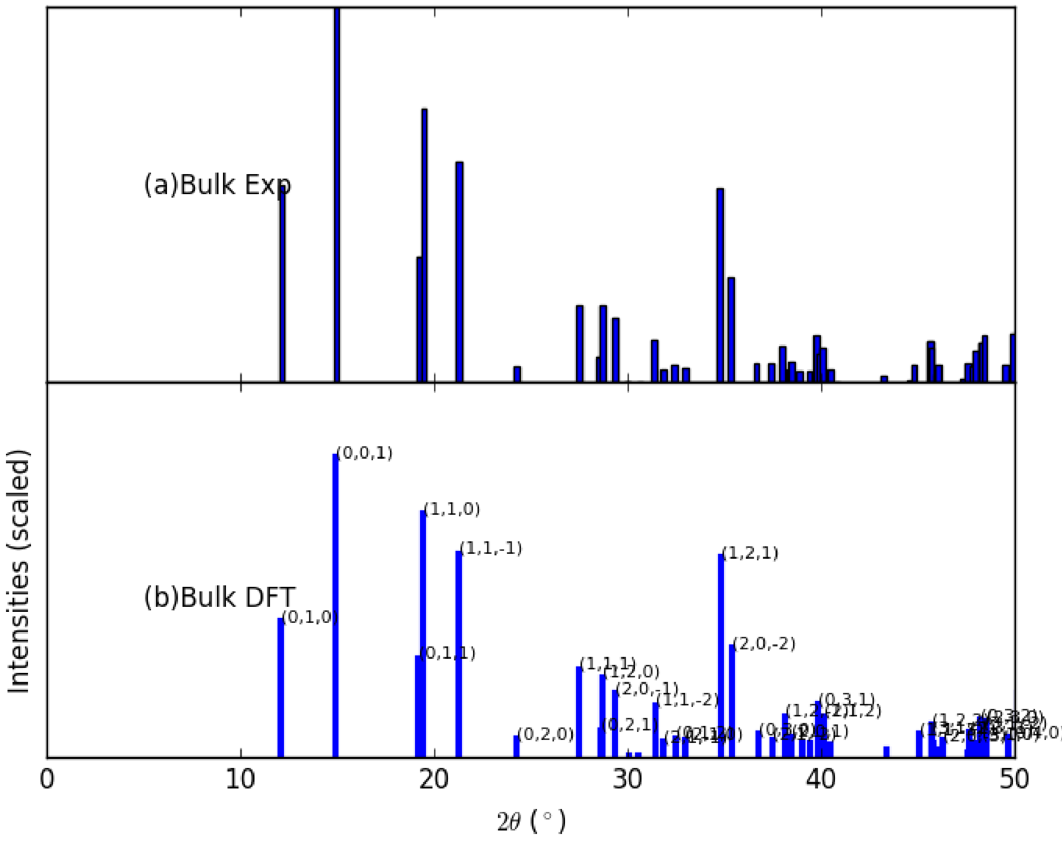

(a)Bulk Exp
(b)Bulk DFT
Intensities (scaled)
(0,0,1)
(1,1,0)
(1,1,-1)
(1,2,1)
(0,1,0)
(0,1,1)
(2,0,-2)
(1,1,1)
(1,2,0)
(2,0,-1)
(1,1,-2)
(0,2,0)
(0,2,1)
(0,3,1)
0
10
20
30
40
50
2θ (°)

Fig. S3d

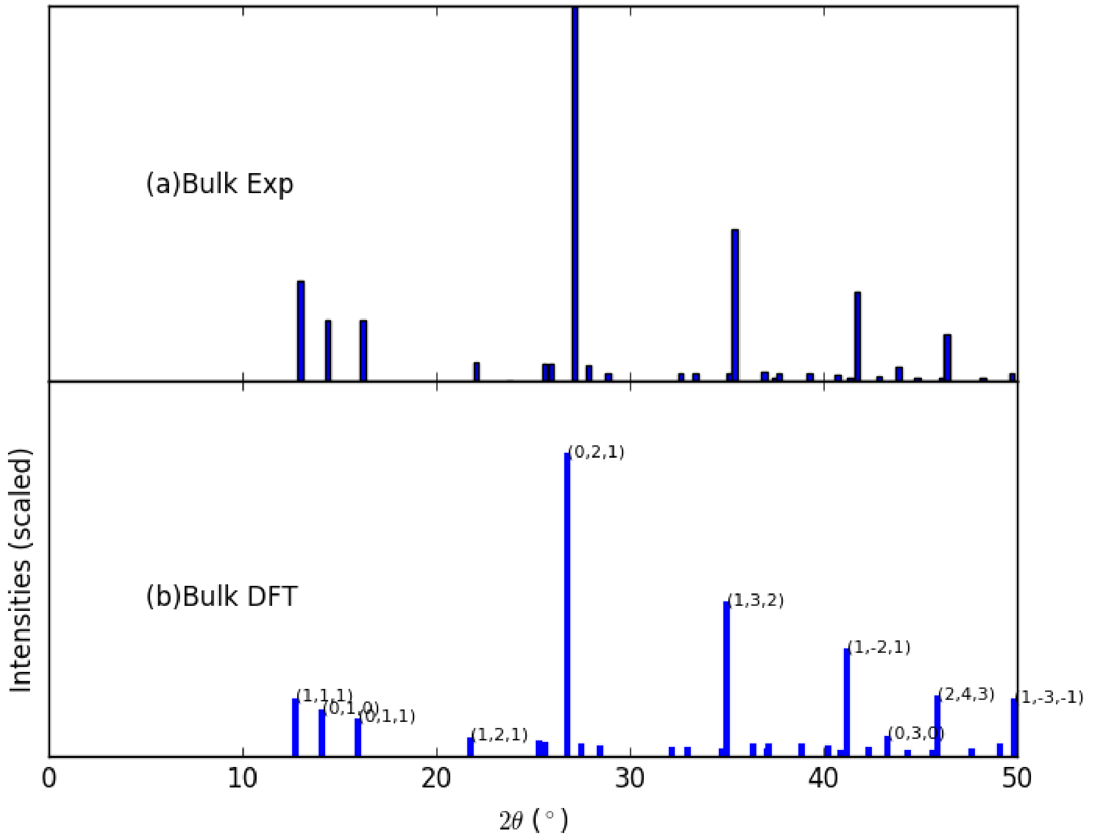

Fig. S3e

Fig. S3 X-ray diffraction pattern for a) BN ($P6_3/mmc$), b) $MoS2$ ($P6_3/mmc$), c) TiNCl (Pmmn), d) $HfCl_4$ (P2/c), e) BiI_3 (R-3) from experiments and optB88 based DFT. Experimental data were obtained from MDI-JADE 6.5 software.

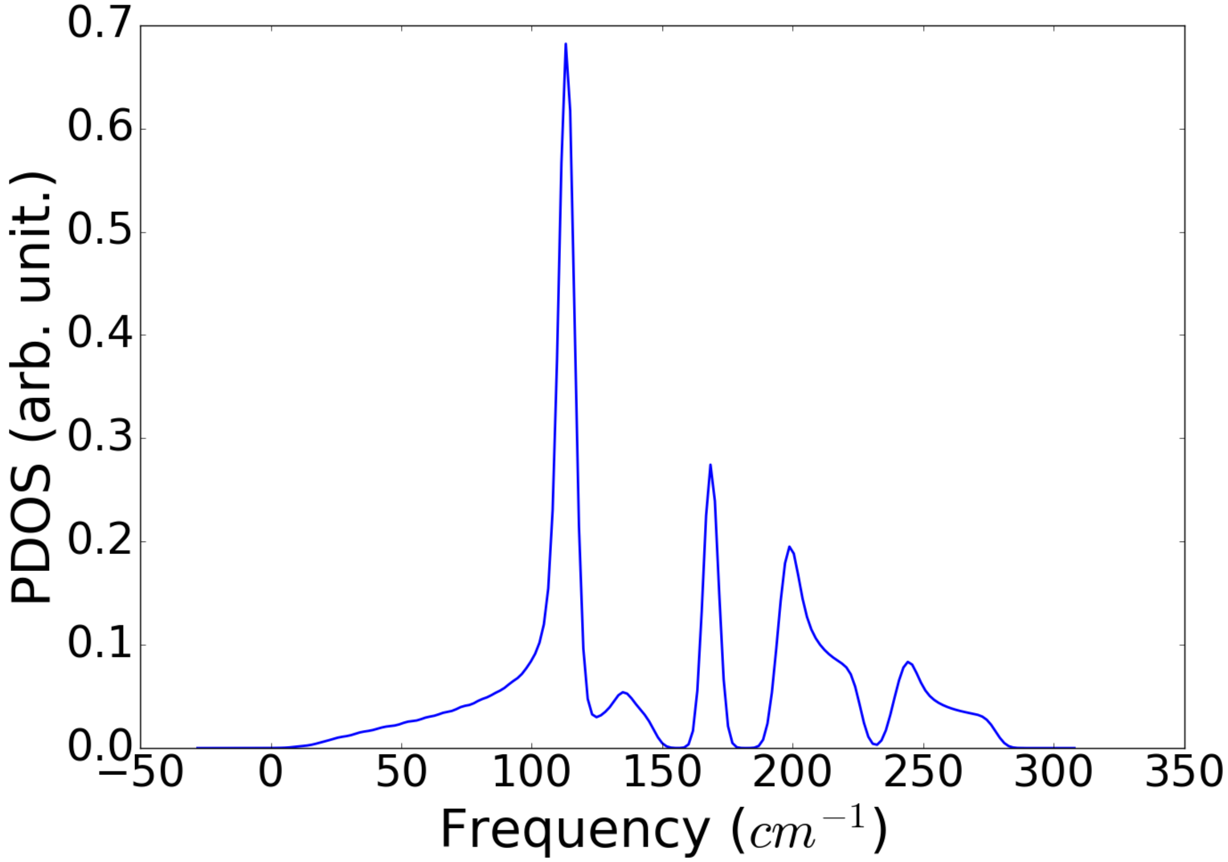

Fig S4: Phonon density states of $MoTe_2$ using optB88 functional.

Table S6: Bulk and layers properties of 2D materials. E_f stands for exfoliation energy, SG stands for space group, SCF-Bulk-Gap and SCF-1L-Gap are the bandgap obtained from SCF run for bulk materials, and layered material respectively. BRIL-Bulk-Gap and BRIL-1L-Gap are the bandgaps obtained with special high-symmetric points along Brillion zone for bulk and layered materials respectively. Among these two values, one with minimum of these two should be considered as optB88 based bandgap of materials. Materials-project ID for the structures are denoted by MPID from which bulk and single-layer calculation were carried out with optB88 functional. Absolute ratio is based on ICSD and MP-PBE c-lattice constants. The database is sorted based on increasing exfoliation energy. Data not available right now are marked with 'na'. 'D' and 'I' represents direct and indirect bandgaps.

Material	Ef (meV)	Bulk SG	1L SG	SCF-Bulk-Gap (eV)	BRIL-Bulk-Gap (eV)	SCF-1L-Gap (eV)	BRIL-1L-Gap (eV)	MPID	Ratio
Mg(AlSe2)2	35.419	R-3m	P-3m1	1.223 D	1.214 D	1.411 D	na	mp-9479	0.07
HfBrN	37.028	R-3m	P-3m1	1.865 D	1.83 D	2.146 D	na	mp-30302	0.07
TiNCl	39.396	Pmmn	Pmmn	0.608 D	0.592 D	0.616 D	0.603 D	mp-27850	0.08
SiH4	39.655	P2_1/c	Pc	6.144 D	6.153 D	2.304 D	2.278 D	mp-23739	0.34
InClO	40.036	Pmmn	Pmmn	2.416 D	2.388 D	2.704 D	2.403 D	mp-27702	0.07
Sc2CCl2	40.479	P-3m1	P-3m1	0.86 D	0.847 D	0.916 D	0.904 D	mp-28479	0.14
Sc2NCl2	40.87	P-3m1	P-3m1	0.0 I	0.0 I	0.0 I	0.0 I	mp-28480	0.09
TiBrN	41.273	Pmmn	Pmmn	0.616 D	0.607 D	0.624 D	0.615 D	mp-27849	0.07
TlSbO3	42.203	R-3	P-31m	2.398 D	2.415 D	2.917 D	2.819 D	mp-30058	0.06
Bi2TeI	43.941	P-1	P-3m1	0.0 I	0.207 D	0.0 I	na	mp-23435	0.09
TiIN	44.698	Pmmn	Pmmn	0.0 I	0.0 I	0.0 I	0.097 D	mp-27848	0.07
SiH	46.425	P-3m1	P-3m1	2.05 D	2.051 D	2.249 D	na	mp-29803	0.07
KMnP	47.5	P4/nmm	P4/nmm	0.0 I	0.0 I	0.0 I	0.0 I	mp-20422	0.37
RbMnAs	50.567	P4/nmm	P4/nmm	0.0 I	0.0 I	0.0 I	na	mp-20242	0.36
PrIO	51.832	P4/nmm	P4/nmm	3.686 D	3.619 D	3.862 D	3.788 D	mp-29254	0.08
Nb2CS2	51.867	R-3m	P-3m1	0.0 I	na	0.0 I	na	mp-4384	0.12
ScCl	51.953	R-3m	P-3m1	0.0 I	0.0 I	0.0 I	na	mp-27507	0.11
BiIO	52.167	P4/nmm	P4/nmm	1.576 D	1.574 D	1.424 D	1.581 D	mp-22987	0.07
ZrCl	52.187	C2/m	P-3m1	na	na	na	na	mp-27440	0.11
Ge(BiTe2)2	53.753	R-3m	P-3m1	0.61 D	0.571 D	1.048 D	na	mp-27948	0.11
ThIN	54.003	P4/nmm	P4/nmm	2.824 D	2.8 D	2.883 D	2.848 D	mp-28067	0.08

As2O3	54.29	P2_1/c	Pc	3.818 D	3.862 D	3.015 D	na	mp-1582	0.06
TiS3	54.493	P2_1/m	P2_1/m	0.306 D	0.309 D	0.337 D	0.338 D	mp-9920	0.08
CrS2	54.977	C2/m	C2/m	0.0 I	0.0 I	0.0 I	na	mp-28910	0.0
ZrS3	55.49	P2_1/m	P2_1/m	na	na	na	na	mp-9921	0.09
DySI	55.684	Pmmn	Pmmn	2.577 D	2.519 D	2.773 D	2.751 D	mp-28929	0.16
GaS	56.225	P6_3/mmc	P-6m2	1.5 D	1.46 D	2.45 D	2.428 D	mp-2507	0.12
BiBrO	56.424	P4/nmm	P4/nmm	2.293 D	2.244 D	2.54 D	na	mp-23072	0.1
GaS	57.085	R-3m	P-3m1	1.346 D	1.29 D	2.308 D	na	mp-9889	0.11
SmTe3	57.237	Cmcm	P4/nmm	0.0 I	0.0 I	0.0 I	na	mp-9399	0.06
PrTe3	57.251	Cmcm	P4/nmm	0.0 I	0.0 I	0.0 I	0.0 I	mp-12351	0.07
NdTe3	57.274	Cmcm	P4/nmm	0.0 I	0.0 I	0.0 I	0.0 I	mp-740	0.06
SrHI	57.392	P4/nmm	P4/nmm	3.608 D	3.354 D	4.05 D	2.167 D	mp-24205	0.11
NiO2	57.783	P-3m1	P-3m1	0.702 D	0.863 D	1.253 D	1.201 D	mp-715324	-0.01
US3	57.904	P2_1/m	P2_1/m	0.0 I	0.0 I	0.0 I	0.0 I	mp-12406	0.06
ZrSe3	58.413	P2_1/m	P2_1/m	na	na	na	na	mp-1683	0.07
GaSe	58.418	P6_3/mmc	P-6m2	0.725 D	0.698 D	1.84 D	1.852 D	mp-1943	0.11
TbBr	58.429	R-3m	P-3m1	0.0 I	0.0 I	0.0 I	na	mp-27924	0.11
LuSBr	58.962	Pmmn	Pmmn	2.856 D	2.841 D	1.565 D	3.007 D	mp-27246	0.2
DySBr	61.176	Pmmn	Pmmn	2.771 D	2.764 D	2.014 D	2.327 D	mp-28928	0.13
ErSeI	62.065	Pmmn	Pmmn	2.194 D	2.165 D	1.297 D	na	mp-28458	0.1
MgCl2	63.216	R-3m	P-3m1	5.641 D	5.575 D	na	5.762 D	mp-23210	0.11
TiS2	64.139	R-3m	P-3m1	0.0 I	0.0 I	0.0 I	na	mvc-11238	0.04
Sr3Si2	65.025	P6_3/mmc	P-3m1	0.0 I	0.0 I	0.0 I	0.0 I	mp-13051	0.13
ErSCl	65.078	Pmmn	Pmmn	2.844 D	2.833 D	3.036 D	3.026 D	mp-27616	0.09
GaTe	65.971	P6_3/mmc	P-6m2	0.163 D	0.093 D	1.483 D	na	mp-10009	0.09
TaI2O	66.308	C2/m	P2/m	0.872 D	0.911 D	0.96 D	na	mp-29027	0.09
ScPS4	66.628	P-1	P-1	1.737 D	1.756 D	1.905 D	1.922 D	mp-6999	0.13
TiCl2	66.703	P-3m1	P-3m1	0.0 I	0.0 I	0.0 I	na	mp-28116	0.16
CdPS3	67.223	C2/m	C2/m	1.761 D	1.692 D	1.956 D	1.95 D	mp-5328	0.11
FeCl2	67.411	R-3m	P-3m1	0.0 I	0.0 I	0.0 I	0.0 I	mp-23229	0.12

MnCl2	67.521	R-3m	P-3m1	1.553 D	1.49 D	1.659 D	1.656 D	mp-28233	0.1
Bi2Te2S	67.683	R-3m	P-3m1	0.304 D	na	0.968 D	0.98 D	mp-27910	0.06
InSe	68.03	R3m	P3m1	0.174 D	0.143 D	1.469 D	1.461 D	mp-22691	0.07
Nb(SCl)2	68.522	C2/m	C2/m	1.457 D	1.536 D	1.665 D	na	mp-27362	0.11
USe3	69.04	P2_1/m	P2_1/m	0.0 I	0.0 I	0.0 I	0.0 I	mp-9429	0.07
CdPS3	69.104	R-3	P-3	1.808 D	1.753 D	1.963 D	na	mp-9330	0.1
ScCl3	69.313	R-3	P-3	3.535 D	3.527 D	3.784 D	3.783 D	mp-23309	0.07
ZrCl2	69.383	R3m	P-6m2	na	na	na	na	mp-23162	0.06
Nb(SeCl)2	69.421	P-1	P-1	0.967 D	0.887 D	1.089 D	na	mp-27361	0.1
CrCl3	69.523	C2/m	C2/m	1.493 D	1.467 D	1.692 D	na	mp-27630	0.08
ScAg(PS3)2	69.558	P-31c	P312	1.9 D	1.892 D	2.012 D	1.991 D	mp-8616	0.13
C	70.355	P6_3/mmc	P6/mmm	0.0 I	0.0 I	0.0 I	0.0 I	mp-48	0.13
Bi2Te2Se	70.529	R-3m	P-3m1	0.23 D	0.225 D	0.909 D	0.92 D	mp-29666	0.08
CdCl2	70.634	R-3m	P-3m1	3.342 D	3.268 D	3.893 D	2.185 D	mp-22881	0.08
BiClO	71.047	P4/nmm	P4/nmm	2.87 D	2.714 D	2.898 D	2.637 D	mp-22939	0.06
BN	71.336	P6_3/mmc	P-6m2	4.46 D	4.096 D	4.484 D	4.212 D	mp-984	0.15
HfCl4	71.624	P2/c	P2/c	3.912 D	3.895 D	2.916 D	4.096 D	mp-29422	0.08
HfFeCl6	71.851	P-31c	P312	0.0 I	0.083 D	1.369 D	na	mp-28220	0.09
RhCl3	71.894	C2/m	C2/m	1.481 D	1.441 D	1.728 D	na	mp-27770	0.08
MgPSe3	71.899	R-3	P-3	2.067 D	2.057 D	2.09 D	2.086 D	mp-30943	0.09
MgBr2	71.93	P-3m1	P-3m1	4.479 D	4.43 D	3.164 D	2.39 D	mp-30034	0.12
ThBrN	72.054	P4/nmm	P4/nmm	2.647 D	na	2.806 D	1.81 D	mp-28066	0.16
Nb3Cl8	72.972	P-3m1	P3m1	0.0 I	0.0 I	0.0 I	0.0 I	mp-29950	0.09
Nb3TeCl7	72.98	P-3m1	P3m1	0.698 D	0.666 D	0.754 D	0.756 D	mp-28938	0.09
VCl3	73.316	R-3	P-31m	0.0 I	0.0 I	0.0 I	0.0 I	mp-28117	0.1
IrCl3	73.514	C2/m	C2/m	1.793 D	1.794 D	2.099 D	2.089 D	mp-27666	0.09
OsCl2O	73.847	Immm	Pmmm	0.0 I	0.0 I	0.0 I	0.0 I	mp-29133	0.12
MnBr2	74.126	P-3m1	P-3m1	1.419 D	1.355 D	1.464 D	1.403 D	mp-28306	0.13
TcS2	74.425	P-1	P-1	0.929 D	0.946 D	1.259 D	1.245 D	mp-9481	0.13
GaAg(PSe3)2	74.54	P-31c	P312	0.362 D	0.353 D	na	na	mp-7008	0.12

ReSe2	75.101	P-1	P-1	1.069 D	1.033 D	1.247 D	1.21 D	mp-541582	0.07
ScAg(PSe3)2	75.372	P-31c	P312	1.481 D	1.462 D	1.651 D	1.65 D	mp-13383	0.14
CoBr2	75.463	P-3m1	P-3m1	0.0 I	0.0 I	0.0 I	0.0 I	mp-30033	0.13
CS2	75.879	Cmce	C2/m	2.881 D	2.881 D	1.691 D	na	mp-2232	0.09
CrSe	75.921	P4/nmm	P4/nmm	0.0 I	0.0 I	0.0 I	0.0 I	mp-604915	0.08
TiBr2	75.929	P-3m1	P-3m1	0.0 I	0.0 I	0.0 I	na	mp-27785	0.23
CdBr2	76.006	P6_3mc	P3m1	2.742 D	2.676 D	2.564 D	na	mp-27934	0.13
WS2	76.272	P6_3/mmc	P-6m2	0.718 D	1.024 D	1.337 D	1.606 D	mp-224	0.15
SnO2	76.452	P1	P1	1.623 D	1.6 D	2.214 D	2.613 D	mvc-6946	0.03
MoS2	76.991	P6_3/mmc	P-6m2	0.922 D	0.919 D	1.658 D	1.714 D	mp-2815	0.21
TmAg(PSe3)2	77.551	P-31c	P312	1.667 D	1.646 D	1.79 D	1.785 D	mp-13385	0.12
BiI	77.687	C2/m	C2/m	0.557 D	1.282 D	0.711 D	na	mp-27708	0.08
ErAg(PSe3)2	77.708	P-31c	P312	1.651 D	1.63 D	1.473 D	1.762 D	mp-13384	0.14
SNCl	77.946	P2_1/m	Pm	2.08 D	2.078 D	2.759 D	na	mp-27952	0.06
Hf3Te2	78.34	I4/mmm	P4/mmm	0.0 I	0.0 I	0.0 I	0.0 I	mp-28919	0.07
InAg(PSe3)2	78.845	P-31c	P312	0.463 D	0.437 D	na	0.853 D	mp-20902	0.09
CrBr3	79.078	R-3	P-31m	1.299 D	1.272 D	1.47 D	1.469 D	mp-27734	0.13
WSe2	79.634	P6_3/mmc	P-6m2	1.046 D	1.002 D	1.334 D	1.546 D	mp-1821	0.16
PtO2	79.793	P6_3mc	P-3m1	1.47 D	1.315 D	1.788 D	1.714 D	mp-7868	0.13
PPdS	79.815	Pbcn	P2/c	0.903 D	0.876 D	1.242 D	1.202 D	mp-7280	0.12
MoSe2	80.237	P6_3/mmc	P-6m2	0.906 D	0.873 D	1.446 D	1.481 D	mp-1634	0.2
RhBr3	80.673	C2/m	C2/m	1.167 D	1.129 D	1.487 D	na	mp-27871	0.07
IrBr3	81.288	C2/m	C2/m	1.423 D	1.583 D	1.797 D	1.785 D	mp-27397	0.1
Al2Te3	81.669	P2_1/c	P2_1/c	1.776 D	1.787 D	1.874 D	1.858 D	mp-29502	0.08
AlSiTe3	81.933	P-31m	P-31m	1.206 D	1.145 D	1.522 D	1.426 D	mp-31220	0.1
MgI2	82.056	P-3m1	P-3m1	3.625 D	3.551 D	3.795 D	3.738 D	mp-23205	0.12
MnI2	82.287	P-3m1	P-3m1	1.047 D	0.962 D	1.044 D	1.006 D	mp-28013	0.06
BPS4	82.402	I222	P222	1.932 D	1.989 D	2.104 D	2.386 D	mp-27724	0.13
CaI2	82.422	P-3m1	P-3m1	3.446 D	3.406 D	1.237 D	3.806 D	mp-30031	0.16
S5N6	82.791	C2/c	C2	2.014 D	2.015 D	2.067 D	na	mp-1419	0.07

PPdSe	83.189	Pbcn	P2/c	0.716 D	0.723 D	1.079 D	1.083 D	mp-3123	0.1
SnS2	83.279	P-3m1	P-3m1	1.224 D	1.17 D	1.553 D	1.418 D	mp-1170	0.18
VSe2	83.778	P-3m1	P-3m1	0.0 I	0.0 I	0.0 I	0.0 I	mp-694	0.14
AlPS4	83.989	P222	P222	2.48 D	2.463 D	1.695 D	1.149 D	mp-27462	0.27
CdI2	84.257	P6_3mc	P-3m1	2.119 D	2.055 D	1.531 D	1.243 D	mp-28248	0.09
PtO2	84.508	P-3m1	P-3m1	1.51 D	1.528 D	1.788 D	1.788 D	mp-617	0.13
Te2W	84.899	Pmn2_1	P2_1/m	0.0 I	0.0 I	0.0 I	0.0 I	mp-22693	0.1
SiS2	85.991	Ibam	Pccm	2.727 D	2.727 D	2.702 D	1.371 D	mp-1602	0.11
P4S5	86.241	P2_1/m	Pm	2.227 D	2.217 D	2.433 D	na	mp-7260	0.12
GeI2	86.265	P-3m1	P3m1	2.062 D	2.129 D	na	2.175 D	mp-27922	0.1
VS2	86.275	P-3m1	P-3m1	0.0 I	0.0 I	0.0 I	0.0 I	mp-9561	0.14
HfS2	86.831	P-3m1	P-3m1	1.078 D	0.944 D	1.345 D	1.256 D	mp-985829	-0.0
Te2Mo	86.842	Pmn2_1	P2_1/m	0.0 I	0.0 I	0.0 I	0.0 I	mp-MoTe2Td	na
Te2Mo	86.87	P2_1/m	P2_1/m	0.0 I	0.0 I	0.0 I	0.0 I	mp-7459	0.12
Re(AgCl3)2	86.921	R-3	P-3	0.591 D	0.59 D	0.737 D	0.736 D	mp-23472	0.06
CrSiTe3	87.051	R-3	P-3	0.553 D	0.537 D	0.558 D	0.554 D	mp-3779	0.08
TaS2	87.155	P6_3/mmc	P-6m2	0.0 I	0.0 I	0.0 I	0.0 I	mp-1984	0.14
PbI2	87.357	R-3m	P-3m1	2.272 D	2.194 D	2.653 D	2.63 D	mp-22883	0.13
TiO2	88.017	P6_3/mmc	P6/mmm	0.0 I	0.0 I	0.0 I	0.0 I	mvc-13391	0.06
TaSe2	88.522	R3m	P-6m2	0.0 I	0.0 I	0.0 I	0.0 I	mp-13870	0.08
SbBr3	88.648	P2_12_12_1	Pmn2_1	2.851 D	2.828 D	2.943 D	na	mp-27399	0.15
AuI	88.81	P4_2/ncm	Cmme	1.404 D	1.365 D	1.747 D	1.709 D	mp-27725	0.09
ZrS2	88.919	P-3m1	P-3m1	na	na	na	na	mp-1186	0.13
PbI2	88.934	P-3m1	P-3m1	2.242 D	2.18 D	2.652 D	2.572 D	mp-22893	0.07
MoS2	89.425	R-3m	C2/m	0.0 I	0.0 I	0.0 I	0.0 I	mp-558544	0.01
TiS2	89.569	P-3m1	C2/m	0.0 I	0.0 I	0.0 I	0.089 D	mp-558110	-0.0
TiS2	89.718	P-3m1	P-3m1	0.0 I	0.0 I	0.0 I	0.095 D	mp-2156	0.13
AlSeBr7	89.921	Pc	P1	2.135 D	2.146 D	1.331 D	1.717 D	mp-29408	0.07
TaSe2	90.692	P6_3mc	P-6m2	0.0 I	0.0 I	0.0 I	0.0 I	mp-7926	0.12
TmI2	90.774	P-3m1	P-3m1	0.0 I	0.0 I	0.0 I	na	mp-29671	0.16

Te2Mo	90.981	P6_3/mmc	P-6m2	0.765 D	0.754 D	1.229 D	1.107 D	mp-602	0.1
TaSe2	91.53	P6_3/mmc	P-6m2	0.0 I	0.0 I	0.0 I	0.0 I	mp-500	0.14
TaS2	91.882	P-3m1	P-3m1	0.0 I	0.0 I	0.0 I	0.0 I	mp-1690	0.18
HfSe2	92.049	P-3m1	P-3m1	0.534 D	0.441 D	0.693 D	0.62 D	mp-985831	0.05
SiTe2	92.34	P-3m1	P-3m1	0.0 I	0.0 I	0.0 I	0.0 I	mp-2755	0.68
NbS2	92.929	P6_3/mmc	P-6m2	0.0 I	0.0 I	0.0 I	0.0 I	mp-10033	0.13
SnSe2	93.474	P-3m1	P-3m1	0.471 D	0.411 D	0.731 D	0.665 D	mp-665	0.12
TiSe2	94.485	P-3m1	P-3m1	0.0 I	0.0 I	0.0 I	0.0 I	mp-2194	0.11
Ta3TeI7	94.752	P6_3mc	P3m1	0.529 D	0.528 D	0.659 D	na	mp-29117	0.08
MoBr3	95.031	Pmmn	Pmm2	0.578 D	0.578 D	0.562 D	0.565 D	mp-23312	0.11
ZrSe2	95.444	P-3m1	P-3m1	na	na	na	na	mp-2076	0.08
PtS2	95.494	P-3m1	P-3m1	0.721 D	0.708 D	1.626 D	1.813 D	mp-762	0.25
AlTeI7	95.514	Pc	P1	1.774 D	1.775 D	1.579 D	1.583 D	mp-29407	0.08
PCl3	96.07	Pnma	Pmc2_1	3.771 D	3.732 D	1.888 D	1.689 D	mp-23230	0.08
BiI3	96.214	R-3	P-3	2.365 D	2.353 D	2.548 D	2.537 D	mp-22849	0.09
TaSe2	96.411	P-3m1	P-3m1	0.0 I	0.0 I	0.0 I	0.0 I	mp-11324	0.09
PSe	96.67	P2_1/c	P2_1	1.971 D	2.039 D	1.224 D	na	mp-28885	0.12
Nb3TeI7	97.529	P6_3mc	P3m1	0.504 D	0.502 D	0.606 D	0.609 D	mp-29689	0.08
SbI3	97.593	R-3	P-3	2.061 D	2.029 D	2.202 D	2.202 D	mp-23281	0.12
NbSe2	98.266	P6_3/mmc	P-6m2	0.0 I	0.0 I	0.0 I	0.0 I	mp-2207	0.1
NdI2	98.598	I4/mmm	P4/mmm	0.0 I	0.0 I	0.0 I	0.0 I	mp-28753	0.11
RuBr3	99.687	P6_3/mcm	Pmma	0.0 I	0.0 I	0.0 I	0.0 I	mp-23294	0.11
AlBr3	100.052	P2_1/c	P-1	3.974 D	3.973 D	1.217 D	3.133 D	mp-23288	0.1
SnO	100.684	P4/nmm	P4/nmm	0.505 D	0.428 D	3.025 D	3.085 D	mp-2097	0.04
Te2Br	101.538	Pnma	Pmn2_1	0.152 D	0.0 I	0.881 D	0.78 D	mp-27648	0.12
WO2	102.658	P6_3/mmc	P6/mmm	0.0 I	0.0 I	0.0 I	0.0 I	mvc-11221	0.01
BiTeI	103.58	P3m1	P3m1	1.2 D	1.131 D	1.617 D	1.575 D	mp-22965	0.08
BiTeCl	104.208	P6_3mc	P3m1	1.217 D	1.159 D	1.817 D	1.806 D	mp-28944	0.14
TlPt2S3	106.049	P-3m1	P-3m1	1.064 D	0.961 D	1.648 D	1.614 D	mp-9272	0.06
Te2I	106.926	Pnma	Pmn2_1	0.213 D	0.133 D	0.923 D	0.901 D	mp-27655	0.12

PtSe2	107.579	P-3m1	P-3m1	0.0 I	0.0 I	1.362 D	1.347 D	mp-1115	0.21
HfTe2	108.003	P-3m1	P-3m1	0.0 I	0.0 I	0.0 I	0.0 I	mp-32887	0.75
BCl3	108.178	P6_3/m	P-6	4.531 D	4.48 D	4.761 D	4.696 D	mp-23184	0.17
HgI2	109.315	P4_2/nmc	P-4m2	0.969 D	0.95 D	1.752 D	1.727 D	mp-23192	0.15
PBr3	109.671	Pnma	Pmc2_1	2.992 D	2.992 D	3.332 D	3.236 D	mp-27257	0.09
TiTe2	109.772	P-3m1	P-3m1	0.0 I	0.0 I	0.0 I	0.0 I	mp-1907	0.04
BiSBr	110.97	Pnma	P2_1/m	1.945 D	1.999 D	1.426 D	1.679 D	mp-23324	0.07
CaN	111.204	P-3m1	P-3m1	1.982 D	1.851 D	0.0 I	na	mp-13146	0.28
TiI3	111.232	Pmmn	Pmm2	0.0 I	0.0 I	0.192 D	0.0 I	mp-23264	0.09
Mn2Bi	111.333	P6_3/mmc	P-3m1	0.0 I	0.0 I	0.0 I	na	mp-22878	0.13
P	111.624	Cmce	Pmna	0.0 I	0.0 I	0.849 D	0.839 D	mp-blackP	na
SrThBr6	115.676	Pmma	Pmm2	2.75 D	2.749 D	2.026 D	na	mp-29016	0.1
AlI3	120.514	P2_1/c	P-1	3.017 D	3.011 D	2.945 D	2.549 D	mp-30930	0.09
P2Se5	122.456	P2_1/c	P1	1.049 D	1.07 D	1.125 D	1.288 D	mp-28860	0.09
SbSBr	124.352	Pnma	P2_1/m	1.71 D	1.744 D	1.589 D	1.51 D	mp-22971	0.07
TlPd2Se3	124.918	P-3m1	P-3m1	0.0 I	0.0 I	1.135 D	0.961 D	mp-7038	0.06
BBr3	125.359	P6_3/m	P-6	3.688 D	3.61 D	3.808 D	3.729 D	mp-23225	0.2
TlTe3Pt2	138.243	P-3m1	P-3m1	0.081 D	0.0 I	1.118 D	0.986 D	mp-9251	0.07
Ta(ICl)2	142.131	Immm	Pmmm	1.05 D	1.039 D	1.302 D	na	mp-28683	0.06
Te2Pt	144.322	P-3m1	P-3m1	0.0 I	0.0 I	0.729 D	0.675 D	mp-399	0.03
PdS2	145.066	Pbca	P2_1/c	0.0 I	0.0 I	1.282 D	1.261 D	mp-13682	0.14
BI3	154.542	P6_3/m	P-62m	2.651 D	2.58 D	2.681 D	2.587 D	mp-23189	0.18
PdSe2	166.236	Pbca	P2_1/c	0.0 I	0.0 I	1.437 D	1.442 D	mp-2418	0.12
NiTe2	172.015	P-3m1	P-3m1	0.0 I	0.0 I	0.0 I	0.0 I	mp-2578	0.0
NbI5	178.347	P2_1/c	Pc	0.542 D	na	0.945 D	na	mp-31487	0.1
PI3	184.599	P6_3	P3	1.972 D	1.9 D	2.334 D	na	mp-27529	0.14
BaBrCl	195.643	Pnma	P2_1/m	4.761 D	4.747 D	2.466 D	na	mp-28049	0.09
ZrS	199.719	P4/nmm	P4/nmm	na	na	na	na	mp-7859	0.1
Te2Pd	202.392	P-3m1	P-3m1	0.0 I	na	0.155 D	0.153 D	mp-782	0.01
Te2Ir	211.103	P-3m1	P-3m1	0.0 I	0.0 I	0.0 I	0.0 I	mp-2285	0.01

BiSe2	214.855	P-3m1	P-3m1	0.0 I	0.0 I	0.0 I	na	mp-27902	0.06
BiSI	221.479	Pnma	P2_1/m	1.767 D	1.725 D	2.063 D	na	mp-23514	0.09
SbSeI	236.632	Pnma	Pmn2_1	1.363 D	1.339 D	1.189 D	1.195 D	mp-22996	0.07
Bi2Te3	250.821	R-3m	P3m1	0.351 D	0.328 D	0.383 D	na	mp-34202	0.02
VS2	298.243	P6_3/mmc	P-6m2	0.0 I	0.0 I	0.0 I	na	mp-1214	0.11
CaSn	337.877	Cmcm	Pmma	0.0 I	0.0 I	0.0 I	0.0 I	mp-2450	0.14
KAuSe	338.351	Cmcm	Pmma	2.046 D	1.896 D	1.895 D	1.877 D	mp-9881	0.09
KAuS	343.839	Cmcm	Pmma	2.296 D	2.173 D	1.9 D	1.943 D	mp-7077	0.1
RbAuSe	345.213	Cmcm	Pmma	1.966 D	1.83 D	1.823 D	1.809 D	mp-9731	0.08
RbAuS	354.167	Cmcm	Pmma	2.173 D	2.054 D	1.324 D	2.03 D	mp-9010	0.08
Sc2C	423.692	P-3m1	P-3m1	0.0 I	0.0 I	0.0 I	0.0 I	mp-29941	0.15
TiSe2	498.526	P6_3/mmc	P-6m2	0.0 I	0.0 I	0.66 D	0.778 D	mp-10027	0.16
GaN	499.825	P6_3mc	P-6m2	1.943 D	1.85 D	2.259 D	2.139 D	mp-804	0.08
Sr2H3	550.557	P6_3/mmc	P-3m1	1.678 D	1.456 D	0.0 I	na	mp-23759	0.08
Ca2H3	557.151	P6_3/mmc	P-3m1	1.055 D	0.783 D	0.0 I	na	mp-24809	0.14
AlN	626.819	P6_3mc	P-6m2	4.474 D	4.417 D	2.988 D	na	mp-661	0.01
TiTe2	644.016	P6_3/mmc	P-3m1	0.0 I	0.0 I	0.0 I	na	mp-567832	0.14
CrSe2	675.698	P6_3/mmc	P-3m1	0.0 I	0.0 I	0.0 I	0.0 I	mp-2189	0.02
ZrTe2	697.341	P6_3/mmc	P-6m2	na	na	na	na	mp-1319	0.23
CoAs2	702.752	P6_3/mmc	P-3m1	0.0 I	0.0 I	0.0 I	na	mp-15679	0.09
Ti2O	946.438	P-3m1	P-3m1	0.0 I	0.0 I	0.0 I	0.0 I	mp-1215	0.02
SnO2	1296.062	P6_3/mmc	P6/mmm	0.0 I	0.0 I	2.134 D	2.119 D	mvc-13245	0.09
GaSe	3020.593	P6/mmm	P-6m2	0.0 I	0.0 I	1.868 D	na	mp-1566	0.06

Table S7: Comparison of functionals for single layer $MoTe_2$ 2H structure. Suffix r stands for previously reported values. C11 is the elastic constant and Φ is the work function of materials.

Mat.	SG	Functional	c_{11}	c_{11r} [1]	Φ	Φ r [2]
MoS2	P-6m2	LDA	146.9	138.1	-5.32	-
MoS2	P-6m2	PBE	132.6	-	-5.09	-5.07
MoS2	P-6m2	OptB	134.1	-	-5.43	-
WSe2	P-6m2	LDA	133.7	130.04	-4.72	-
WSe2	P-6m2	PBE	119.5	-	-4.32	-4.21
WSe2	P-6m2	OptB	121.5	-	-4.83	-
MoSe2	P-6m2	LDA	124.0	118.37	-4.77	-
MoSe2	P-6m2	PBE	109.7	-	-4.60	-4.57
MoSe2	P-6m2	OptB88	111.8	-	-4.96	-
MoTe2	P-6m2	LDA	96.0	92.7	-4.56	-
MoTe2	P-6m2	PBE	83.0	-	-4.33	-4.29
MoTe2	P-6m2	OptB	84.8	-	-4.65	-
WS2	P-6m2	LDA	159.1	151.48	-5.29	-
WS2	P-6m2	PBE	145.3	-	-4.86	-4.73
WS2	P-6m2	OptB	146.5	-	-5.41	-

Table S8: Effect of simulation cell-size on elastic constants of materials

Material	C11	C12	C13	C22	C33	C44	C66
BN ($P6_3/mmc$)	883.7	198.3	1.1	883.7	36.9	342.6	5.5
BN ($P6_3/mmc$),2x2x2	883.6	198.3	1.2	883.7	36.9	342.3	5.3
BiClO(P4/nmm)	135.4	69.4	36.9	135.4	51.0	59.5	25.7
BiClO(P4/nmm), 2x2x2	135.6	69.4	36.9	135.6	51.9	59.4	25.6
AuI ($P4_2/ncm$)	24.9	9.0	13.2	24.9	24.5	4.0	8.4
AuI ($P4_2/ncm$), 2x2x2	24.9	9.0	13.2	24.9	24.6	4.0	8.4

References:

[1] C. Ataca, H. Sahin, and S. Ciraci, "Stable, single-layer MX2 transition-metal oxides and dichalcogenides in a honeycomb-like structure," *The Journal of Physical Chemistry C,* vol. 116, pp. 8983-8999, 2012

[2] C. Gong, H. Zhang, W. Wang, L. Colombo, R. M. Wallace, and K. Cho, "Band alignment of two-dimensional transition metal dichalcogenides: Application in tunnel field effect transistors," *Applied Physics Letters,* vol. 103, p. 053513, 2013
